# Chronic Stress Attenuates the Glucocorticoid Receptor Transcriptional Programme in the *Danio rerio* Telencephalon

**DOI:** 10.3390/ijms27219772

**Published:** 2026-10-30

**Authors:** Maksim V. Shamshurin, Maksim A. Grabchuk, Vladislav A. Shatilov, Olga B. Tseilikman, Vadim E. Tseilikman

**Affiliations:** Faculty of Fundamental Medicine, Chelyabinsk State University, 129 Bratiev Kashirinykh St., Chelyabinsk 454001, Russia; narzgulus@gmail.com (M.V.S.); grabchukm.a@gmail.com (M.A.G.);

**Keywords:** *Danio rerio*, chronic unpredictable stress, telencephalon, glucocorticoid receptor, associative gene network, gene expression, transcriptomic data reanalysis

## Abstract

We reanalysed public RNA-seq data from the telencephalon of adult zebrafish after 14 days of chronic unpredictable stress. The deposited runs concatenate pooled libraries, hiding the replicates; we recovered the library boundaries from the block structure of unaligned reads and realigned to GRCz11 with splicing enabled. Unique alignment rose from 63.7–64.2% to 80.6–81.6%, and 275 genes were differentially expressed (FDR < 0.05), 101 of the 155 reported previously and 174 new, with effect sizes matching the published ones (*ρ* = 0.754). Against gene sets from an *nr3c1* knockout, the data show a coordinated, mirror-symmetric shift in receptor-dependent transcription—the receptor-induced set down, the receptor-repressed set up (*p* ≈ 0.001 for both)—while the original study reported cortisol at control level; “receptor-dependent” denotes set membership, not receptor occupancy. Two artefacts constrain the reading of individual genes: epidermal content differs between groups while neuronal content does not, and the gene list is clustered in the genome far beyond chance. Removing the 85 flagged genes (of 275) leaves the receptor-level result intact (*p* = 0.0008 and 0.0014) but withdraws the conclusions on keratins and barrier structures. That removal addresses gene-level artefact, not sample composition, which six libraries cannot settle: a between-group difference in tissue composition remains an alternative explanation.

## 1. Introduction

Chronic unpredictable stress (CUS) in *Danio rerio* reproduces behavioural, endocrine and transcriptomic features of affective disorders and post-traumatic stress disorder (PTSD) [1,2,3] and is effective even at early developmental stages [4]. The species is established in translational neuroscience [5]: about 70% of human genes have orthologues in it [6], its pallium contains putative homologues of the hippocampus and amygdala [7], and cortisol acts through a conserved glucocorticoid receptor (GR) whose null mutants are viable [8].

In mammals, prolonged glucocorticoid action remodels hippocampal and prefrontal circuits [9,10], suppresses neurogenesis [11] and alters mTORC1 signalling through REDD1/DDIT4 [12]. Receptor output depends on cofactors such as FKBP5 [13,14], and its genomic targets have been mapped [15]. Unlike depression, PTSD often shows low basal cortisol with enhanced negative feedback [16,17].

In *D. rerio*, a five-week CUS protocol raises brain GR expression without changing cortisol, which falls only by week twelve; blood–brain barrier permeability rises and later normalises as glial markers shift from a pro- to an anti-inflammatory profile [3]. Mitochondrial proteins of the brain proteome also change [18]. Stress models further show altered monoamine oxidase activity, lipid peroxidation and mitochondrial function [19,20,21,22,23]. These are measures of enzyme activity; transcriptomic data describe a different level of regulation and complement them.

The telencephalon transcriptome under a 14-day protocol was reported by Huang et al. [1]. In the archive, however, the three pooled libraries of each group are merged into one run without read groups, so the replicates are invisible to standard tools, and the reads were aligned to the outdated GRCz10 assembly with spliced alignment disabled.

STRING-based network analysis has been applied to this species before [2]. STRING edges are undirected and reflect association rather than regulation; following Kolchanov et al. [24], we therefore speak of an associative gene network, draw no conclusions about direction, and test gene positions against null models that control for how well genes are studied.

The aim of this work was to recover the replicate structure of these data and to ask which functional groups of genes change coordinately, what happens to the GR-dependent programme, and how specific the resulting profile is relative to neuroinflammation and to CUS of a different duration.

## 2. Results

### 2.1. Library Recovery and Realignment

The archive loader numbers the aligned spots of each submitted file consecutively and places that file’s unaligned spots at the end of its own block, so a run assembled from several files carries an internal signature: a short stretch of fully unaligned read pairs at each junction. Each of the two deposited runs contains three such stretches, and their positions therefore mark the boundaries of the three files from which the run was assembled (Table 1).

Six checks support the partition, and they test two different things. Three concern the arithmetic of the split: library sizes sum exactly to the number of spots in each run with zero discrepancy, the read counts of the extracted libraries match the expected values exactly, and alignment identifiers continue without a gap across each boundary. Three concerns the correspondence between the blocks and the submitted files: the number of blocks equals the number of source BAM files listed in the SRA record, the relative library sizes follow the order of the source file sizes, and within each block the alignments run from chromosome 1 to the last scaffold, as they would in a separately sorted file. The aligned fraction is uniform across the recovered libraries. Spliced realignment to GRCz11 raised the uniquely aligned fraction to 80.6–81.6% (Figure S1), with within-group spread below one percentage point.

### 2.2. What the Recovered Partition Establishes

The checks above establish that each deposited run is a concatenation of three separately prepared files and that we have located the boundaries between them. They do not establish that those three files are independent biological pools rather than repeated sequencing of one pool; that follows from the design of the source study, which states that four telencephala were pooled per sample and three samples were prepared per group, and it is an inference from that description rather than a property we have measured. Three observations are consistent with it without proving it, and we report them as such: libraries of the same group correlate more closely with one another than with libraries of the other group (mean Spearman ρ 0.9883 against 0.9846; Table S1b, Figure S2b), which is the ordering expected of biological replicates and not the pattern repeated sequencing of one pool would give; asymptotic dispersion was 0.0056 and median per-gene dispersion 0.0041, higher than the near-zero values typical of technical replicates, and the first principal component (48.4% of variance) separates the groups completely (Table S1, Figure S2). Neither measure can identify the biological origin of a library, and the consequences of this limit are set out in Section 3.9.

### 2.3. Differential Expression

We identified 275 DEGs at FDR < 0.05, 139 up- and 136 down-regulated (Figures S3 and S4), against 155 in the original publication (Table 2). Effect-size estimates are reproduced (Spearman ρ = 0.754 across the 15,690 genes with baseMean ≥ 10 in both analyses; Figure S5), and of the 134 previously reported DEGs in that set, 132 agree in direction. Without the expression filter, 140 of the 155 published DEGs receive an adjusted *p*-value here, and 138 of them agree in direction. An independent computation with PyDESeq2 gave 274 DEGs, 257 shared, with a log_2_FC correlation of 1.000.

The most significant additionally identified genes are listed in Table S2; the strongest is the histone methyltransferase *prdm2a* (+5.12; *padj* = 4.7 × 10^−23^). Among genes significant in both analyses, *chrna7a*, which encodes the α7 nicotinic receptor subunit (*chrna7* in the annotation of [1]), gained significance (*padj* = 3.2 × 10^−4^ against 2.3 × 10^−2^). ↑—indicates upregulated gene expression; ↓—indicates downregulated gene expression.

### 2.4. Isoform Specificity of Collagen Changes

Matrix changes affect individual isoforms and paralogues rather than whole families. Of six type IV collagen genes, only *col4a6* changes (+0.98); of four laminin beta chains, only *lamb2l* (+0.78); of two lysyl oxidase-like paralogues, only *loxl2b* (−2.80), while *loxl2a* is unchanged. Nidogens, perlecan, fibronectins and *sparc* are unaffected. Within collagen maturation, directions diverge: *p3h1* (−1.04) and *loxl2b* are reduced, the procollagen C-proteinase *bmp1b* is elevated (+0.64), and both type XVII collagen genes, hemidesmosome components, fall sharply (*col17a1a* −6.26, *col17a1b* −2.72).

Perineuronal nets, discussed as a substrate for the persistence of fear memories [25], are unaffected: none of the 17 tested components changed (all |log_2_FC| < 0.7; Table S3). The changes identified belong to the basement membrane, not to this hyaluronan-based matrix.

One qualification belongs here. *col17a1a* is reassigned in Section 2.11 as a transcript of probable epidermal origin, and after its exclusion the basement-membrane term is no longer enriched; the collagen-trimer term is. The isoform specificity described above therefore stands for the collagens that remain, and the basement membrane as such is not established by these data.

### 2.5. Systems Regulated Mainly Downstream of Transcription

Of the antioxidant defence system, only *gpx1a* changes (−0.67); superoxide dismutases, catalase, peroxiredoxins, the thioredoxin system, *nfe2l2a* and *keap1a* do not (Table S3). The single monoamine oxidase gene *mao*, as well as *nr3c1*, *nr3c2* and *hsd11b2*, is unchanged.

### 2.6. Glutamine Transport and Nucleotide Metabolism

Two transporters change in opposite directions with high significance: the glial glutamine transporter *slc38a3a* is reduced (−0.73; *padj* = 4.9 × 10^−7^) and the neuronal amino acid transporter *slc6a17* is elevated (+1.11; *padj* = 6.3 × 10^−8^). Glutamine synthetases and glutamate and vesicular transporters are unchanged. In cluster MCL 5, the changed genes maintain the mitochondrial deoxyribonucleotide pool (*tk2* −0.71, *rrm2b* +0.69) or catabolise adenine nucleotides (*ampd1* +3.73); of the ectonucleotidases, only *entpd8* changes, and purinergic receptors are unaffected.

### 2.7. The Associative Gene Network

Of the 275 DEGs, 256 mapped to STRING. At 0.400, with all sources, 137 edges link 118 genes, the largest component comprising 61 nodes; the remaining 138 genes are isolated, reflecting the sparse annotation of the *D. rerio* interactome. The 50 interactor proteins add 368 edges but no new edges between DEGs, so they were excluded. Sensitivity to threshold and evidence sources is given in Table S4.

Textmining contributes to 123 of 137 edges; 33 edges have no support outside it. Removing these leaves 104 edges and splits the largest component into 38, 11 and 6 nodes; this operation was used to assess cluster robustness.

Connectivity of the query list exceeds chance only under study-bias control (Table S5). Random sets matched for node degree give 64.8 expected internal edges (95% interval 50–80) against 137 observed (*p* < 0.001). Without matching, no effect is detected (*p* = 0.219), because DEGs are less studied than the background (median degree 31 against 41).

### 2.8. Node Connectivity

Degree ranges from one to eight (mean 2.3). For no node does betweenness centrality exceed its expectation under 1000 degree-preserving randomisations (Table S6); for most it falls below. High-degree genes thus sit inside dense clusters rather than bridging them. The highest-degree node, *cdh1* (E-cadherin, −2.50; degree 8), belongs to no cluster of five or more nodes.

### 2.9. Clusters and Their Functional Enrichment

**Figure 1:**
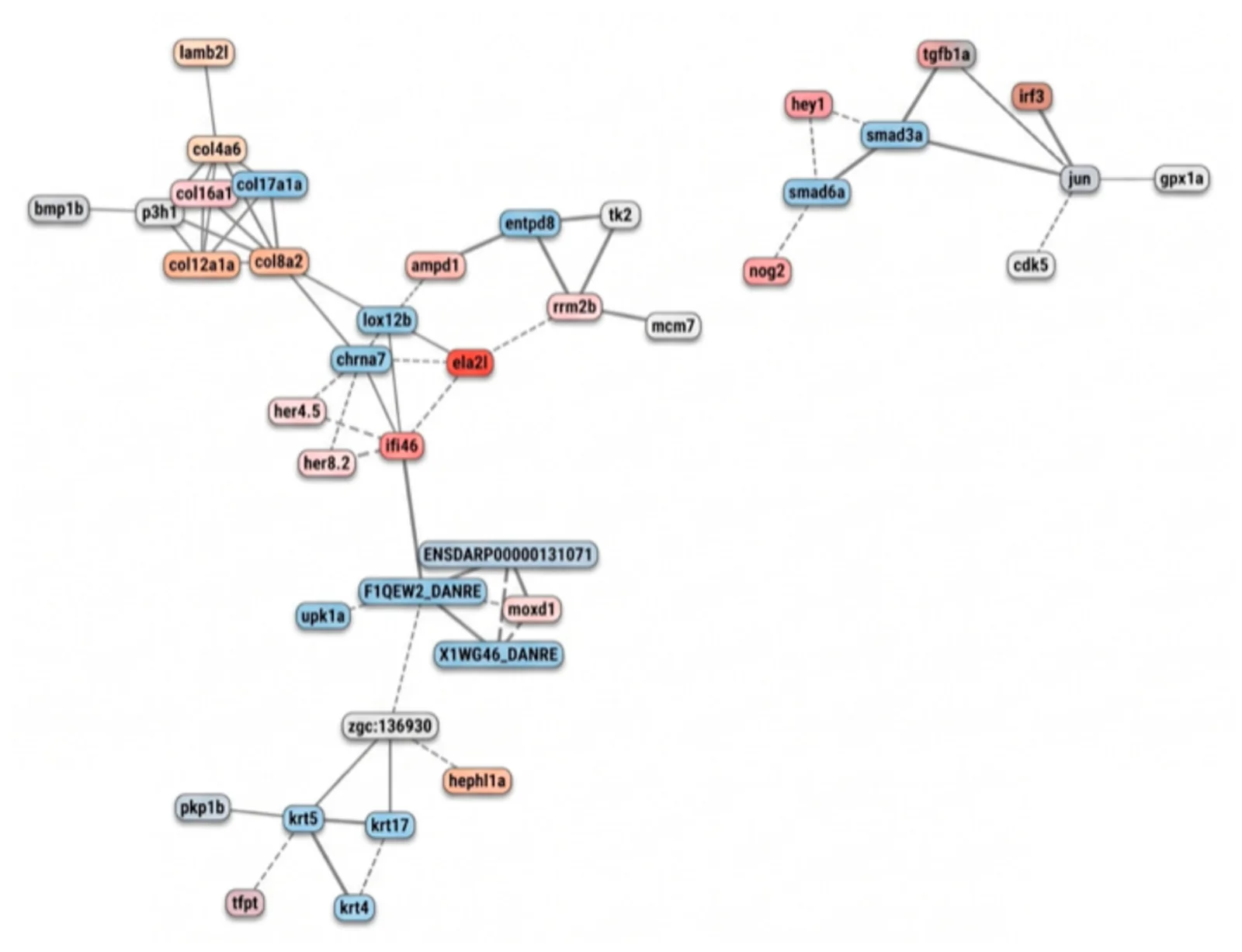
Clusters of the associative gene network of the differentially expressed genes. Built on the 256 DEGs with a STRING identifier (combined-score threshold 0.400, no interactor proteins); shown are the 40 nodes of the six MCL clusters and the edges linking them. Node fill encodes the log_2_ fold change, from blue (reduced) through white to red (elevated); edge width is the STRING combined score. Solid edges indicate experimental, curated-database or coexpression support; dashed edges indicate literature co-mention alone. MCL 1 (TGF-β signalling) forms the separate component on the right; the remaining five clusters lie in the left-hand component, with membership listed in Table S21. Node labels are reproduced as STRING v12.0 supplies them, so that the figure can be matched against the database it was built from; two of them are ZFIN symbols that have since been superseded and appear in the text and in Table 3 under their current form, *chrna7* as *chrna7a* and *ifi46* as *ifi27.4*. The four labels that are protein identifiers or clone names (ENSDARP00000131071, F1QEW2_DANRE, X1WG46_DANRE, *zgc:136930*) have no official symbol and are resolved in Table S9. Layout: Cytoscape 3.10.3, Prefuse Force Directed.

MCL produced six clusters of at least five nodes, all robust to degree-preserving permutation (*p* < 0.001; Table S7); both MCODE clusters of this size coincide with MCL clusters. Composition, edge provenance and the leading enrichment term are given in Table 3 and the structure in Figure 1.

Of the 137 edges, 17 (12.4%) connect paralogues by Ensembl Compara. Robustness checks (Table S10) split the clusters into two groups. MCL 1, MCL 2 and MCL 5 retain a connected core after removing literature-only edges and after removing the highest-degree node; MCL 2 contains no literature-only edges at all. MCL 3 shrinks to four nodes when paralogue groups are collapsed and largely reflects the keratin family. MCL 4 and MCL 6 lose many edges without external support and have no significant enrichment; they are presented as hypotheses.

Across all 275 DEGs, the single significant term is KEGG:04382, cornified envelope formation (*p* = 1.07 × 10^−3^; nine genes, eight of them down-regulated—*col17a1a*, *krt17*, *icn2*, *cldnb*, *col17a1b*, *pkp1b*, *cldna* and *icn*—and one up-regulated, the type IV collagen *col4a6*. Significant terms appear exactly in the clusters that passed the robustness checks; edge provenance, topology and annotation are independent criteria, so their agreement strengthens the inference.

### 2.10. Cross-Study Comparison of Glucocorticoid-Related Gene Expression

The GR-dependent programme is coordinately shifted: the GR-induced set towards down-regulation and the GR-repressed set towards up-regulation, both at FDR < 10^−3^, with mirror symmetry across more than two thousand genes each. At the level of single genes, the clearest change is the reduction in ddit4 (−0.76; *padj* = 3.7 × 10^−5^); other known targets change coordinately without individual significance (Table S11). The sets include 70 of the 275 DEGs (Figure S8).

The 14-day profile does not agree with the 5-week one [26]: of 169 shared genes, direction agrees for 70 (41%; *p* = 0.031), and effect sizes correlate negatively (ρ = −0.25; *p* = 9 × 10^−4^). Three of the four genes significant in both studies are interferon-inducible and change in opposite directions (Table S12). Enrichment by the Aβ42 signature is weak, so the response to CUS does not reduce to shared effectors of neuroinflammation. Of nine DEGs between fluoxetine and CUS in Demin et al. [26], four change opposite to the stress effect, consistent with partial normalisation; the set is too small for enrichment analysis.

### 2.11. Tissue Composition of the Libraries

Several of the largest effects in the DEG list belong to genes with epithelial rather than neural assignment in zebrafish [27,28,29]. Because the dissected region comprised the telencephalon together with the olfactory bulb [1], carry-over of adjacent epithelium is a plausible source of such transcripts, and we tested it directly rather than treating it as a caveat.

We formed composite content scores as the mean log_2_ expression of marker panels for neurons, radial glia, oligodendrocytes, microglia, epidermis, olfactory epithelium, erythrocytes, endothelium, pineal gland, skeletal muscle, cartilage and eye, excluding from every panel any gene that is itself a DEG, so that the score cannot be circular (Table S17). Radial glia are the principal macroglial cell type of the teleost brain and serve there the functions carried by astrocytes in mammals [30,31,32], so the panel is informative about the glial compartment as a whole. Neuronal content is indistinguishable between the groups (−0.01 log_2_ units), as is the content of oligodendrocytes, microglia, endothelium and erythrocytes. Epidermal content is 1.86 log_2_ units lower in the stress libraries, about 3.6-fold and the largest difference among the twelve panels, and radial-glial content is 0.26 log_2_ units higher (Figure S9).

The weight this comparison can carry is limited, and we state the limits here. The nominal *p*-values are 0.047 for epidermis and 0.040 for radial glia; across the twelve panels both become 0.28 after Benjamini–Hochberg correction, and under leave-one-library-out the epidermal comparison ranges from 0.037 to 0.114, so only one of the six reduced datasets keeps it below 0.05, although removing any single marker leaves it between 0.042 and 0.061 (Tables S26 and S27a,b). At three pools per group, these comparisons are descriptive rather than confirmatory. The case for treating epithelial transcripts as suspect rests not on them but on the convergence of three different kinds of evidence: the zebrafish annotation of the genes concerned, their per-library pattern, and the dissection, which placed olfactory and surface epithelium immediately adjacent to the sampled tissue.

Attribution of individual genes to a contaminating tissue is not possible at three pools per group, and we report this because it bounds what such a dataset can support. Correlating each DEG with the epidermal score after removing the group means flags 6.5% of DEGs at |r| > 0.8, against 5.3% of expression-matched non-DEGs (Kolmogorov–Smirnov *p* = 0.59), and a decoy panel of nine randomly chosen genes flags as many DEGs as the epidermal panel does. We therefore reassigned genes on published zebrafish evidence [27,28,29], together with a second criterion applied to the counts themselves: a gene absent from every library of one group and present in every library of the other. Twenty-eight of the 275 DEGs meet one of these criteria (14 epidermal or mucosal, six assigned to another non-neural organ, eight all-or-none across pools; Table S18). The evidence behind these assignments is of unequal strength, and Table S18b now grades it against the zebrafish expression record, which was consulted for every gene. Three genes—*krt4*, *muc5.2* and *nphs2*—have expression recorded in the attributed non-neural tissue in adult fish and none in neural tissue. Eleven have zebrafish evidence in that tissue at an embryonic or larval stage, or at an unresolved stage. For four—*cdh1*, *cldna*, *cldnb* and *opn1mw2*—the record also includes expression in adult brain or nervous system, so it does not discriminate the origin of the transcript. For two, *st14b* and *tmprss15*, there is no zebrafish expression annotation at all, and the assignment rests on orthology to the mammalian gene. Table S19d repeats the exclusion for these nested subsets. The glucocorticoid tests are unaffected at every level (*p* between 0.0010 and 0.0016). The epithelial terms behave as the grading predicts: three of the sixteen fall with the three adult-supported genes, and the intermediate-filament, keratinocyte-differentiation and basement-membrane terms fall when the eleven genes of the second group are set aside as well. Neither outcome is a claim about origin. The genes are set aside not because the record shows them to be contaminants but because it cannot show where the transcripts came from—least of all for the four whose record places them in skin and adult brain alike—and a term that rests on transcripts of undecidable origin is not read here in either direction. The only claims the exclusion removes are our own; it adds none.

Removing them does not change the glucocorticoid result (GR-induced *p* = 0.0015 → 0.0013; GR-repressed *p* = 0.0015 → 0.0010) and does not change MCL 1, which loses no member. It does change the epithelial part of the interpretation: enriched GO terms fall from 16 to 5, the intermediate-filament, keratinocyte-differentiation and basement-membrane terms are lost, MCL 3 falls from seven genes to three, and seven of the nine member genes of KEGG:04382 (cornified envelope formation) are among those set aside, so that term is no longer enriched. The terms that survive are regulation of neurogenesis, of nervous-system development, of cell development, of cell differentiation, and collagen trimer (Table S20). The weight of the first of these statements should not be overdrawn. Of the two glucocorticoid sets, 1 of 2443 and 3 of 1951 members appear on the exclusion list, and none of the four is an epidermal transcript: three are unannotated loci with an all-or-none count pattern, and the fourth is the kidney gene *nphs2*. There is accordingly almost nothing in these sets for the removal to remove, and the outcome carries little information about whether tissue composition affects them.

Finally, we asked whether the glucocorticoid result travels with composition rather than with treatment. A model containing the group factor and the epidermal score as measured cannot answer this, because the two are collinear (*r* = −0.82) and the model loses power: random covariates of equal collinearity reduce the number of DEGs to a median of 84 and remove the GR signal in 14 of 31 cases (Table S19c). Epidermal content also varies within each group, however, by 1.74 log_2_ units among the control pools and 1.28 among the stress pools, and centring it within groups gives a covariate orthogonal to the group factor by construction. In a model containing both, the GR sets move with the group (induced down, *p* = 0.0021; repressed up, *p* = 0.0016; 246 genes at FDR < 0.05) and not with composition (induced *p* = 0.11; repressed *p* = 0.41; 16 genes at FDR < 0.05), and the trend along the composition axis runs opposite in sign to the stress effect (Table S28). Consistently with this, one of the 2443 tested GR-induced genes and three of the 1951 GR-repressed genes appear on the exclusion list. The limit of this test should be stated plainly: because the covariate is orthogonal to the group factor by construction, it tests only the within-group component of epidermal content. The difference between the group means in epidermal content remains collinear with the treatment and cannot be separated from it in these data; what the test shows is that variation in composition large enough to span 1.3–1.7 log_2_ units within a group does not move the glucocorticoid sets, and that where it does move them the direction is opposite. Section 3.9 states the residual confound. Two further points belong here. None of the fifteen epidermal marker genes is a member of either glucocorticoid set, against 1.4 and 1.1 expected, and across the twelve panels no panel is over-represented in either set once the twenty-six tests are corrected (smallest adjusted *p* = 0.67; Table S37), so a change in epidermal content has no direct representation in the two sets—though transcripts shared between skin and brain would not be caught by a marker panel. Neither that observation nor the orthogonal-covariate model excludes tissue composition as an alternative explanation of the group-level result, and neither is presented as doing so.

### 2.12. Genomic Distribution of the Differentially Expressed Genes

The DEG list is not randomly distributed along the genome. Among the 275 genes, there are 48 pairs separated by less than 200 kb on the same chromosome, of which 37 change in the same direction. Against 2000 random gene sets matched to the DEG list decile by decile on mean normalised expression, the expectations are 15.2 and 7.8 pairs, respectively (95% ranges 8–23 and 3–14; *p* < 0.0005 in both cases; Table S29). Matching on expression leaves the expectation unchanged, so the clustering is not a by-product of the expression levels at which differentially expressed genes are found. The largest blocks consist of loci with large fold changes and zero counts in one group. On chromosome 8, three consecutive DEGs span 17.5 kb—*tnfrsf14* and two loci unnamed in Ensembl 112 (ENSDARG00000089901 and ENSDARG00000093392, named si:ch211-261n11.8 and CT583651.1 in the annotation used by [1])—each raised by about four log_2_ units, which is roughly sixteenfold (Figure S10). We state the inference at the strength the data support. Positional clustering of this kind has several possible sources: shared haplotypes, copy-number differences, local regulatory domains, shared chromatin states, and the tandem organisation of gene families all produce it, and RNA-seq from one time point cannot separate them. It is therefore a reason to test for linked genetic variants or other local effects—genotyping of the pooled animals would settle it—and not by itself evidence of structural variation. The her4 cluster on chromosome 23 is the informative exception: *her4.3* and *her4.5* lie 9.9 kb apart and change together, but they are tandem paralogues and known Notch targets, so co-regulation is expected there. The effect is also not a blanket positional one: *her4.4* lies between them and does not change (log_2_FC +0.09), which is what co-regulation of specific paralogues looks like and not what a shared haplotype across the array would look like. A point of nomenclature belongs with this pair: in the Ensembl 89 annotation used by [1], the second gene carried the symbol *her4.2*, which in Ensembl 112 denotes a different locus (ENSDARG00000094426) that is not differentially expressed here. It is one of four DEG symbols reassigned to another gene between the two annotations (Table S34), and 70 of the 275 DEGs carry a different symbol in the two releases. The ambiguity is present in the source table as well: in the supplementary table of [1] the symbol *her4.2* is attached to two different Ensembl identifiers, one of which is reported there as differentially expressed (log_2_FC +1.41, padj 0.020) and the other not (+0.21, padj ≈ 1); 289 symbols in that table are attached to more than one identifier, covering 762 genes, and 10 of our 275 DEGs carry such a symbol (Table S34c). Comparison between the two analyses was therefore made on stable identifiers throughout, and we give identifiers alongside symbols wherever a specific locus matters. Genomic adjacency is a flag for inspection, not a criterion for exclusion, and we used it only together with the all-or-none count pattern. Taking the most conservative course and setting aside every gene flagged by either class—the 28 reassigned in Section 2.11 together with the same-direction members of the genomic blocks, 85 genes in all, leaving 190—the receptor-level shift is unchanged in direction and slightly stronger (induced down, *p* = 0.0008; repressed up, *p* = 0.0014; Table S33). The flags therefore bear on the interpretation of individual genes, not on the receptor-level result.

The same consideration applies to enrichment statements, which treat their member genes as independent observations. Of the two protein domains reported as enriched among up-regulated genes in the original publication [1], the three genes carrying the interferon-induced 6–16 (IFI6/IFI27-like) domain—*ifi27.1*, *ifi27.2* and *ifi27.4* (*zgc:123068*, *zgc:152791* and *si:dkey-188i13.7* in [1])—lie within 17.4 kb of one another on chromosome 13 and form a single tandem cluster; their counts rise in two of the three stress pools rather than in all three. That enrichment therefore rests on one locus counted three times. The four Orange-domain genes lie on four different chromosomes—*her6* on 6, *her8.2* on 15, *hey1* on 19 and *her4.5* on 23—so they are independent. The present reanalysis reproduces that signal: after the exclusion of Section 2.11, the surviving enriched terms are regulation of neurogenesis and of nervous-system development, carried by seven genes across six chromosomes with a minimum separation of 5.5 Mb. We applied the test to our own list as well: two terms, peripheral nervous system neuron development and differentiation, rested on the adjacent *her4.3*/*her4.5* pair and are not reported (Table S25).

## 3. Discussion

### 3.1. Recovery of Replicates

The loss of replicate structure on submission turned out to be reversible, and recovering it is simpler than it first appears. The key lies in the order in which the archive loader processes input files: it numbers the aligned reads of each file consecutively and places the unaligned ones at the end of the corresponding block, which leaves a discernible structure in the merged run. The procedure requires no contact with the authors of the original study and does not depend on read identifiers having survived. Two of the six checks give exact rather than approximate agreement: the library sizes sum to the number of spots in the run with zero discrepancy, and the read counts of the extracted libraries match the expected values to the single read. Records with a similar structure are, judging by the submission rules, unlikely to be rare.

### 3.2. Relation to the Original Publication

The reanalysis does not contradict the published result but refines it. A correlation of effect-size estimates of ρ = 0.754 and directional agreement for 98.5% of previously reported DEGs mean that the list of Huang et al. [1] contains no systematic error. The number of detectable DEGs nevertheless rises from 155 to 275, with 174 genes that previously did not reach the significance threshold. The cause is measurable: the deposited alignment was performed without splicing, so reads spanning exon–exon junctions were not counted at all. There are 13.1–16.6 million of them per library, which accounts both for the 17-percentage-point difference in aligned fraction and for the difference in statistical power.

The most significant of the additionally identified genes is *prdm2a* (log_2_FC = +5.12; *padj* = 4.7 × 10^−23^), a histone methyltransferase of the PRDM family, a family with established roles in development and in the maintenance of progenitor states [33]. A change in expression of the histone deacetylase HDAC4 under prolonged stress exposure has already been shown in this species [3], so the involvement of chromatin modifiers in the stress response of *D. rerio* is established independently; the present data indicate that such changes are already detectable at day 14 and extend to methyltransferases. Chromatin regulation is a recognised component of stress-related phenotypes [34,35], and H3K9 methylation in particular has been linked to stress exposure in rodent hippocampus [36]. Other notable additions are *smad3a* (−2.36), which belongs to the cluster with the strongest enrichment, and a group of amino acid and neurotransmitter transporters (*slc6a17*, *slc38a3a*). Of the 56 DEGs represented in the GRCz10 annotation by clone identifiers, 45 are detectable here and 35 are significant. This group contains the rapidly evolving teleost-specific families, including the interferon-inducible ones; their representation in results depends on the annotation version more strongly than that of conserved genes.

An independent check is available in the original study itself. Its Supplement 3 lists primer pairs for 19 genes validated by RT-qPCR against *ef1a*, a panel chosen by the original authors and not by us. We resolved each entry to a stable identifier through the supplementary table of [1] rather than by symbol (Table S35); 18 resolved to a single gene with an estimate in both analyses, while one, listed there as *her4.2*, cannot be resolved because that symbol is attached to two identifiers in the table of [1]. For the resolved 18, the two analyses agree closely (Spearman ρ = 0.96, Pearson *r* = 0.98, against ρ = 0.754 across the whole published list). Fifteen have an absolute log_2_ fold change above 0.3 in at least one analysis and all 15 agree in direction; the remaining three (*ache*, *npy*, *nr3c1*) are near zero in both, so the sign carries no information. We are more conservative on significance: 11 reach FDR < 0.05 here against 14 in the published list, the three differences being *igfbp2a* (*p* = 0.70 here, 0.005 there), *nub1* (0.052, 0.00028) and *her6* (0.35, 0.010); no gene of the panel becomes significant here that was not significant there. In the other direction, *chrna7a* is more firmly called here than in the original analysis (padj 3.2 × 10^−4^ against 2.3 × 10^−2^). Only one member of the panel, *ela2l*, is on the exclusion list of Section 2.11. Two features of the panel bear on the interpretation offered here. First, the receptor message does not fall. In the telencephalon, *nr3c1* is essentially unchanged (log_2_FC +0.04 here, +0.01 in the table of [1]), as are *hsd11b2*, *ache* and *npy*. The whole-brain RT-qPCR of [1], on a separate cohort of five animals per group, gives the same directions with larger amplitude and reports two of them as significant: *nr3c1* raised (*p* = 0.031) and *hsd11b2* reduced (*p* = 0.006), with *ache* and *npy* unchanged (*p* = 0.13 and *p* = 0.99); the female cohort of that study gives the same three directions, with *ache* significant there as well [1]. The two measurements agree in sign and differ in magnitude, which is unsurprising when tissue, method and cohort all differ; neither shows the receptor message falling, and a longer protocol in the same species raises brain GR expression as well [3]. The programme those receptors drive nevertheless shifts in the attenuating direction, so the attenuation is not explained by a loss of receptor message—if anything, the message moves the other way (H1). Second, the genes of the panel that do change are distributed across collagen maturation (*loxl2b*, *col8a2*), redox (*gpx1a*), cholinergic signalling (*chrna7a*), cell-cycle and immediate-early regulation (*cdk5*, *jun*) and Notch signalling (*hey1*, *her8.2*), which is the diffuse pattern a receptor-level effect produces and not a single-pathway response.

The genes themselves behave as the measurement account predicts. Of the 174, 163 have an effect-size estimate in the original table, and 148 (90.8%) agree with ours in direction (Spearman ρ = 0.87), against 79.1% for randomly expressed genes significant in neither analysis (binomial *p* = 5.2e−05). Twenty-four had an adjusted *p*-value below 0.10 originally and 61 below 0.25, with a median of 0.54 against 1.00 for randomly expressed genes: they stood high in the original ordering without crossing its threshold. They are also no more likely than the previously published genes to carry an artefact flag from Section 2.11 (10.9% against 8.9%; Fisher *p* = 0.68); Table S31.

The two statistical components can be separated, and neither accounts for the result alone. Recomputed on the common gene set under a normal approximation, the published estimates with the published standard errors give 140 genes, and ours with ours give 262; the published estimates with our standard errors give 481, and our estimates with the published standard errors give 238. The gain in precision alone would yield far more differentially expressed genes than we report, and is partly offset because our effect-size estimates are systematically smaller: the median absolute log_2_ fold change across common genes is 0.125 here against 0.149 in the original. These counterfactuals combine quantities from two pipelines under a normal approximation and are given as a decomposition, not as predictions. The contributions of the aligner, the assembly and the annotation cannot be apportioned among themselves, because all three changed together; that would require a controlled series varying one factor at a time, which we did not run.

The original analysis was not performed without replicates: three pooled samples per group were prepared and analysed as three replicates, and the published table reports a standard error and an adjusted *p*-value for every gene. What the archive lost was the replicate structure, not the replication itself, so the recovery described in Section 2.1 is what makes an independent reanalysis possible rather than a source of additional yield. The increase from 155 to 275 is attributable to quantification. Of the 174 genes significant here but not in the published list, 11 are absent from the original table, an effect of the annotation; 4 were filtered out before testing; and 159 were tested and did not reach FDR < 0.05. Across the 19,175 genes tested in both analyses, the median baseMean rises 1.91-fold and the median standard error of the log_2_ fold change falls 0.78-fold, being smaller here for 85% of genes (Table S32).

### 3.3. Matrix Changes and Their Boundaries

Matrix changes proved isoform-specific. Of the six type IV collagen genes and four laminin beta chains, one each is changed—*col4a6* and *lamb2l*—and of the two paralogues of the lysyl oxidase-like enzyme, only *loxl2b*. The teleost genome underwent an additional round of duplication [37,38], and many genes are represented by paralogues with partly diverged functions; selective change in one paralogue while the other stays put indicates specificity of regulation rather than a general shift in matrix gene expression. For cross-species comparison and for planning experimental validation, it matters which paralogue is considered: in humans, the corresponding functions are often carried by a single gene.

Within the collagen maturation system, the directions diverge: prolyl 3-hydroxylase *p3h1* and lysyl oxidase *loxl2b* are reduced while procollagen C-proteinase *bmp1b* is elevated. Prolyl 3-hydroxylation modifies a single residue of the triple helix and, unlike 4-hydroxylation, does not govern its thermal stability; cross-linking of mature fibres is initiated by lysyl oxidase [39], whereas BMP1 acts as the procollagen C-proteinase [40]. Taken together, these shifts point to a change in the composition and degree of cross-linking of the matrix rather than in the volume of its synthesis. These data do not speak to the blood–brain barrier, and one observation shows why. The claudins that change here, *cldna* and *cldnb*, are not the claudins of the vascular barrier: in *D. rerio* that function belongs to *cldn5a* [41], whose paralogue CLDN5 serves the same role in mammals [42]. Neither *cldn5a* (+0.23) nor *cldn5b* (+0.33) changes in these data. Type XVII collagen anchors epithelium to the basement membrane [43], but *col17a1a* is reassigned in Section 2.11 as a transcript of probable epidermal origin, and the basement-membrane term does not survive that reassignment, so these data do not support a statement about the vascular basement membrane or about barrier permeability. The increase in barrier permeability reported in the same species under longer exposure [3] is a question for direct measurement, not a comparison these data can make.

The single significant enrichment term across the full DEG list needs a comment. The cornified envelope is a structure of the keratinising epidermis of amniotes and is absent in teleosts; the annotation reaches *D. rerio* by orthology. Seven of its nine member genes—the claudins *cldna* and *cldnb*, the plakophilin *pkp1b*, the keratin *krt17*, the type XVII collagen *col17a1a*, and the ictacalcins icn and icn2, calcium-binding proteins of the S100 family reported in the epidermis [28]—are of probable epidermal origin by the criteria of Section 2.11, and the term is not enriched once they are set aside. The two remaining members are of a different character: *col17a1b* is expressed in the nervous system of *D. rerio* unlike its skin paralogue and is retained, and *col4a6* is a basement-membrane collagen that rises rather than falls. The term is therefore not a statement about intercellular contacts or about the barrier function of epithelium in brain tissue. Whether the seven transcripts come from epidermis carried over in dissection or are expressed in the telencephalon itself is not settled by expression data: the assignment rests on the tissue specificity of the markers and on all-or-none count patterns, which is reason to set the genes aside pending a direct test rather than a demonstration of carry-over. In situ hybridisation or immunostaining on sectioned brain would settle it.

### 3.4. Different Levels of Regulation

Comparing levels of regulation yields a substantive distinction. Of the antioxidant defence system, only *gpx1a* is changed, while superoxide dismutases, catalase, peroxiredoxins, the thioredoxin system and the transcription factor *nfe2l2a* remain at control level; the monoamine oxidase transcript is likewise unchanged. Yet stress models do show changes in monoamine oxidase activity, lipid peroxidation and mitochondrial function [20,21,23], and in *D. rerio* under CUS, changes in mitochondrial proteins of the brain proteome have been described [18]. Transcriptomic data belong to a different level of regulation, and the discrepancy between levels is informative in itself. At day 14, the oxidative and monoaminergic components of the stress response, if present, are not reflected in the changes in expression of the corresponding genes. Monoamine oxidase activity is governed primarily by post-translational mechanisms and substrate availability.

The changes in cluster MCL 5 admit a specific interpretation. Thymidine kinase *tk2* and ribonucleotide reductase subunit *rrm2b* supply deoxyribonucleotides for replication of mitochondrial DNA: mutations in both genes cause mitochondrial DNA depletion syndrome [44], *rrm2b* is required to maintain the mitochondrial deoxyribonucleotide pool specifically in quiescent cells [45], and it is induced by DNA damage and oxidative stress [46]. AMP deaminase *ampd1*, the most strongly elevated gene of the cluster (+3.73), catabolises adenine nucleotides and is activated when the energy charge falls. Two qualifications belong with this reading: *ampd1* is assigned in Section 2.11 to the striated-muscle category and is therefore excluded from the quantitative claim, and *entpd8* shows a composition-linked per-library pattern. The interpretation below rests on *tk2* and *rrm2b*. The extracellular arm of the purinergic system is practically untouched. What we see, then, concerns intracellular nucleotide and energy metabolism rather than purinergic signalling, and it agrees with data on mitochondrial dysfunction in stress-associated states [18,20].

### 3.5. Network Clusters

Of the six clusters robust to the permutation test, four show significant functional enrichment and retain a connected core when requirements on edge provenance are tightened. The cluster with the strongest enrichment, MCL 1, brings together components of TGF-β signalling (*tgfb1a*, *smad3a*, *smad6a*), Notch-dependent regulation (*hey1*, *nog2*), the AP-1 factor (*jun*), an interferon-regulatory factor (*irf3*), an antioxidant enzyme (*gpx1a*) and a cyclin-dependent kinase (*cdk5*). Four edges of this cluster are supported by the experimental source alone and are therefore not explained by transfer of curated annotation.

Cluster MCL 2 is the only one containing no edges without external support and is entirely covered by curated databases. Cluster MCL 3 brings together keratins and a plakophilin but shrinks to four nodes after paralogue groups are collapsed, so it substantially reflects family membership. Clusters MCL 4 and MCL 6 are presented as hypotheses: in MCL 4, removal of edges without external support leaves four connections, including the only non-literature link, *chrna7a* with *ifi27.4*, and the cluster has no significant enrichment. The α7 receptor participates in the cholinergic anti-inflammatory pathway and suppresses cytokine release by macrophages [47,48], a line of work that continues to develop [49], which makes its link to an interferon-inducible gene biologically plausible—though in the present data that link rests on a single edge. The differential expression of *chrna7a* is a measurement and is confirmed here with greater significance than before (*padj* = 3.2 × 10^−4^), whereas any claim about its central position in the network does not have that status and is not made.

Connectivity of the query list exceeds chance only under control for study coverage, and the direction of the correction is counter-intuitive. Without degree matching, the test would detect no effect, because the DEGs turned out to be shifted towards less studied genes than the transcriptome background. The bias of interactome data towards well-studied proteins is documented as a source of systematic error [50,51], but it is almost always discussed in connection with false-positive conclusions. Our case shows the other side: in species with incomplete annotation, control for study coverage also guards against false negatives.

### 3.6. Glucocorticoid-Dependent Transcription

The most substantive biological result is the coordinated shift in the GR-dependent transcriptional programme. The inference rests on two independent gene sets obtained experimentally in a receptor knockout model [52] and does not depend on selecting individual targets from the literature. The mirror symmetry of the result makes an explanation through a general shift in the distribution unlikely.

The behaviour of *ddit4* deserves separate comment. In rodents, chronic unpredictable stress raises REDD1 protein levels in the prefrontal cortex, and the increase is mediated by glucocorticoid release; deletion of the gene renders animals resistant to stress-induced synapse loss [12]. In the present data, *ddit4*—the most significant of the well-characterised receptor targets—is reduced (−0.76; *padj* = 3.7 × 10^−5^). The direction is opposite to that described for the active phase of stress exposure in rodents and consistent with attenuated glucocorticoid signalling: if induction of *ddit4* requires an active receptor, its reduction is expected when transcriptional output falls. The difference may also reflect the sampling time—24 h after the last stressor rather than during exposure—as well as species and brain region.

The result is consistent with the sampling conditions. Tissue was collected 24 h after the last stressor, when cortisol did not differ from control according to the original publication. Reduced transcriptional output of the receptor at this point is also consistent with the fall in cortisol under prolonged CUS [3] and with observations of low cortisol in PTSD comorbid with depression. The present work establishes the fact of the shift, not its mechanism: the data do not distinguish reduced ligand availability from altered receptor sensitivity or a rearranged cofactor environment.

The glucocorticoid programme and the substantive core of cluster MCL 1 barely overlap: of the nine genes in the cluster, only *jun* belongs to the GR-dependent sets. These are two independent results, and the available data give no grounds for combining them into a single scheme.

Two kinds of artefacts have to be kept apart here. One acts on individual genes, where a transcript of non-neural origin enters the list on its own account; the other acts on the sample, where a difference in the proportions of cell types moves many genes at once. The checks of Section 2.11 address the first: the shift survives removal of the reassigned genes, and it is carried by the group factor rather than by an orthogonal composition term. They do not address the second, and the slight intersection between the exclusion list and the two gene sets—four genes of 4394—is the reason: there is almost nothing in these sets for such a removal to remove. Epidermal content differs between the groups and is collinear with the treatment, so the two cannot be separated in six libraries, and a difference in tissue composition remains an alternative explanation of the group-level result which the analyses reported here do not exclude. These use the same six libraries and the same two gene sets, so they establish robustness rather than independent confirmation, which would require new animals. One further question can be asked within the data: how specific is the mirror pattern to these gene sets? Drawing 2000 random pairs of gene sets of the same sizes from the same ranked statistics, 0 produced both members significant with opposite directions, giving an empirical bound of *p* < 0.0005 (Table S30). A difference between the two groups of fish arising from housing, handling or batch would therefore have to be aligned with the glucocorticoid axis specifically; a general difference does not produce this pattern. That narrows the space of alternative explanations without eliminating it, since a nuisance factor acting through glucocorticoid physiology would be indistinguishable from the treatment in these data.

### 3.7. Divergence of Profiles at Different Exposure Durations

Comparison with the 5-week protocol [26] gave a result that is negative in sign: the direction agrees for only 41% of shared genes, and the correlation of effect-size estimates is negative. Three of the four genes significant in both studies are interferon-inducible and change in opposite directions. This observation is consistent with the reported switch from a pro-inflammatory to an anti-inflammatory profile as exposure lengthens [3], but the studies compared differ not only in duration, but also in brain region (telencephalon versus whole brain) and in laboratory and protocol details. We therefore describe the finding as a divergence of profiles rather than temporal dynamics; the latter can be established only by comparison within a single design.

A practical limitation follows. DEG sets obtained at different durations of stress exposure in one species cannot serve as mutual validation. This constrains the common practice of confirming the results of one study with the DEG list of another without regard for design comparability.

Weak enrichment by the Aβ42 response signature shows that the transcriptomic response to CUS does not reduce to shared effector components of neuroinflammation. This limits the interpretation of overlaps between stress and neurodegenerative models: agreement of functional categories reflects shared terminal steps rather than the specificity of the primary stress response.

### 3.8. Hypotheses Arising from These Results

Each hypothesis below rests on the present results and on published observations in the same species, and each specifies a test. They are conjectures, not established results.

**H1.**  *The attenuation lies downstream of receptor messaging and of ligand availability. Three upstream variables point away from the simplest explanations. Trunk cortisol is at control level at the moment of sampling, 24 h after the last stressor, although it was raised 15 min after it [1]. Receptor message does not fall: nr3c1 is unchanged in the telencephalon in both analyses and is reported raised in whole brain by the RT-qPCR of [1] (p = 0.031), and a longer protocol in the same species raises it as well [3]. The enzyme that inactivates cortisol locally, hsd11b2, is unchanged here and reduced in that same whole-brain assay (p = 0.006), which would leave more ligand available rather than less. The attenuation therefore runs against the direction of both upstream variables rather than following them. The glucocorticoid-dependent programme nevertheless shifts. The attenuation should therefore sit at receptor protein abundance, nuclear translocation, cofactor availability or chromatin accessibility at receptor-bound sites rather than in the amount of ligand or of receptor message. Test: GR immunostaining with nuclear and cytoplasmic fractionation of dissected telencephalon, and chromatin immunoprecipitation for GR at the promoters of fkbp5, tsc22d3, klf9 and ddit4, with those four transcripts and cortisol measured in the same animals and sampling extended beyond two weeks. A negative result for translocation would point to cofactor or chromatin-level regulation and would distinguish this state from the receptor up-regulation reported at five weeks in the same species [3]. This hypothesis presupposes that the shift is a property of the tissue rather than of its composition, which the present data cannot establish (Section 3.9).*

**H2.**  *TGF-β signalling drives paralogue-specific remodelling of collagen. MCL 1 (tgfb1a ↑, smad3a ↓, smad6a ↓) and MCL 2 change together, while duplicate paralogues keep baseline expression. The basement-membrane term does not survive the exclusion of Section 2.11, and the surviving evidence is the collagen-trimer term, so the prediction concerns collagen maturation rather than the vascular basement membrane. Test: phospho-SMAD3 and immunostaining for the collagens of MCL 1 and MCL 2.*

**H3.**  *A subset of animals mounts an interferon response under this protocol. The present data raise this possibility rather than support it: the interferon-inducible transcripts concerned form one tandem cluster within 17.4 kb on chromosome 13, so they are a single observation, and they rise in two of the three stress pools rather than in all three. Test: per-animal rather than pooled sampling, with genotyping of the chromosome 13 locus, so that an induced response can be distinguished from a copy-number or haplotype difference.*

**H4.**  *Chromatin modifiers, including H3K9 methyltransferases, join the early stress response. prdm2a, an H3K9 methyltransferase, is the most strongly elevated gene in the list and is not flagged by either artefact class. Test: in situ hybridisation, knockdown, and H3K9me2 immunostaining in the telencephalon.*

**H5.**  *Early oxidative changes occur without transcriptional activation of antioxidant genes. Only gpx1a changes and mao is unchanged, so regulation is likely post-transcriptional. Test: enzyme activities and oxidative-damage markers rather than transcripts.*

**H6.**  *Glial–neuronal glutamine transfer changes while glutamate reuptake is intact. slc38a3a falls and slc6a17 rises, with glutamine synthetases and the glial glutamate transporters unchanged. Test: double labelling with glutamine synthetase and a neuronal marker, and measurement of glutamine flux.*

**H7.**  *Changes in nucleotide metabolism reflect mitochondrial stress rather than purinergic signalling. tk2 and rrm2b change while the purinergic receptors do not. ampd1 and entpd8 are excluded from this prediction: the first is reassigned in Section 2.11 as a striated-muscle transcript and the second has a composition-linked per-library pattern. Test: mitochondrial DNA copy number and adenine-nucleotide pools.*

These processes may reinforce one another—altered collagen maturation and a shift in glial–neuronal glutamine transfer are both compatible with the same glucocorticoid signal—but the present data constrain the direction of such coupling only weakly. No hypothesis on transcriptional weakening of an epithelial or vascular barrier is advanced here: the claudin, plakophilin and keratin transcripts on which such a prediction would rest are of probable epidermal origin by the criteria of Section 2.11.

### 3.9. Limitations

- Design of the source study: housing and the unit of treatment. The published report states that four telencephala were pooled per sample and three samples prepared per group from a separate cohort of 24 males, and that animals were housed in 3 L tanks at a density of no more than 5 fish/L, which permits up to fifteen fish per tank; twelve fish per condition therefore fit within a single tank. The number of tanks per condition and the assignment of fish to tanks are not reported, and the deposited record adds nothing. The six stressors differ in the level at which they were delivered, and the published description does not resolve all of them. Two were delivered to individual fish: social isolation, in a separate 250 mL vessel, and restraint, by mesh dividers within the home tank. Two were delivered to a group: overcrowding, by mesh dividers in the home tank, and tank change, by netting between two tanks. For the remaining two, shallow water and the object chase, the number of fish treated at one time is not stated and cannot be inferred (Table S36). The level of delivery does not by itself fix the experimental unit, however, which is set by the level at which the treatment was allocated. That level here is the housing group: the protocol states that control animals were not handled during the fourteen days, so the control and stressed fish of a cohort cannot have shared a tank. A stressor delivered to one fish at a time therefore does not create independent replication, because the fish receiving it were not independently assigned to receive it. The twelve fish of a condition are subsamples of one or more treated groups rather than independent replicates of the treatment. If each condition occupied a single tank, the effect of the treatment is not separable from the effect of the tank. We searched the article, its three supplementary files and the SRA record. Five things cannot be recovered from them: the number of tanks per condition, the assignment of fish to tanks, the vessel used for shallow water and whether fish were treated singly or together, the number of fish chased at one time during the object chase, and whether the stressed and control fish of a cohort stood on the same rack. We state this rather than settle it by assumption.Two features of the housing narrow the problem without removing it. The animals were kept in a rack with closed-system filtration, so the water is shared between tanks and the most obvious tank-level variables—water chemistry, temperature and dissolved gases—are common to all of them; what remains is the position of a tank on the rack, the social composition of its occupants and the handling that accompanies the tank-level stressors. One of the six stressors, moreover, consisted of netting fish between two tanks eight times, so a single fixed home tank is not an accurate description of the stressed group either. A related feature of the comparison belongs in the same place: the published protocol states that control animals were not handled during the fourteen days, so the contrast is between stressed and undisturbed fish rather than between stressed and sham-handled fish, and handling is part of what distinguishes the groups. This is usual in chronic unpredictable stress protocols and is not peculiar to this study, but it bears on how the resulting signature should be read. The confound is therefore narrower than the phrase ‘tank effect’ suggests, but it is not eliminated, and is not treated as eliminated here.This is a limitation of the data rather than of the analysis. It constrains what these libraries can establish; it applies in the same degree to the original analysis of the same animals, and no computational procedure applied after the fact can remove it. Separating the two effects requires randomisation within tanks, so that both conditions are represented in every tank; we note this for the design of prospective experiments rather than as a criticism of a study carried out for a different purpose.
- **What the recovery of the libraries establishes.** The six checks establish that the deposited runs are concatenations of contiguous source files and that we have found those boundaries. That each recovered block corresponds to one independent biological pool is an inference from the published description of the experiment, not a property we have demonstrated; correlation structure and dispersion are consistent with it but cannot distinguish independent pools from technical replicates of the same pool.
- **Region mismatch of the external gene sets.** The gene sets used to characterise glucocorticoid-dependent transcription were derived from whole brain, whereas the present contrast is telencephalon with olfactory bulb. Regional heterogeneity would dilute such a comparison and move it towards the null, so the bias is conservative with respect to detection; but the effect sizes reported here are not telencephalon-specific estimates and should not be compared quantitatively with whole-brain studies.
- **Composition and treatment cannot be separated.** Group and epidermal content are collinear in these libraries (*r* = −0.82), so a model containing both has little power and its result is uninformative in either direction. We therefore report the unadjusted analysis with the reassigned genes removed, together with the orthogonal-covariate analysis of Section 2.11, and we do not present an adjusted DEG list as a corrected one. The adjusted model is reported for completeness and not as a test of composition: random covariates of the same collinearity remove the glucocorticoid signal in 14 of 31 runs and reduce the number of differentially expressed genes to a median of 84, so its failure to reach significance (*p* = 0.358 and 0.107; Table S19b) is a property of the design and not a finding about glucocorticoids. The two readings—an attenuated receptor-dependent programme, and a difference in tissue composition aligned with the treatment—cannot be told apart in these data, and we do not claim to have excluded the second.
- **Computational evidence.** DEGs, clusters and enriched categories require experimental validation.
- **Within-pool variability.** Each library pools four males, so variation between individual fish within a pool is not observable, and the effective replication is three units per group.
- **Nature of the network.** STRING associations are not regulatory relations; clusters are not regulatory units, and the null models do not test regulatory function.
- **Evidence sources.** Textmining is biased against poorly studied teleost-specific genes; coexpression edges introduce partial circularity (excluding them removes 5 of 137 edges).
- **External gene sets.** They come from models of different aetiology, brain region, age and laboratory; conclusions concern profile agreement, not mechanism.
- **Annotation.** *D. rerio* annotation is incomplete and partly transferred by orthology; the detectable gene set depends on the assembly version.
- **Targeted systems.** Functional systems were assessed against predefined gene lists; unchanged transcripts do not exclude regulation of protein or activity.
- **Single time point.** Tissue was sampled 24 h after the last stressor; the data do not describe response dynamics.

## 4. Materials and Methods

### 4.1. Source Data

We used RNA-seq reads from the telencephalon with olfactory bulbs of sexually mature male *D. rerio* of the AB line, 11 months old (BioProject PRJNA422916; runs SRR6410933—control, SRR6410932—CUS) [53]. The protocol comprised 14 days with two random stressors per day; tissue was collected 24 h after the last stressor, at which point cortisol did not differ from control according to the original publication. The original design used three pooled libraries per group, four telencephala each. CUS protocols for adult *D. rerio* have since been standardised [54]; the original study predates this standard.

Run metadata were obtained with sra-tools 3.4.1 (NCBI, Bethesda, MD, USA). The runs were assembled by the ‘bam-load’ loader from BAM files; the original read identifiers had been replaced with sequential numbers and read group records were absent. The deposited alignments were produced with HISAT2 v2.0.5 against GRCz10 with ‘--no-spliced-alignment’ and ‘--rna-strandness R’, although the original publication states Bowtie2 v2.3.2.2. Observed read lengths were 20–151 bp against a nominal 150 bp, indicating trimming before submission. The aligned fraction was 63.7% (control) and 64.2% (CUS).

The RNA-seq material of the source study came from a separate cohort of 24 adult males; four telencephala with olfactory bulbs were pooled per sample, and three samples were prepared per group, so each condition is represented by three pools of four fish. Housing and the level at which each stressor was applied are discussed in Section 3.9.

### 4.2. Recovery of Library Structure

The runs were scanned in steps of 10^6^ spots (samples of 200 spots) along the ‘ALIGNMENT_COUNT’ column of the SEQUENCE table. Each run contained three contiguous blocks of fully unaligned read pairs. This structure corresponds to sequential loading of three BAM files: the loader numbers the aligned spots of each file consecutively and places the unaligned ones at the end of the corresponding block. Boundaries were located by bisection with verification of neighbouring spot states. Reads were extracted with ‘fasterq-dump’ 3.4.1, retaining the spot number in the identifier (‘--seq-defline “@$ac.$si/$ri”’, ‘--split-3’), and split line by line at four lines per read.

### 4.3. Quantification and Differential Expression

Quality control used Falco 1.3.2 (University of Southern California, Los Angeles, CA, USA) [55] summarised with MultiQC 1.35 (Seqera Labs, Barcelona, Spain). Alignment used STAR v2.7.11b (Cold Spring Harbor Laboratory, Cold Spring Harbor, NY, USA) [56] with the GRCz11/danRer11 index, and Ensembl release 112 annotation (EMBL-EBI, Hinxton, UK) [57] with chromosome names converted to UCSC notation, ‘--sjdbOverhang 149’, ‘--quantMode GeneCounts’, spliced alignment enabled. We used the reverse-strand count column in accordance with library strandedness: its ‘N_noFeature’ value is 3.5 times lower than that of the forward strand. Per-gene counting was performed by the built-in STAR function, equivalent to featureCounts [58].

Differential expression was analysed using DESeq2 v2.11.40.8 (Bioconductor; University of North Carolina, Chapel Hill, NC, USA) [59] on the Galaxy platform 2.11.40.8, (usegalaxy.eu, Freiburg, Germany), with the design set to ‘~condition’, the Wald test, Benjamini–Hochberg correction [60], an FDR threshold < 0.05, no shrinkage of log_2_FC estimates, and fitType set to parametric. Statistics were computed for 20,863 of 32,061 genes; size factors ranged from 0.86 to 1.09. The computation was independently repeated with an alternative implementation, PyDESeq2 0.5.4 (Owkin, Paris, France) [61], using the same parameters. Biological independence of the replicates was assessed from asymptotic and median dispersion, principal component analysis of the 500 most variable genes, and Spearman correlations between libraries.

### 4.4. Comparison with Independent Gene Sets

Glucocorticoid-dependent genes were taken from Eachus et al. [52], GSE213505: *nr3c1* knockout versus wild type, whole brain, *n* = 6 per group. From the full table of 4483 genes, we formed the GR-induced set (2482 genes, down-regulated in the mutant) and the GR-repressed set (2001 genes). The profile of CUS of a different duration came from Demin et al. [26]: 5-week protocol, whole brain, *n* = 6–7, 196 DEGs, together with the fluoxetine contrasts. Neuroinflammation without stress exposure came from Bhattarai et al. [62], GSE74326: intraventricular injection of Aβ42 in adult fish, telencephalon, two cell fractions with three replicates each; differential expression was computed by us (PyDESeq2, design ~ fraction + treatment, contrast Aβ42 versus control), since GEO holds a count matrix.

Enrichment analysis followed the GSEA (Stanford University, Stanford, CA, USA) approach on a pre-ranked list [63]: ranking by the Wald statistic, 17,704 genes with baseMean ≥ 10, 2000 permutations, FDR correction. Gene sets were formed at *padj* < 0.05 and split by direction of effect. DEG lists were additionally compared directly, by directional concordance (binomial test) and by correlation of effect-size estimates.

### 4.5. Associative Gene Network

We used stringApp 2.2.0 (Novo Nordisk Foundation Center for Protein Research, Copenhagen, Denmark) [64,65] in Cytoscape 3.10.3 (Cytoscape Consortium, San Diego, CA, USA) [66] with STRING v12.0 (SIB Swiss Institute of Bioinformatics, Lausanne, Switzerland) [67], *Danio rerio*, full network, all evidence sources, querying the 275 DEG identifiers. Networks were built at combined-score thresholds of 0.400 and 0.700, with and without up to 50 interactor proteins; the main analysis used the network without interactors at 0.400. Combined scores after excluding a source were recomputed with the STRING formula. Node names in Figure 1 are those carried by STRING v12.0, which maintains its own preferred names and does not track the current Ensembl release: chrna7 and ifi46 are symbols that ZFIN has since superseded by chrna7a and ifi27.4. We reproduce the figure as the software generated it and use the current symbols in the text and in Table 3; gene symbols elsewhere in this paper follow Ensembl 112.

Clustering used clusterMaker2 (University of California, San Francisco, CA, USA) [68,69] with MCL [70] (inflation 2.5, weights from ‘stringdb::score’, cutoff 0.4) and MCODE 2.0.3 (University of Toronto, Toronto, ON, Canada) [71] (degree cutoff 2, node score cutoff 0.2, K-core 2, haircut). Only clusters of at least five nodes were interpreted. Degree and betweenness centrality are reported within clusters only.

Two null models were used. Degree-preserving double edge swap [72] implemented in NetworkX 3.6.1 (https://networkx.org) (1000 iterations) tests whether a cluster is denser than the topology implies. Random gene sets matched for node degree in the full *D. rerio* network (1000 iterations, drawn from the 19,364 tested genes mapped to STRING) test whether the query list is more tightly connected than genes of comparable study coverage, since interactome data are biased towards well-studied proteins [50,51]. Robustness to the degree-binning scheme was checked in three ways.

For each cluster, we also determined edge composition by evidence source, the share of paralogue edges (Ensembl Compara release 116 (EMBL-EBI, Hinxton, UK)) [73], and connectivity after removing literature-only edges, after removing the highest-degree node, and after collapsing paralogue groups.

### 4.6. Functional Enrichment

g:Profiler version e114_eg62_p19 (University of Tartu, Tartu, Estonia) [74,75], organism *Danio rerio*, sources GO (MF, CC, BP), KEGG and Reactome, g:SCS correction, threshold 0.05. The background was the 20,863 genes for which DESeq2 computed an adjusted *p*-value; 20,859 resolved in g:Profiler. We defined the background as the set of tested genes rather than by a baseMean threshold: at a threshold of 10, the query was not a subset of the background, since 18 of the 275 DEGs had a baseMean below it, including *col17a1a*, *krt17* and *upk1a*, which belong to the interpreted clusters.

### 4.7. Null Models and Composition Analysis

Tissue-content scores were the mean log_2_ of DESeq2-normalised counts over marker panels of zebrafish genes (Table S17); genes belonging to the 275 DEGs were excluded from every panel. Group differences were tested by two-sample t-test and corrected across the twelve panels by the Benjamini–Hochberg procedure, with leave-one-library-out and leave-one-marker-out reported alongside. For the orthogonal-covariate model, the score was centred within each group, which makes it orthogonal to the group factor; DESeq2 was then fitted with design ~ group + score and the two contrasts tested separately. The collinearity control drew random covariates constrained to the same absolute correlation with the group factor as the measured score (*n* = 31). Genes were assigned to a non-neural tissue from three published studies of zebrafish expression—[27] for the keratins and E-cadherin, [29] for type XVII collagen and [28] for ictacalcin—together with the curated expression records of ZFIN (University of Oregon, Eugene, OR, USA; accessed 23 October 2026), consulted for every gene. The evidence is graded in Table S18b on the zebrafish record itself: tier A, expression recorded in the attributed tissue in adult fish with no neural record; tier B, zebrafish expression in that tissue at embryonic or larval stages, or not resolved to a stage; tier C, a record that also includes expression in adult brain or nervous system, so that it does not discriminate the origin of the transcript; tier D, no zebrafish expression annotation, the assignment resting on orthology to the mammalian gene. The eight genes set aside on the count pattern are tier E and carry no expression evidence. Two cautions apply to this material: ZFIN lists *col17a1b* among the former names of *col17a1a*, so legacy citations must be checked against the identifier and not the symbol, and [28] annotates *icn* but not *icn2*, whose assignment follows that of its paralogue.

For the genomic-clustering null, gene coordinates were taken from Ensembl 112. The 275 DEGs were binned into deciles of mean normalised expression defined on the non-DEG background; each of 2000 random sets drew the same number of non-DEG genes per decile. A pair was counted when two genes of a set were consecutive in start-position order on the same chromosome, and their start positions lay less than 200 kb apart; a pair was same-direction when the signs of their log_2_ fold changes agreed. The same function computed the observed and the null counts, so the comparison does not depend on the choice of distance measure. Gene symbols throughout follow Ensembl 112; where the annotation used by [1] gave a different symbol, both are reported (Table S34 and S34b). The reported *p*-value is the proportion of random sets reaching the observed count. For the gene-set mirror null, 2000 disjoint pairs of random gene sets of the same sizes as the two glucocorticoid sets were drawn from the ranked Wald statistics and tested by the same competitive procedure; the reported quantity is the number of draws in which both members reached *p* < 0.05 with opposite directions. Gene-set tests used the parametric branch of camera with inter-gene correlation fixed at 0.01, re-implemented in Python 3.13.16 (Python Software Foundation, Wilmington, DE, USA) with NumPy 2.5.3 and SciPy 1.18.1 (NumFOCUS, Austin, TX, USA); the reference implementation, limma 3.58.1 (Walter and Eliza Hall Institute, Melbourne, Australia) under R 4.3.3 (R Foundation for Statistical Computing, Vienna, Austria), reproduced the results. Two different gene-set procedures appear in this paper and are not pooled. Table 4 and Figures S6–S8 report the pre-ranked GSEA of Section 4.4, a self-contained test on the ranked list with 2000 permutations of gene labels. The glucocorticoid and marker-panel tests (Tables S19a,b, S22, S28, S30 and S33) use the competitive camera procedure described here, which compares the genes of a set against the remaining genes and accounts for inter-gene correlation. Where both are reported for the same gene set, the procedure is named in the text. The composition, genomic-clustering and mirror-null analyses were run in the same Python environment with pandas 3.0.5 and statsmodels 0.15.0; Supplementary Figures were drawn with matplotlib 3.11.2.

## 5. Conclusions

1. A 14-day CUS protocol coordinately reduces GR-dependent transcription in the *D. rerio* telencephalon: GR-induced genes shift down and GR-repressed genes up, mirror-symmetrically, while cortisol is still at control level. Tissue composition is not excluded as an alternative explanation of this shift (Section 3.9).
2. Coordinately changed genes form robust network clusters corresponding to TGF-β signalling, collagens and nucleotide metabolism. Keratins and barrier structures do not form such a cluster once transcripts of probable epidermal origin are set aside.
3. Matrix changes are isoform-specific: single paralogues change while their duplicates do not, and perineuronal nets are unaffected. After the reassignment in Section 2.11, the surviving enrichment is for collagen trimer rather than for the basement membrane.
4. Antioxidant and monoamine oxidase transcripts are unchanged; changes reported for these systems in stress models are likely realised downstream of transcription.
5. The 14-day profile disagrees with the 5-week one and overlaps only weakly with a neuroinflammatory signature, so DEG lists from different exposure durations cannot serve as mutual validation.
6. Network connectivity exceeds chance only under study-bias control, because stress-responsive genes are less studied than the transcriptome background.
7. Replicate structure lost on submission is recoverable from the block structure of unaligned reads; the procedure is verifiable and applies to any record of this kind.
8. Part of the gene list reflects composition and genomic structure rather than regulation: epidermal content differs between the groups while neuronal content does not, and the list is clustered in the genome far beyond chance. In pooled designs without genotyping, tissue carry-over and segregating structural variation are not distinguishable from regulation at the level of the individual gene. The glucocorticoid result is not removed by setting these transcripts aside, but that removal speaks to the origin of individual transcripts and not to the composition of the samples: with three pools per group and epidermal content collinear with the treatment, a between-group difference in composition remains an alternative explanation and is not excluded here.
9. Seven hypotheses follow, each with a proposed experimental test. They do not share one design: some require genotyping of the pooled animals, others immunostaining, enzyme activities or measurement of metabolite flux.

## Figures and Tables

**Table 1:** Recovered libraries and realignment results. “Junction-spanning reads, M” is the number of reads aligned across an exon–exon junction, in millions; it is not the number of distinct junctions. Of the distinct junctions detected, 94–95% were annotated. The deposited alignment excluded spliced reads and reached an aligned fraction of 63.7–64.2%.

Group	Library	Spot Range	Read Pairs	Uniquely Aligned, %	Multi-Mapped, %	Junction-Spanning Reads, M
Control	1	1–27,540,277	27,540,277	81.6	10.8	16.6
Control	2	27,540,278–53,577,688	26,037,411	81.4	11.0	15.0
Control	3	53,577,689–76,704,853	23,127,165	81.2	11.0	13.4
CUS	1	1–29,166,077	29,166,077	80.6	11.6	15.6
CUS	2	29,166,078–55,378,560	26,212,483	81.5	11.2	15.5
CUS	3	55,378,561–76,913,951	21,535,391	81.5	11.1	13.1

**Table 2:** Comparison with the original publication. Chance agreement exceeds 50% because the distribution of log_2_FC is shifted towards down-regulation.

Measure	Value
Statistics computed	20,863 of 32,061 genes
DEGs at FDR < 0.05	275 (139 ↑/136 ↓)
Spearman correlation of log_2_FC (15,690 genes with baseMean ≥ 10 in both analyses)	ρ = 0.754
Published DEGs detectable here/agreeing in direction	134/132 (98.5%)
Chance expectation of directional agreement	80.0% (*p* < 0.001)
Published DEGs reaching FDR < 0.05 here	101
DEGs not in the published list (275—101)	174

**Table 3:** Clusters of at least five nodes: composition, distribution of edges by evidence source and the most significant enrichment term (g:Profiler, background of 20,863 tested genes, g:SCS). Full term lists are in Table S8; node labels without official symbols are in Table S9.

Cluster	Genes (log_2_FC)	exp/db/coexp/txt-only	Leading Term (*p*)
MCL 1 (9)	*smad3a* (−2.36), *irf3* (+2.08), *smad6a* (−1.66), *nog2* (+0.73), *hey1* (+0.73), *tgfb1a* (+0.70), *jun* (−0.69), *gpx1a* (−0.67), *cdk5* (+0.53)	4/2/5/4	TGF-β signalling, KEGG:04350 (2.9 × 10^−4^)
MCL 2 (8)	*col17a1a* (−6.26), *col8a2* (+1.45), *p3h1* (−1.04), *col16a1* (+0.99), *col4a6* (+0.98), *col12a1a* (+0.79), *lamb2l* (+0.78), *bmp1b* (+0.64)	6/15/7/0	Collagen trimer, GO:0005581 (2.0 × 10^−3^)
MCL 3 (7)	*krt17* (−4.03), *krt4* (−3.62), *krt5* (−3.29), *pkp1b* (−2.69), *zgc:136930* (−2.46), *tfpt* (+0.93), *hephl1a* (+0.58)	7/3/7/1	Intermediate filament, GO:0005882 (1.3 × 10^−5^)
MCL 4 (6)	*ela2l* (+8.30), *loxl2b* (−2.80), *her4.5* (+2.76), *chrna7a* (−1.74), *ifi27.4* (+1.46), *her8.2* (+1.45)	4/0/0/6	None
MCL 5 (5)	*ampd1* (+3.73), *entpd8* (−2.38), *tk2* (−0.71), *rrm2b* (+0.69), *mcm7* (−0.45)	0/3/2/0	Nucleobase compound metabolism, GO:0006139 (2.2 × 10^−3^)
MCL 6 (5)	*upk1a* (−5.33), *X1WG46* (−4.30), *ENSDARP00000131071* (−2.79), *F1QEW2* (−2.60), *moxd1* (+1.15)	1/0/4/3	None

**Table 4:** Enrichment analysis on the full ranked list (17,704 genes, 2000 permutations; Figures S6 and S7). Size is the number of set genes represented in the ranking.

Gene Set	Source	Size	NES	FDR
GR-induced genes	[52]	2215	−2.11	<0.001
GR-repressed genes	[52]	1776	+1.91	4 × 10^−4^
Down-regulated under 5-week CUS	[26]	88	+1.96	6 × 10^−4^
Down-regulated under fluoxetine	[26]	23	+2.17	<0.001
Up-regulated under fluoxetine	[26]	10	−1.54	0.035
Up-regulated under Aβ42	[62]	2146	+1.36	0.040
Down-regulated under Aβ42	[62]	2660	−1.21	0.150
Up-regulated under 5-week CUS	[26]	32	−0.65	0.96

## Data Availability

The raw reads are available in NCBI SRA (BioProject PRJNA422916). External datasets used: GSE213505, GSE74326. The boundaries of the recovered libraries are given in Table 1 and suffice to reproduce the splitting procedure in full. The per-gene read count matrix, the complete DESeq2 results table, gene lists and gene set files for the enrichment analysis are provided in the Supplementary Materials. The original contributions presented in this study are included in the article/Supplementary Materials. Further inquiries can be directed to the corresponding author.

## Supplementary Materials

The following supporting information can be downloaded at: https://www.mdpi.com/article/10.3390/ijms27219772/s1.
