# Supplementary material for: Chronic Stress Attenuates the Glucocorticoid Receptor Transcriptional Programme in the Danio rerio Telencephalon
Source: Int J Mol Sci. 2026 Oct 30;27(21):9772. doi: 10.3390/ijms27219772 (PMC13762656; doi:10.3390/ijms27219772)
Supplement: Supplementary file 1 [file ijms-27-09772-s001.zip › ijms-4631408-supplementary.pdf]

Supplementary Materials

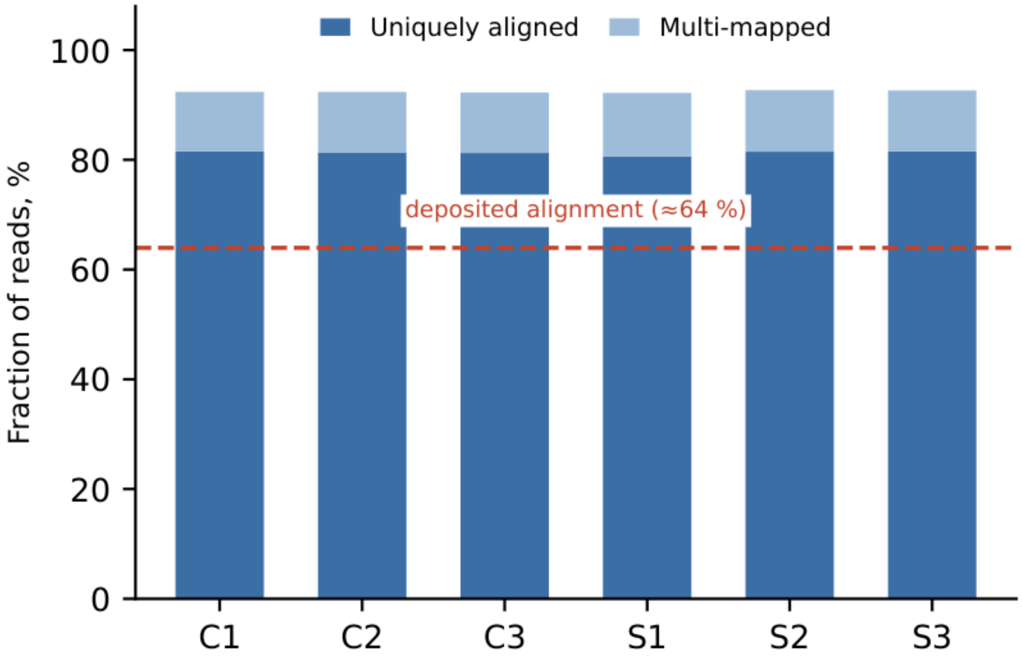

Figure S1. Fraction of aligned reads by library. Bars show uniquely aligned and multi-mapped reads (STAR, GRCz11 assembly, spliced alignment). The dashed line marks the aligned fraction of the deposited alignment (HISAT2, GRCz10, without splicing).

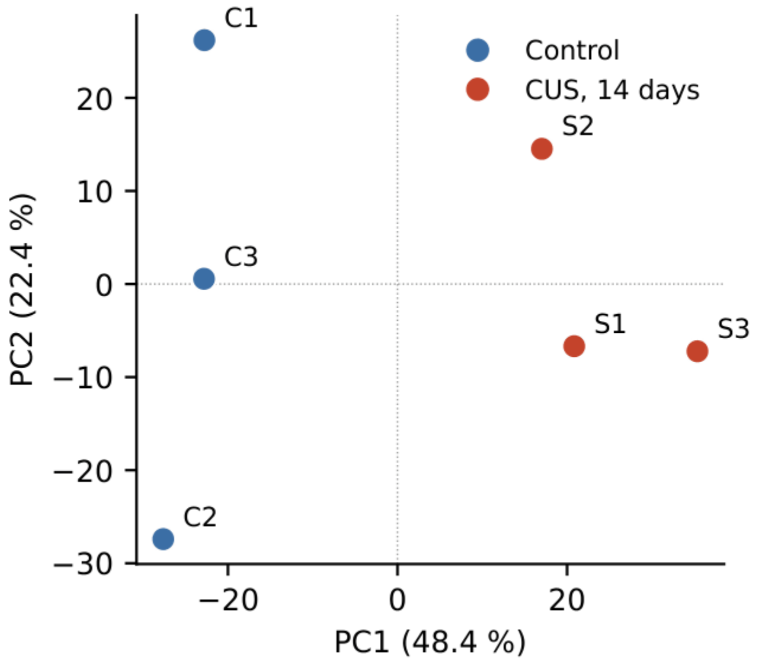

Figure S2. Principal component analysis of the 500 most variable genes (log-scaled DESeq2 normalised expression). The first component (48.4 % of variance) separates the groups completely; the second (22.4 %) is unrelated to grouping. C1–C3, control libraries; S1–S3, libraries of the chronic unpredictable stress group.

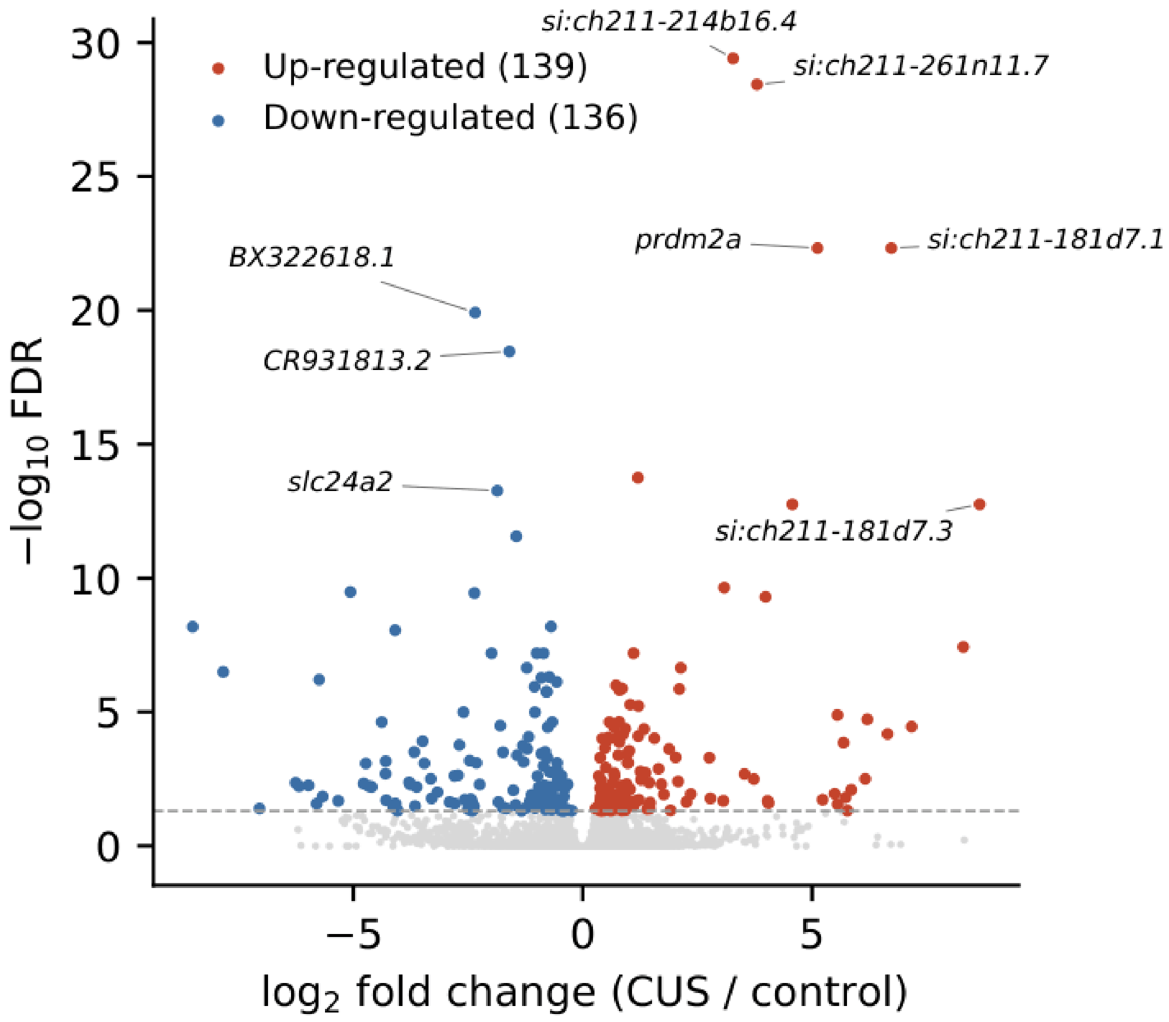

Figure S3. Differential expression under chronic unpredictable stress. The x-axis shows $\log_2$ fold change (stress relative to control), the y-axis the negative decimal logarithm of the adjusted p-value. Genes at FDR < 0.05 are highlighted (139 up-regulated and 136 down-regulated); the dashed line marks the FDR = 0.05 threshold. The eight most significant genes are labelled. Y-axis values are capped at 35.

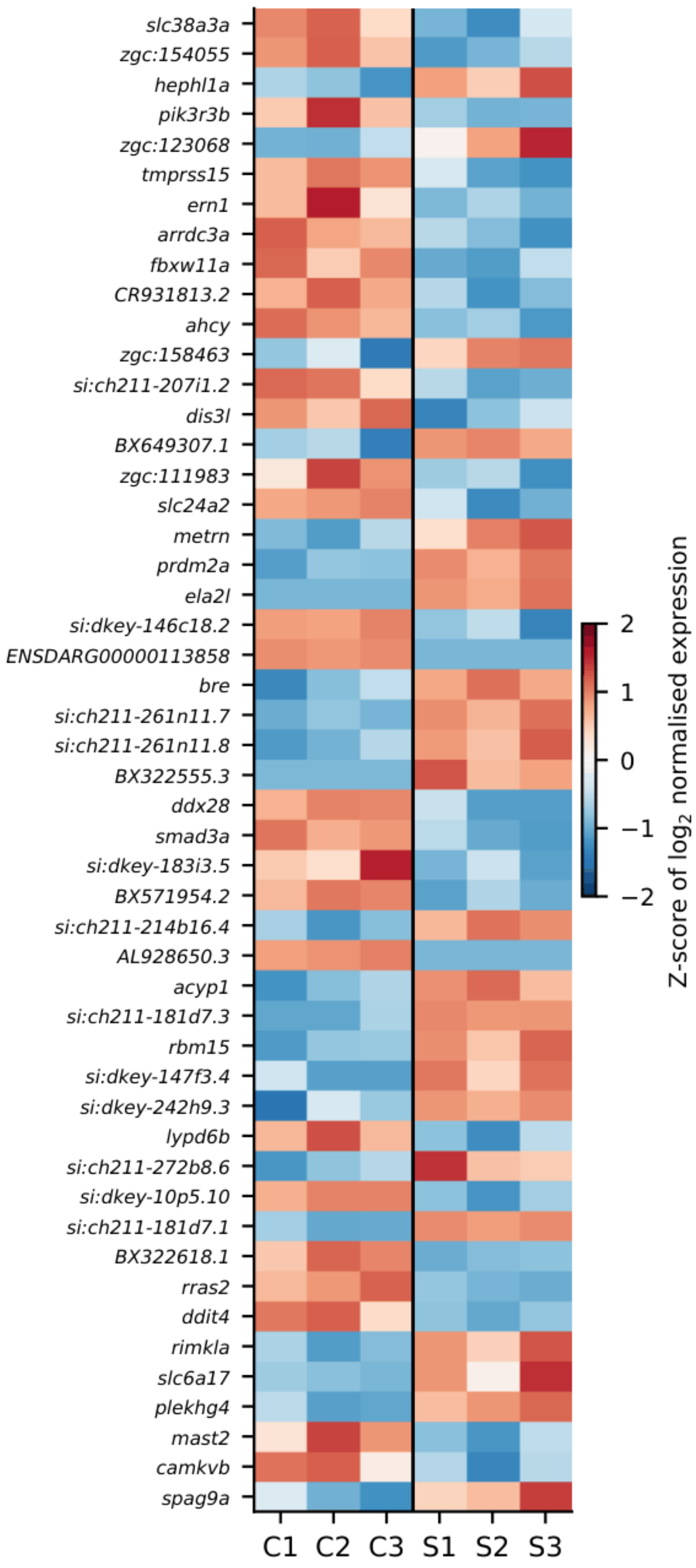

Figure S4. The fifty most significant DEGs. Z-transformed log-scaled normalised expression values are shown; genes are ordered by mean value. The vertical line separates the groups.

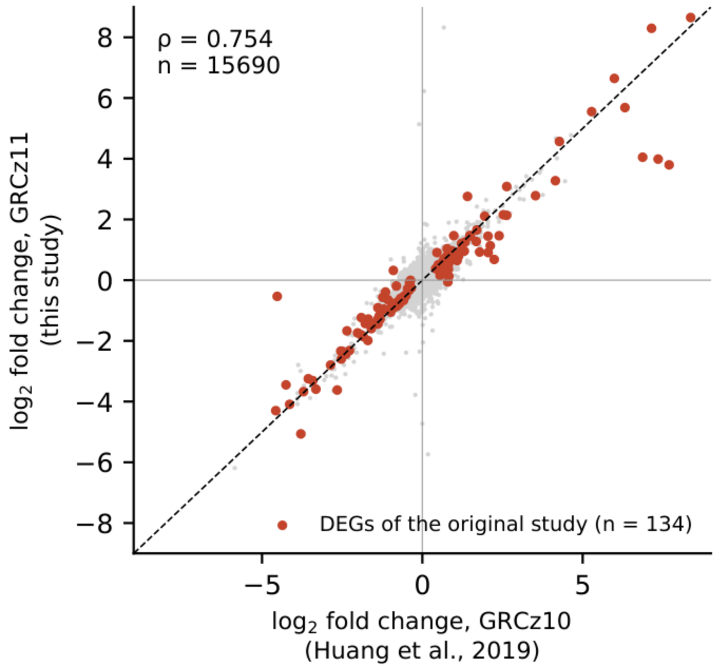

Figure S5. Comparison of effect-size estimates with those previously published. Axes show $\log_2$ fold change for the 15,690 genes detectable in both analyses. Red marks the 134 genes from the list of 155 DEGs of the original publication that are detectable in the present analysis; the dashed line is the line of equality.

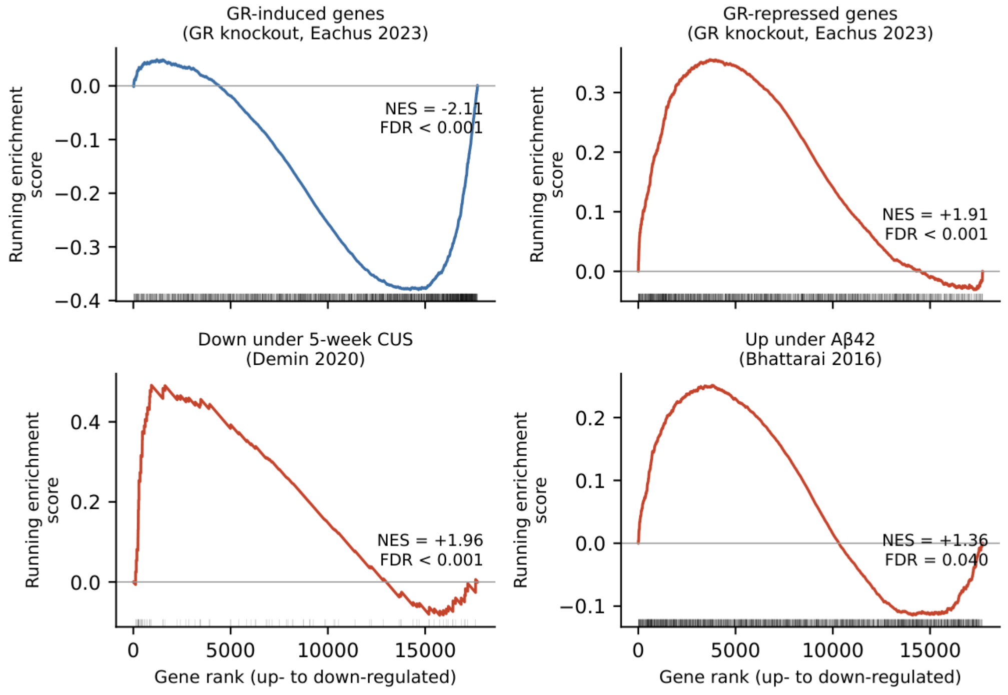

Figure S6. Enrichment curves for four external gene sets. The 17,704 genes are ranked by the Wald statistic from up- to down-regulated; ticks below each curve mark the positions of set genes in the ranking. The normalised enrichment score (NES) and FDR are given (2,000 permutations).

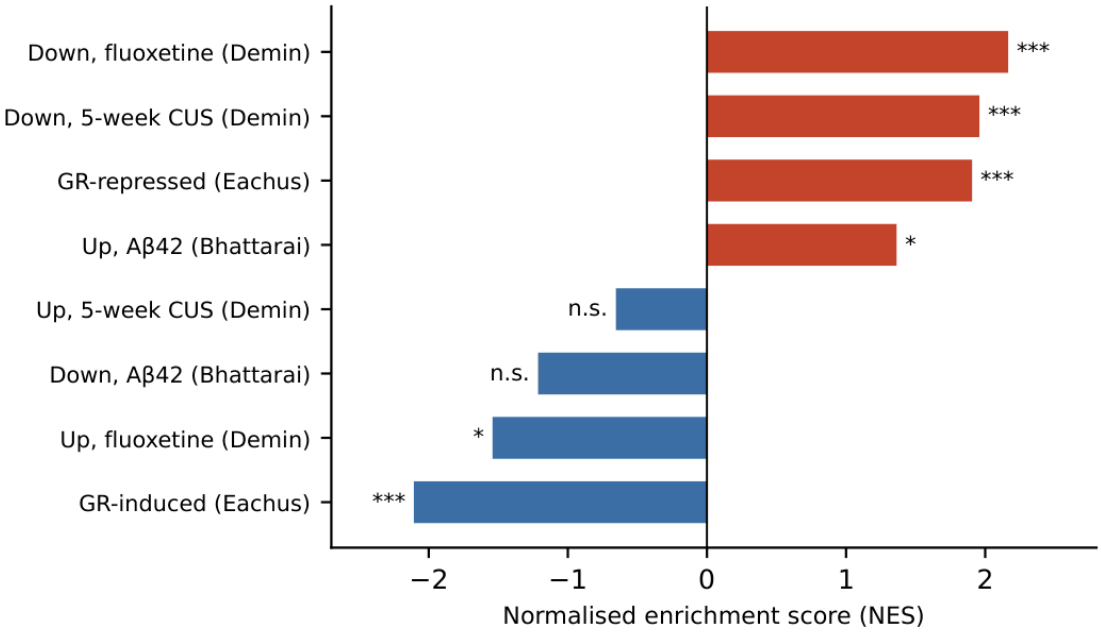

Figure S7. Summary of enrichment analysis for eight gene sets. Significance: * FDR < 0.05; ** FDR < 0.01; *** FDR < 0.001; n.s., not significant.

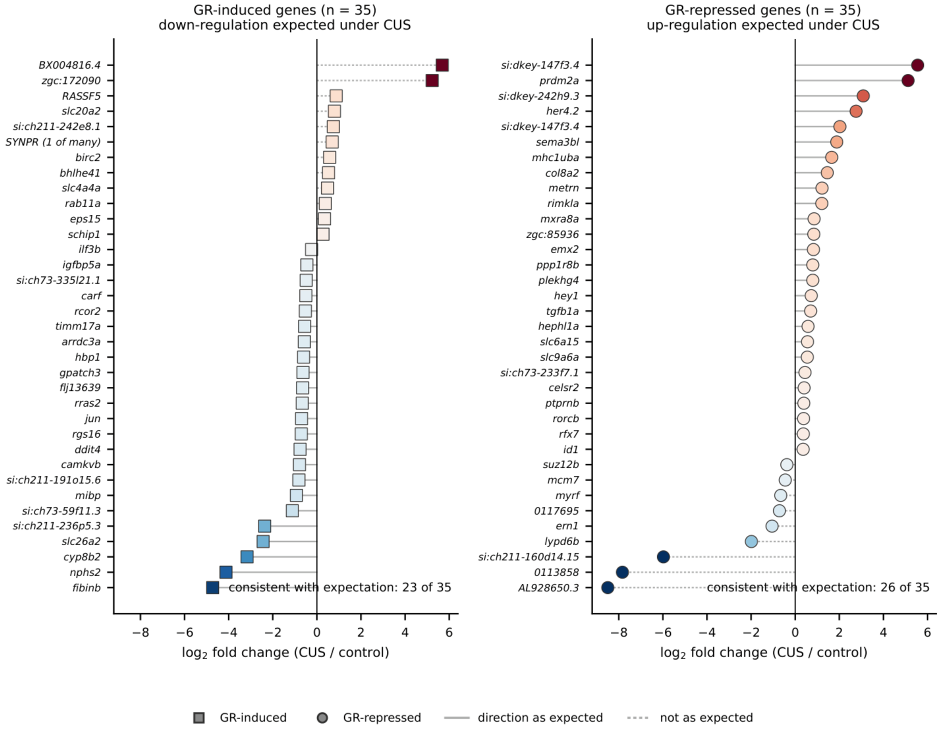

Figure S8. DEGs belonging to the sets of glucocorticoid-dependent genes of *Danio rerio* (Eachus et al., 2023): 70 of 275. Square markers denote GR-induced genes (35), circles GR-repressed genes (35). Marker colour is $\log_2$ fold change. Dashed lines mark genes whose direction of change does not match the expectation for that set (12 of 35 induced and 9 of 35 repressed). The significance of the shift in the GR-dependent programme was established by enrichment analysis on the full ranked gene list (NES = −2.11 and +1.91; FDR < 10^{-3}), not from this set.

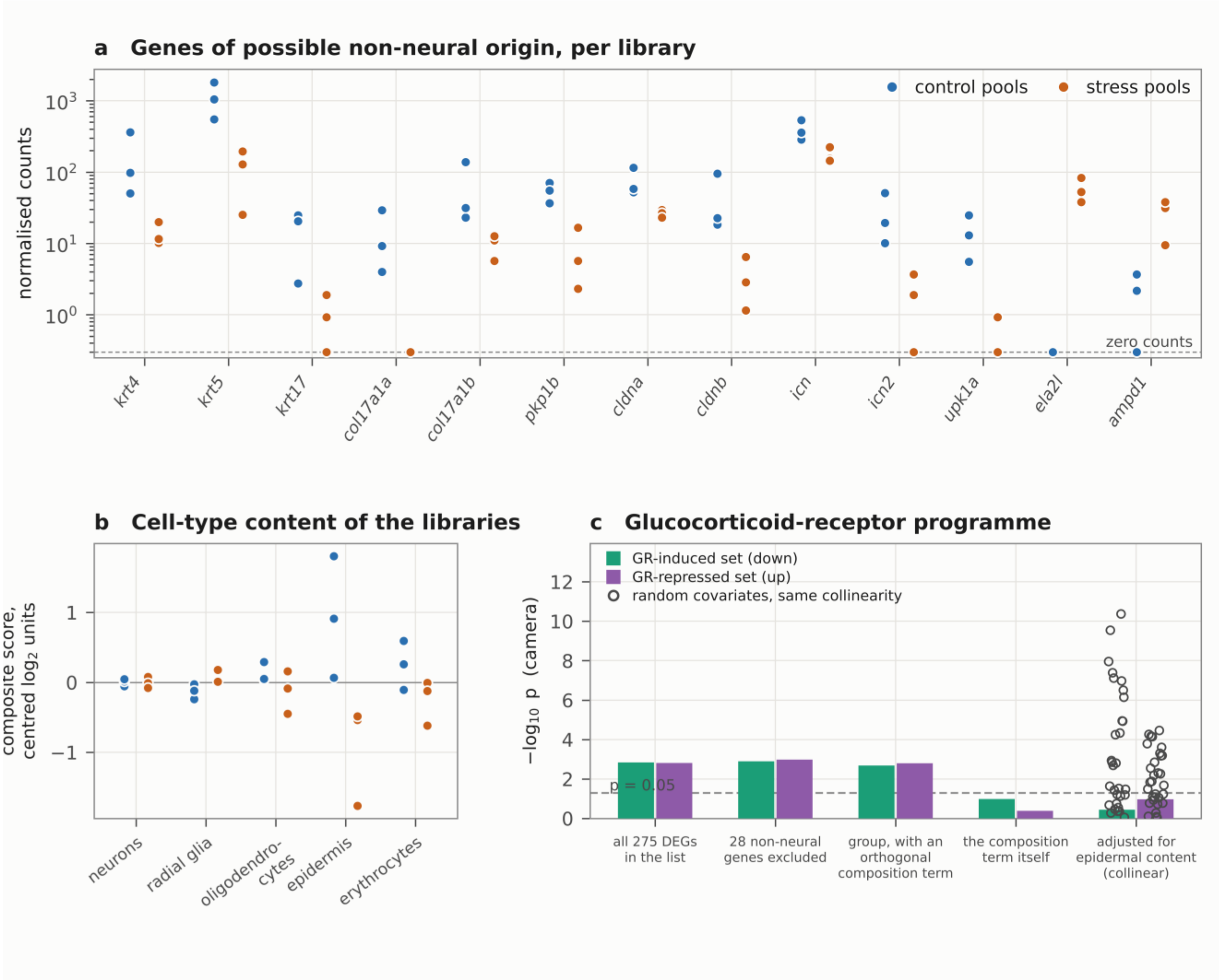

Figure S9. Tissue composition of the libraries and its relation to the glucocorticoid result. (a) Per-library normalised counts for the genes raised by the reviewer, on a logarithmic scale; control pools in blue, stress pools in orange. (b) Composite scores for five of the twelve marker panels, centred within panels and expressed in $\log_2$ units; the epidermal panel is the only one showing a substantial difference between groups, and the neuronal panel shows none; all twelve are in Table S22. (c) Competitive tests of the two glucocorticoid-dependent gene sets across five models, with the distribution obtained from thirty-one random covariates of equal collinearity shown for comparison.

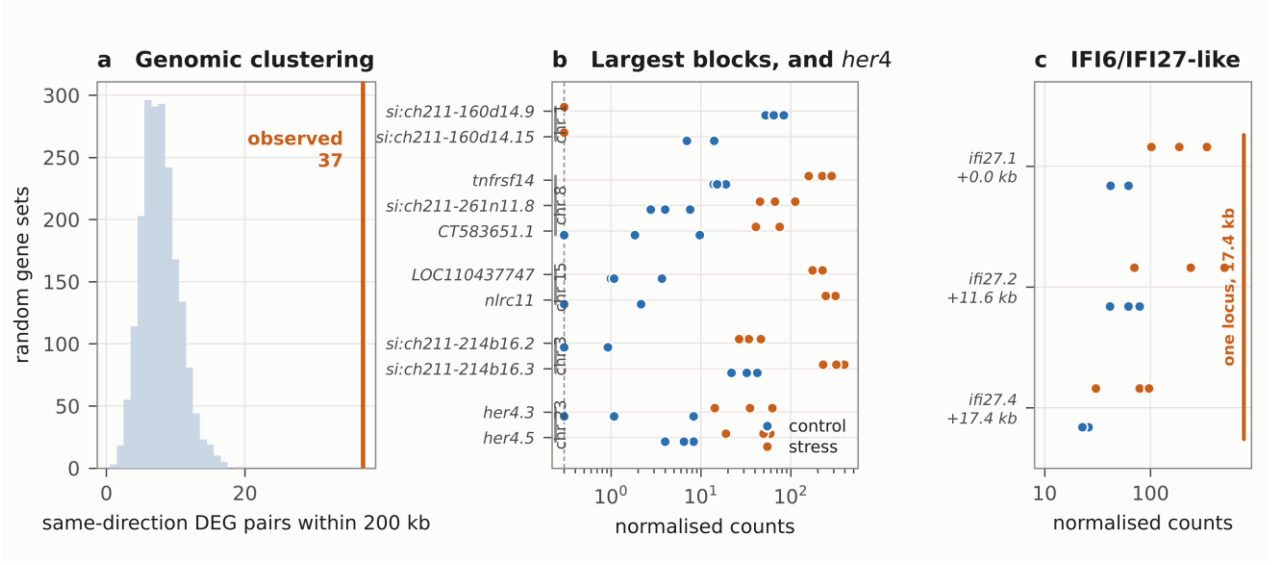

Figure S10. Genomic distribution of the differentially expressed genes. (a) Same-direction gene pairs within 200 kb among the 275 DEGs against 2,000 random gene sets matched decile by decile on mean normalised expression. (b) Per-library normalised counts for the largest positional blocks and for the her4 pair on chromosome 23; symbols follow Ensembl 112. (c) The three genes carrying the Interferon-induced 6–16 domain, with their distances from the first; they occupy a single locus of 17.4 kb.

Table S1. Biological independence of the replicates

Measure	Value	Interpretation
Asymptotic dispersion (a_0)	0.0056	Near zero for technical replicates
Median per-gene dispersion	0.0041	As above
Variance on PC1	48.4%	Groups fully separated
Variance on PC2	22.4%	Unrelated to grouping
Spearman correlation within groups	0.9765–0.9839	Exceeds between-group values
Spearman correlation between groups	0.9765–0.9831	—
Size factors	0.86–1.09	Uniform sequencing depth

Table S1. Measures of biological independence of the replicates.

Table S2. Most significant additionally identified genes

Gene	baseMean	$\log_2$FC	padj
prdm2a	83.2	+5.12	4.7×10^{-23}
rimkla	1103.7	+1.21	1.8×10^{-14}
smad3a	64.3	−2.36	3.6×10^{-10}
pik3r3b	534.2	−1.00	6.3×10^{-8}
slc6a17	1156.2	+1.11	6.3×10^{-8}
si:dkey-10p5.10	148.9	−1.22	2.2×10^{-7}
slc38a3a	2749.9	−0.73	4.9×10^{-7}
ddx28	27.0	−5.74	6.2×10^{-7}

Table S2. Most significant genes not identified in the original analysis.

Table S3. State of individual functional systems

System	Changed genes	Unchanged genes (tested)
Basement membrane, type IV collagen	*col4a6* (+0.98)	*col4a1*, *col4a2*, *col4a3*, *col4a4*, *col4a5*
Laminins	*lamb2l* (+0.78)	*lamb1a*, *lamb1b*, *lamb2*, *lama1*, *lama2*, *lama4*, *lama5*, *lamc1*
Other membrane components	—	*nid1a*, *nid1b*, *nid2a*, *hspg2*, *col15a1b*, *fn1a*, *fn1b*, *sparc*
Collagen maturation	*p3h1* (−1.04), *bmp1b* (+0.64), *loxl2b* (−2.80)	*p3h2*, *plod1a*, *plod2*, *bmp1a*, *loxl2a*, *loxl1*
Hemidesmosomes	*col17a1a* (−6.26), *col17a1b* (−2.72)	*col7a1*
Perineuronal nets	—	*acana*, *acanb*, *bcan*, *vcana*, *vcanb*, *tnr*, *hapln1a*, *hapln1b*, *hapln3*, *hapln4*, *ptprz1a*, *ptprz1b*, *has3*, *mmp2*, *mmp9*, *adamts5*, *otx2*
Antioxidant defence	*gpx1a* (−0.67)	*gpx1b*, *gpx4a*, *gpx4b*, *sod1*, *sod2*, *cat*, *prdx1*, *prdx2*, *txn*, *txnrd1*, *gsr*, *nqo1*, *hmox1a*, *nfe2l2a*, *keap1a*
Monoamine oxidase and GR axis	—	*mao*, *nr3c1*, *nr3c2*, *hsd11b2*
Glutamine–glutamate metabolism	*slc38a3a* (−0.73), *slc6a17* (+1.11), *camkvb* (−0.79)	*slc38a3b*, *slc38a2*, *slc1a2b*, *slc1a3a*, *slc1a3b*, *glula*, *glulb*, *glulc*, *gad1b*, *gad2*, *slc17a6a*, *slc17a6b*, *slc17a7a*, *grin1a*, *camk2g1*
Nucleotide metabolism	*ampd1* (+3.73), *entpd8* (−2.38), *tk2* (−0.71), *rrm2b* (+0.69), *mcm7* (−0.45)	*entpd1*, *entpd2a*.1, *entpd3*, *entpd4*, *entpd5a*, *entpd6*, *nt5e*, *adora1b*, *adora2aa*, *adora2ab*, *p2rx7*, *p2ry1*, *p2ry12*, *pnp4a*, *ada*

Table S3. Functional systems assessed against predefined gene lists. $\log_2$FC values are given in parentheses. Genes were considered unchanged at an adjusted p-value ≥ 0.05. *slc1a2a*, *adora1a*, *p2rx4a* and *has2* were removed by DESeq2 independent filtering and were not tested.

Table S4. Sensitivity of the network to threshold and evidence sources

Sources	Threshold	Edges	Nodes	DEGs, %	Components	Largest componen t
All	0.400	137	118	46.1	19	61
All	0.700	29	39	15.2	13	6
All	0.900	8	11	4.3	4	4
Without textmining	0.400	47	48	18.8	16	6
Without textmining	0.700	10	15	5.9	6	4
Without textmining and coexpression	0.400	42	45	17.6	16	6
Experiments + databases only	0.400	41	43	16.8	15	6

Table S4. Sensitivity of the network to the threshold and the set of evidence sources. Only nodes with at least one edge are counted. Combined scores after exclusion of a source were recomputed with the STRING formula. The genomic neighbourhood, gene fusion and co-occurrence sources did not change the network by a single edge at a threshold of 0.700.

Table S5. Null model of random gene sets

Model	Statistic	Observed	Expected	95 % interval
Degree-matched	Internal edges	137	64.8	50–80
Degree-matched	Largest component	61	19.6	8–41
Unmatched (control)	Internal edges	137	119.9	80–173
Unmatched (control)	Largest component	61	59.3	—

Table S5. Null model of random gene sets (1,000 iterations). Robustness to the degree-binning scheme was checked with three schemes: expected edge counts of 65.9 / 71.8 / 71.4, $p < 0.001$ in all cases.

Table S6. Betweenness centrality: full result

Gene	Degree	Betweenness	Expected	p	Cluster
cdh1	8	0.083	0.162	0.985	—
F1QEW2_DANRE	7	0.158	0.134	0.262	MCL 6
col8a2	7	0.055	0.134	0.999	MCL 2
chrna7	7	0.054	0.136	0.998	MCL 4
ifi46	6	0.134	0.110	0.234	MCL 4
krt5	6	0.058	0.106	0.945	MCL 3
jun	6	0.026	0.108	1.000	MCL 1
p3h1	6	0.009	0.108	1.000	MCL 2
col4a6	6	0.009	0.108	1.000	MCL 2
rrm2b	5	0.056	0.081	0.770	MCL 5
loxl2b	5	0.048	0.082	0.889	MCL 4
smad3a	5	0.025	0.082	0.999	MCL 1
col17a1a	5	0.000	0.082	1.000	MCL 2
col16a1	5	0.000	0.084	1.000	MCL 2
col12a1a	5	0.000	0.082	1.000	MCL 2
zgc:136930	4	0.069	0.058	0.266	MCL 3
ampd1	4	0.053	0.059	0.482	MCL 5
ela2l	4	0.053	0.058	0.488	MCL 4
cldnb	4	0.049	0.057	0.531	—
c4b	4	0.017	0.057	0.994	—
X1WG46_DANRE	4	0.012	0.057	0.995	MCL 6
ENSDARP00000131071	4	0.012	0.058	0.997	MCL 6
pik3r3b	4	0.003	0.058	0.994	—
krt4	4	0.002	0.059	0.995	MCL 3
plp1b	4	0.002	0.059	0.997	—
cldna	3	0.056	0.037	0.149	—
ahcy	3	0.041	0.036	0.313	—
eef2a.2	3	0.025	0.036	0.662	—
crocc2	3	0.025	0.036	0.686	—
entpd8	3	0.009	0.037	0.962	MCL 5
smad6a	3	0.009	0.037	0.970	MCL 1
rras2	3	0.003	0.035	0.984	—
krt17	3	0.002	0.035	0.983	MCL 3
metrn	3	0.002	0.036	0.983	—
myrf	3	0.001	0.036	0.991	—
cyp8b3	3	0.001	0.036	0.990	—
moxd1	3	0.000	0.036	1.000	MCL 6
tgfb1a	3	0.000	0.035	1.000	MCL 1
med7	2	0.033	0.015	0.075	—
kdm5ba	2	0.009	0.016	0.716	—
rab11a	2	0.009	0.016	0.722	—
acox1	2	0.000	0.017	0.918	—

rhbg	2	0.000	0.016	0.936	—
rcor2	2	0.000	0.015	0.937	—
dnah1	2	0.000	0.015	0.968	—
col17a1b	2	0.000	0.017	0.971	—
nphs2	2	0.000	0.016	0.966	—
slc43a2a	2	0.000	0.016	0.977	—
cdh16	2	0.000	0.016	1.000	—
cdh19	2	0.000	0.015	1.000	—
c7b	2	0.000	0.015	1.000	—
cd59	2	0.000	0.016	1.000	—
her4.5	2	0.000	0.016	1.000	MCL 4
her8.2	2	0.000	0.016	1.000	MCL 4
cyp8b2	2	0.000	0.016	1.000	—
B0UYB4_DANRE	2	0.000	0.016	1.000	—
hey1	2	0.000	0.016	1.000	MCL 1
klf7b	2	0.000	0.016	1.000	—
neurod6a	2	0.000	0.016	1.000	—
mast2	2	0.000	0.016	1.000	—
gpbp1l1	2	0.000	0.016	1.000	—
mpz	2	0.000	0.016	1.000	—
flj13639	2	0.000	0.017	1.000	—
tk2	2	0.000	0.017	1.000	MCL 5
ccdc40	1	0.000	0.000	1.000	—
suz12b	1	0.000	0.000	1.000	—
U3JAP2_DANRE	1	0.000	0.000	1.000	—
cenpo	1	0.000	0.000	1.000	—
F1QGA7_DANRE	1	0.000	0.000	1.000	—
schip1	1	0.000	0.000	1.000	—
dnah3	1	0.000	0.000	1.000	—
nog2	1	0.000	0.000	1.000	MCL 1
camkvb	1	0.000	0.000	1.000	—
ilf3b	1	0.000	0.000	1.000	—
tfpt	1	0.000	0.000	1.000	MCL 3
tmprss15	1	0.000	0.000	1.000	—
muc5.2	1	0.000	0.000	1.000	—
tor3a	1	0.000	0.000	1.000	—
hbba1	1	0.000	0.000	1.000	—
ba1l	1	0.000	0.000	1.000	—
zgc:92380	1	0.000	0.000	1.000	—
gpm6bb	1	0.000	0.000	1.000	—
tmem98	1	0.000	0.000	1.000	—
dysf	1	0.000	0.000	1.000	—
eps15	1	0.000	0.000	1.000	—
hephl1a	1	0.000	0.000	1.000	MCL 3
ctsd	1	0.000	0.000	1.000	—

birc2	1	0.000	0.000	1.000	—
pkp1b	1	0.000	0.000	1.000	MCL 3
slc6a15	1	0.000	0.000	1.000	—
arrdc3a	1	0.000	0.000	1.000	—
ythdc1	1	0.000	0.000	1.000	—
rbm15	1	0.000	0.000	1.000	—
niban1a	1	0.000	0.000	1.000	—
zgc:86896	1	0.000	0.000	1.000	—
bhlhe41	1	0.000	0.000	1.000	—
E7FFE4_DANRE	1	0.000	0.000	1.000	—
bmp1b	1	0.000	0.000	1.000	MCL 2
abhd8a	1	0.000	0.000	1.000	—
lamb2l	1	0.000	0.000	1.000	MCL 2
RASSF5	1	0.000	0.000	1.000	—
upk1a	1	0.000	0.000	1.000	MCL 6
Q6DRQ1_DANRE	1	0.000	0.000	1.000	—
slc4a4a	1	0.000	0.000	1.000	—
znf592	1	0.000	0.000	1.000	—
col28a1b	1	0.000	0.000	1.000	—
irf3	1	0.000	0.000	1.000	MCL 1
cdk5	1	0.000	0.000	1.000	MCL 1
gpx1a	1	0.000	0.000	1.000	MCL 1
arhgef18a	1	0.000	0.000	1.000	—
plekhg4	1	0.000	0.000	1.000	—
fat2	1	0.000	0.000	1.000	—
ackr3b	1	0.000	0.000	1.000	—
opn1mw2	1	0.000	0.000	1.000	—
ccdc120	1	0.000	0.000	1.000	—
mcm7	1	0.000	0.000	1.000	MCL 5
E7F0F4_DANRE	1	0.000	0.000	1.000	—
psat1	1	0.000	0.000	1.000	—

Table S7. Nodes are ordered by degree, then by centrality. Expected is the mean of the null distribution; p is the empirical one-sided p-value for exceeding the null level. For no node does the excess over the null level reach significance: the minimum empirical p-value is 0.075, for med7 (degree 2), and among high-degree nodes the minimum is 0.234, for ifi46 (degree 6). For most nodes the observed centrality falls below the null expectation. Nodes outside clusters of at least five members are marked "—" in the Cluster column.

Table S7. Permutation test by cluster

Cluster	Nodes	Internal edges	Expected	95 % interval	p
MCL 1	9	10	0.83	0–3	< 0.001
MCL 2	8	17	2.01	0–5	< 0.001
MCL 3	7	8	0.58	0–2	< 0.001
MCL 4	6	10	0.98	0–3	< 0.001
MCL 5	5	5	0.33	0–2	< 0.001
MCL 6	5	7	0.50	0–2	< 0.001
MCODE 1	6	15	1.77	0–4	< 0.001
MCODE 3	8	13	2.52	0–5	< 0.001

Table S7. Degree-preserving edge randomisation: 1,000 iterations, 1.25 million successful swaps. The test asks whether a cluster is denser than expected under the given topology; it does not test whether the cluster operates as a regulatory unit.

Table S8. Significant enrichment terms by cluster

Cluster	Term	ID	Genes	p (g:SCS)
MCL 1	TGF-beta signaling pathway	KEGG:04350	3	2.86×10^{-4}
MCL 1	27 further terms (GO:BP, GO:MF, Reactome)	—	—	< 0.05
MCL 2	Collagen trimer	GO:0005581	2	2.01×10^{-3}
MCL 2	ECM-receptor interaction	KEGG:04512	2	7.8×10^{-3}
MCL 2	Cornified envelope formation	KEGG:04382	2	1.7×10^{-2}
MCL 2	Integrin signaling	KEGG:04518	2	2.5×10^{-2}
MCL 3	Intermediate filament	GO:0005882	3	1.3×10^{-5}
MCL 3	Keratinization	GO:0031424	3	1.5×10^{-5}
MCL 3	Keratinocyte differentiation	GO:0030216	3	9.1×10^{-5}
MCL 3	Skin development	GO:0043588	3	2.3×10^{-4}
MCL 3	Keratin filament	GO:0045095	3	3.0×10^{-4}
MCL 3	20 further terms	—	—	< 0.05
MCL 5	Nucleobase-containing compound metabolic process	GO:0006139	5	2.16×10^{-3}
MCL 5	Nucleotide metabolism	KEGG:01232	3	2.6×10^{-3}
MCL 5	Purine metabolism	KEGG:00230	3	6.0×10^{-3}
MCL 5	6 further terms	—	—	< 0.05
MCL 4	Crosslinking of collagen fibrils (not significant)	REAC	1	0.125
MCL 6	Cell-matrix adhesion (not significant)	GO:0031012	1	0.997

Table S8. Enrichment by cluster (g:Profiler, background of 20,863 tested genes, g:SCS correction, threshold 0.05).

The single significant term across the full list of 275 DEGs is KEGG:04382, cornified envelope formation (term size 115, 9 query genes, $p = 1.07 \times 10^{-3}$). Term genes: *col17a1a* (−6.26), *krt17* (−4.03), *icn2* (−3.78), *cldnb* (−3.67), *col17a1b* (−2.72), *pkp1b* (−2.69), *cldna* (−1.51), *icn* (−1.18), *col4a6* (+0.98).

The miRTarBase source was excluded on the basis of a measurement: in an exploratory run over the 275 DEGs with the source enabled, 1,337 terms were returned, the same single KEGG:04382 remained significant, and two terms came from microRNAs (dre-mir-206-3p, p = 0.099, one gene of the two in the set, plus the root node). Coverage of *D. rerio* in that database is effectively absent.

Table S9. Differentially expressed genes without official symbols

Label	Ensembl ID	log_2FC	Cluster
F1QEW2_DANRE	ENSDARG00000056248	−2.60	MCL 6
X1WG46_DANRE	ENSDARG00000031588	−4.30	MCL 6
ENSDARP00000131071	ENSDARG00000103199	−2.79	MCL 6
zgc:136930	ENSDARG00000055192	−2.46	MCL 3
—	ENSDARG00000113858	−7.84	—
—	ENSDARG00000114910	+0.43	—
—	ENSDARG00000110528	+0.48	—
—	ENSDARG00000117695	−0.72	—
—	ENSDARG00000110845	−1.08	—
—	ENSDARG00000117309	−3.75	—
—	ENSDARG00000106267	+2.36	—
—	ENSDARG00000117510	+1.77	—
—	ENSDARG00000117707	−1.19	—
—	ENSDARG00000116617	−2.48	—
—	ENSDARG00000097559	−7.04	—

Table S9. Genes without official symbols in the current annotation release. The first four belong to the interpreted clusters and are labelled in Table 8 of the main text and in Figure 8 by protein identifiers or clone names.

Table S10. Cluster robustness checks

Cluster	Initial	Without txt-only edges	Highest-degree node removed	Paralogue collapse	Status
MCL 1	9 nodes, 10 edges	6 edges, component of 6	*jun*: 5 edges, component of 5	8 nodes, 8 edges, connected	Robust
MCL 2	8, 17	17 edges, fully connected	*p3h1*: 11 edges, component of 6	6 nodes, 8 edges, connected	Robust
MCL 3	7, 8	7 edges, component of 6	*krt5*: 3 edges, component of 4	4 nodes, 3 edges	Reflects family
MCL 4	6, 10	4 edges, component of 4	*ifi46*: 5 edges, component of 5	5 nodes, 8 edges	Literature-driven
MCL 5	5, 5	5 edges, connected	*rrm2b*: 2 edges	unchanged	Robust
MCL 6	5, 7	4 edges, component of 5	*F1QEW2*: 3 edges	unchanged	Hypothesis

Table S10. Cluster robustness checks. Removal of the highest-degree node tests whether cluster density is driven by how well a single gene has been studied.

Table S11. Well-characterised targets of the glucocorticoid receptor

Gene	log_2FC (this study)	padj	log_2FC (published table)
ddit4	−0.76	3.7×10^{-5}	−0.89
cebpd	−1.36	0.056	−1.34
dusp1	−0.93	0.082	−0.80
tsc22d3 (GILZ)	−0.44	0.29	−0.42
per1a	−0.59	0.32	−0.55
klf9	−2.09	0.71	−2.21
fkbp5	−1.50	0.91	−1.54

Table S11. Receptor targets well characterised in the literature. The set was selected from the literature and is not independent; it is shown as an illustration accompanying the enrichment result. Only *ddit4* is individually significant.

Table S12. Genes significant both here and in Demin et al. (2020)

Gene	$\log_2$FC under 5-week CUS (Demin et al., 2020)	$\log_2$FC under 14-day CUS (this study)	padj (this study)
zgc:152791	−4.09	+2.15	0.23
ifi46 / si:dkey-188i13.7	−2.50	+1.46	0.040
CR318588.1	−3.87	Not detectable	—
draxin	+0.67	+0.91	0.13

Table S12. Three of the four genes are interferon-inducible and change in opposite directions. Of the 196 DEGs of the 5-week protocol, 169 are detectable in the present data; directional agreement is 70 of 169 (41%; binomial test $p = 0.031$); the correlation of effect-size estimates is Spearman $\rho = -0.25$ ($p = 9 \times 10^{-4}$).

Table S13. Boundaries of the blocks of unaligned reads

Run	Transition	Spot number	Meaning
SRR6410933	A → U	19,195,816	End of the aligned part of library 1
SRR6410933	U → A	27,540,278	Start of library 2
SRR6410933	A → U	45,928,444	End of the aligned part of library 2
SRR6410933	U → A	53,577,689	Start of library 3
SRR6410933	A → U	69,819,337	End of the aligned part of library 3
SRR6410932	A → U	20,827,327	End of the aligned part of library 1
SRR6410932	U → A	29,166,078	Start of library 2
SRR6410932	A → U	47,610,873	End of the aligned part of library 2
SRR6410932	U → A	55,378,561	Start of library 3
SRR6410932	A → U	70,484,832	End of the aligned part of library 3

Table S13. Block boundaries located by bisection. A, at least one read of the pair is aligned; U, both reads unaligned. Spot ranges for library extraction are given in Table 1 of the main text.

Table S14. Alignment metrics by library

Library	Read pairs	Uniquely, %	Multi-mapped, %	Unaligned, %	Junction-spanning reads, M	Annotated, %
C1	27,540,277	81.6	10.8	7.3	16.6	95
C2	26,037,411	81.4	11.0	7.3	15.0	95
C3	23,127,165	81.2	11.0	7.5	13.4	94
S1	29,166,077	80.6	11.6	7.5	15.6	94
S2	26,212,483	81.5	11.2	7.0	15.5	95
S3	21,535,391	81.5	11.1	7.1	13.1	95

Table S14. STAR alignment results against the GRCz11 assembly. For comparison, the deposited alignment (HISAT2, GRCz10, without splicing) reached an aligned fraction of 63.7 % in the control and 64.2 % under stress, with no junction-spanning reads placed. "Junction-spanning reads, M" is the number of reads aligned across an exon–exon junction, in millions, not the number of distinct junctions; of the distinct junctions detected, 94–95 % were annotated.

Table S15. Contribution of evidence sources to the main network

Evidence source	Edges with a non-zero partial score	Maximum partial score
Textmining	123	0.889
Coexpression	63	0.688
Experiments	56	0.558
Curated databases	39	0.916
Co-occurrence	18	0.403
Genomic neighbourhood	2	0.051
Gene fusion	1	0.002

Table S15. Distribution of the 137 edges of the main network by evidence source. One edge may carry partial scores from several sources.

Table S16. Recount of the differentially expressed genes

Category	Count
Published table (Supplement 2 of [1]), genes	24 035
Published DEGs at padj < 0.05 (recomputed)	155
published up / down	78 / 77
Genes with an adjusted p-value	20 863
DEGs at FDR < 0.05	275
up-regulated	139
down-regulated	136
Published DEGs receiving an adjusted p-value here	140
agreeing in direction	138
Genes with baseMean >= 10 in both analyses	15 690
published DEGs in that set	134
agreeing in direction in that set	132
reaching FDR < 0.05 here	101
DEGs not in the published list	174
Check: 101 + 174	275

Table S16. Categories of differentially expressed gene recomputed from the data. The published counts are recomputed from Supplement 2 of [1] at the same threshold. The last three rows restrict both analyses to genes with a base mean of at least ten, the filter under which the comparison reproduces exactly.

Table S17. Per-library counts for the questioned genes and twelve marker panels

Panel	Symbol	Gene	Status	Norm ctrl1	Raw ctrl1	Norm ctrl2	Raw ctrl2	Norm ctrl3	Raw ctrl3	Norm str1	Raw str1	Norm str2	Raw str2	Norm str3	Raw str3
reviewer-listed	krt4	ENSDARG00000017624	DEG	50.60	55	363.4	365	97.40	90	10.10	11	19.90	21	11.50	10
reviewer-listed	krt5	ENSDARG00000058371	DEG	551.5	600	1 800	1 808	1 052	972	128.2	139	194.4	205	25.40	22
reviewer-listed	krt17	ENSDARG00000094041	DEG	2.80	3	24.90	25	20.60	19	0.900	1	1.90	2	0	0
reviewer-listed	col17a1a	ENSDARG00000069415	DEG	9.20	10	4	4	29.20	27	0	0	0	0	0	0
reviewer-listed	col17a1b	ENSDARG00000079011	DEG	23	25	138.4	139	31.40	29	11.10	12	5.70	6	12.70	11
reviewer-listed	pkp1b	ENSDARG00000052705	DEG	36.80	40	70.70	71	55.20	51	16.60	18	5.70	6	2.30	2
reviewer-listed	cldna	ENSDARG00000069888	DEG	52.40	57	115.5	116	58.40	54	29.50	32	26.60	28	23	20
reviewer-listed	cldnb	ENSDARG00000009544	DEG	18.40	20	95.60	96	22.70	21	6.50	7	2.80	3	1.20	1
reviewer-listed	icn	ENSDARG00000009978	DEG	286.8	312	535.6	538	358.1	331	154	167	222.9	235	144.1	125
reviewer-listed	icn2	ENSDARG00000055514	DEG	10.10	11	50.80	51	19.50	18	3.70	4	1.90	2	0	0
reviewer-listed	upk1a	ENSDARG00000021866	DEG	5.50	6	24.90	25	13	12	0.900	1	0	0	0	0
reviewer-listed	ela2l	ENSDARG00000056765	DEG	0	0	0	0	0	0	52.60	57	37.90	40	83	72
reviewer-listed	ampd1	ENSDARG00000033832	DEG	3.70	4	0	0	2.20	2	31.40	34	9.50	10	38	33
neuron	snap25a	ENSDARG00000020609	not a DEG	24 723	26 896	27 801	27 923	25 988	24 019	29 547	32 031	26 771	28 225	24 379	21 154
neuron	snap25b	ENSDARG00000058117	not a DEG	8 546	9 297	8 236	8 272	7 662	7 082	7 924	8 590	8 144	8 586	7 933	6 884
neuron	elavl3	ENSDARG00000014420	not a DEG	15 718	17 099	14 800	14 865	15 646	14 461	16 424	17 805	13 964	14 722	15 330	13 302
neuron	syt1a	ENSDARG00000030614	not a DEG	41 897	45 579	43 324	43 514	40 841	37 747	41 813	45 328	39 698	41 855	39 309	34 109
neuron	nefma	ENSDARG00000021351	not a DEG	406.3	442	346.5	348	533.4	493	476.9	517	458.1	483	372.2	323
neuron	nefmb	ENSDARG00000043697	not a DEG	10.10	11	17.90	18	17.30	16	13.80	15	12.30	13	10.40	9
neuron	tubb5	ENSDARG00000037997	not a DEG	4 613	5 018	4 307	4 326	4 352	4 022	4 950	5 366	5 261	5 547	4 735	4 109
neuron	gap43	ENSDARG00000099744	not a DEG	4 947	5 382	4 898	4 920	5 208	4 813	5 460	5 919	4 849	5 112	4 611	4 001
neuron	sncb	ENSDARG00000104945	not a DEG	9 812	10 674	9 689	9 732	8 941	8 264	10 516	11 400	10 030	10 575	9 562	8 297
neuron	stmn1b	ENSDARG00000033655	not a DEG	12 281	13 360	13 187	13 245	13 915	12 861	15 168	16 443	12 607	13 292	12 474	10 824
neuron	syn2a	ENSDARG00000045945	not a DEG	6 028	6 558	5 554	5 578	5 788	5 350	5 787	6 274	6 185	6 521	5 726	4 969
neuron	rbfox3a	ENSDARG00000010083	not a DEG	563.5	613	702.9	706	661.1	611	621.7	674	570	601	632.7	549
radial_glia	gfap	ENSDARG00000025301	not a DEG	748.2	814	579.5	582	739	683	813.6	882	728.4	768	805.6	699
radial_glia	s100b	ENSDARG00000057598	not a DEG	3 399	3 698	2 324	2 334	3 151	2 912	3 968	4 302	3 619	3 816	3 164	2 745
radial_glia	fabp7a	ENSDARG00000007697	not a DEG	8 187	8 907	6 034	6 061	6 510	6 017	6 824	7 398	7 484	7 890	8 155	7 076
radial_glia	slc1a2b	ENSDARG00000102453	not a DEG	39 951	43 462	27 928	28 051	31 701	29 300	39 046	42 329	34 438	36 309	45 401	39 395
radial_glia	glula	ENSDARG00000099776	not a DEG	17 633	19 183	12 704	12 760	13 430	12 413	15 639	16 954	14 263	15 038	16 634	14 434
radial_glia	her4.1	ENSDARG00000056732	not a DEG	124.1	135	119.5	120	141.7	131	242.6	263	163.1	172	187.8	163
radial_glia	her4.5	ENSDARG00000056729	DEG	8.30	9	4	4	6.50	6	59	64	19	20	49.60	43
radial_glia	gja1a	ENSDARG00000071192	not a DEG	11.90	13	15.90	16	11.90	11	21.20	23	11.40	12	17.30	15
radial_glia	cx43	not found	absent	—	—	—	—	—	—	—	—	—	—	—	—
radial_glia	slc1a3a	ENSDARG00000104431	not a DEG	114.9	125	142.4	143	138.5	128	130.1	141	171.7	181	160.2	139
oligodendrocyte	mbpa	ENSDARG00000036186	not a DEG	3 916	4 260	3 800	3 817	4 824	4 459	3 488	3 781	3 476	3 665	2 296	1 992
oligodendrocyte	plp1a	ENSDARG00000103732	not a DEG	49.60	54	49.80	50	71.40	66	69.20	75	33.20	35	26.50	23
oligodendrocyte	plp1b	ENSDARG00000011929	DEG	1 132	1 231	1 181	1 186	1 284	1 187	905.8	982	924.8	975	697.2	605
oligodendrocyte	olig1	ENSDARG00000040948	not a DEG	211.4	230	230	231	201.2	186	214.9	233	239	252	190.2	165
oligodendrocyte	olig2	ENSDARG00000040946	not a DEG	341	371	389.3	391	363.5	336	427.1	463	303.5	320	318.1	276
oligodendrocyte	mpz	ENSDARG00000038609	DEG	382.4	416	358.4	360	404.7	374	239.8	260	310.2	327	214.4	186
oligodendrocyte	sox10	ENSDARG00000077467	not a DEG	215.1	234	185.2	186	288.9	267	206.6	224	230.5	243	147.5	128
microglia	mpeg1.1	ENSDARG00000055290	not a DEG	9.20	10	18.90	19	14.10	13	11.10	12	8.50	9	17.30	15
microglia	csf1ra	ENSDARG00000102986	not a DEG	198.5	216	218	219	221.8	205	241.7	262	203	214	306.6	266
microglia	p2ry12	ENSDARG00000069945	not a DEG	11	12	6	6	11.90	11	8.30	9	16.10	17	12.70	11
microglia	apoeb	ENSDARG00000040295	not a DEG	1 619	1 761	1 430	1 436	1 429	1 321	1 730	1 876	2 063	2 175	1 724	1 496
microglia	lcp1	ENSDARG00000023188	not a DEG	193	210	228	229	166.6	154	199.2	216	173.6	183	247.8	215
microglia	mfap4	not found	absent	—	—	—	—	—	—	—	—	—	—	—	—
epidermis	krt91	ENSDARG00000036830	not a DEG	672.9	732	2 177	2 187	1 172	1 083	200.2	217	391.7	413	48.40	42
epidermis	krt92	ENSDARG00000036834	not a DEG	0.900	1	0	0	0	0	0	0	1.90	2	0	0
epidermis	krt97	ENSDARG00000000212	not a DEG	241.8	263	487.9	490	422	390	60.90	66	43.60	46	1.20	1
epidermis	krt8	ENSDARG00000058358	not a DEG	984.5	1 071	1 311	1 317	1 008	932	1 141	1 237	987.4	1 041	1 217	1 056
epidermis	tp63	ENSDARG00000044356	not a DEG	14.70	16	74.70	75	32.50	30	18.40	20	13.30	14	12.70	11
epidermis	cldni	ENSDARG00000054616	not a DEG	0.900	1	11.90	12	4.30	4	1.80	2	2.80	3	2.30	2
epidermis	krtt1c19e	ENSDARG00000090268	not a DEG	0	0	1	1	1.10	1	0	0	0.900	1	0	0
epidermis	anxa1a	ENSDARG00000026726	not a DEG	73.50	80	184.2	185	109.3	101	61.80	67	97.70	103	51.90	45
epidermis	s100a10b	ENSDARG00000025254	not a DEG	143.4	156	253.9	255	166.6	154	166	180	217.2	229	145.2	126
epidermis	cyt1	ENSDARG00000092947	not a DEG	1.80	2	2	2	0	0	0.900	1	1.90	2	0	0
epidermis	cyt1l	ENSDARG00000036832	not a DEG	0	0	0	0	0	0	0	0	0	0	0	0
epidermis	krt1-19d	ENSDARG00000023082	not a DEG	11	12	56.80	57	14.10	13	11.10	12	5.70	6	4.60	4
epidermis	epcam	ENSDARG00000040534	not a DEG	7.40	8	87.60	88	17.30	16	0	0	3.80	4	0	0

epidermis	agr2	ENSDARG00000070480	not a DEG	3.70	4	7	7	7.60	7	0	0	2.80	3	0	0
epidermis	muc5.1	ENSDARG00000070331	not a DEG	4.60	5	29.90	30	33.50	31	9.20	10	1.90	2	1.20	1
epidermis	muc5.2	ENSDARG00000058556	DEG	9.20	10	45.80	46	60.60	56	8.30	9	1.90	2	1.20	1
olfactory_epithelium	omp	not found	absent	—	—	—	—	—	—	—	—	—	—	—	—
olfactory_epithelium	ora1	ENSDARG00000098601	not a DEG	0	0	0	0	0	0	0	0	0	0	0	0
olfactory_epithelium	ora2	not found	absent	—	—	—	—	—	—	—	—	—	—	—	—
olfactory_epithelium	ora3	not found	absent	—	—	—	—	—	—	—	—	—	—	—	—
olfactory_epithelium	ora4	ENSDARG00000078223	not a DEG	0	0	0	0	0	0	0	0	0	0	0	0
olfactory_epithelium	ora5	ENSDARG00000078257	not a DEG	0	0	0	0	0	0	0	0	0.900	1	0	0
olfactory_epithelium	ora6	not found	absent	—	—	—	—	—	—	—	—	—	—	—	—
olfactory_epithelium	trpc2b	ENSDARG00000003344	not a DEG	0	0	2	2	3.20	3	0.900	1	0	0	0	0
olfactory_epithelium	s100z	ENSDARG00000038729	not a DEG	54.20	59	71.70	72	60.60	56	57.20	62	51.20	54	54.20	47
olfactory_epithelium	gng13a	ENSDARG00000024740	not a DEG	0	0	0	0	0	0	0	0	0	0	0	0
olfactory_epithelium	gng13b	ENSDARG00000037921	not a DEG	2 169	2 360	2 596	2 607	1 655	1 530	2 093	2 269	2 018	2 128	2 036	1 767
olfactory_epithelium	calb2b	ENSDARG00000036344	not a DEG	14.70	16	26.90	27	17.30	16	11.10	12	17.10	18	20.70	18
olfactory_epithelium	cnga2	not found	absent	—	—	—	—	—	—	—	—	—	—	—	—
exocrine_pancreas	ela2	ENSDARG00000056744	not a DEG	3.70	4	2	2	2.20	2	0	0	0	0	0	0
exocrine_pancreas	prss1	ENSDARG00000042993	not a DEG	23	25	14.90	15	11.90	11	16.60	18	14.20	15	12.70	11
exocrine_pancreas	ctrb1	not found	absent	—	—	—	—	—	—	—	—	—	—	—	—
exocrine_pancreas	cela1.1	ENSDARG00000043175	not a DEG	0	0	0	0	0	0	0	0	0	0	0	0
exocrine_pancreas	cpa5	ENSDARG00000021339	not a DEG	0.900	1	1	1	3.20	3	2.80	3	2.80	3	2.30	2
exocrine_pancreas	try	not found	absent	—	—	—	—	—	—	—	—	—	—	—	—
exocrine_pancreas	amy2a	ENSDARG00000013856	not a DEG	0	0	1	1	0	0	0.900	1	0	0	0	0
exocrine_pancreas	cel.1	ENSDARG00000017490	not a DEG	0	0	0	0	1.10	1	0	0	0	0	0	0
exocrine_pancreas	ctrl	ENSDARG00000068680	not a DEG	0.900	1	3	3	0	0	0.900	1	0	0	2.30	2
erythrocyte	hbaa1	ENSDARG00000097011	not a DEG	3 617	3 935	4 686	4 707	7 036	6 503	4 045	4 385	3 636	3 833	3 125	2 712
erythrocyte	hbba1	ENSDARG00000089087	DEG	2 865	3 117	3 577	3 593	4 448	4 111	2 348	2 545	2 722	2 870	2 604	2 260
erythrocyte	hbae1.1	ENSDARG00000089475	not a DEG	0	0	0	0	0	0	0	0	0	0	0	0
erythrocyte	alas2	ENSDARG00000038643	not a DEG	162.7	177	209.1	210	221.8	205	167	181	158.4	167	92.20	80
erythrocyte	ba1	not found	absent	—	—	—	—	—	—	—	—	—	—	—	—
erythrocyte	hbae3	ENSDARG00000079305	not a DEG	0	0	0	0	0	0	0	0	0	0	0	0
endothelium	kdrl	ENSDARG00000105215	not a DEG	890.7	969	1 175	1 180	1 050	970	999	1 083	807.2	851	805.6	699
endothelium	cdh5	ENSDARG00000075549	not a DEG	285.9	311	396.3	398	308.4	285	522.1	566	435.4	459	425.3	369
endothelium	fli1a	not found	absent	—	—	—	—	—	—	—	—	—	—	—	—
endothelium	tie1	ENSDARG00000004105	not a DEG	93.80	102	172.2	173	121.2	112	91.30	99	109.1	115	111.8	97
endothelium	flt1	ENSDARG00000019371	not a DEG	286.8	312	364.4	366	364.6	337	318.2	345	281.7	297	238.6	207
endothelium	pecam1	not found	absent	—	—	—	—	—	—	—	—	—	—	—	—
pineal	aanat2	ENSDARG00000079802	not a DEG	50.60	55	15.90	16	21.60	20	30.40	33	40.80	43	26.50	23
pineal	exorh	ENSDARG00000111860	not a DEG	2 186	2 378	208.1	209	399.2	369	119	129	358.5	378	133.7	116
pineal	otx5	ENSDARG00000043483	not a DEG	81.80	89	11	11	5.40	5	33.20	36	22.80	24	20.70	18
pineal	rorb	ENSDARG00000033498	not a DEG	103	112	68.70	69	79	73	90.40	98	84.40	89	80.70	70
pineal	asmt	ENSDARG00000098249	not a DEG	572.7	623	23.90	24	71.40	66	10.10	11	55	58	10.40	9
skeletal_muscle	myhz1.1	ENSDARG00000067990	not a DEG	6.40	7	5	5	11.90	11	12.90	14	6.60	7	9.20	8
skeletal_muscle	acta1a	ENSDARG00000036371	not a DEG	19.30	21	30.90	31	6.50	6	20.30	22	11.40	12	13.80	12
skeletal_muscle	mylpfa	ENSDARG00000053254	not a DEG	3.70	4	9	9	3.20	3	0	0	2.80	3	0	0
skeletal_muscle	ckma	ENSDARG00000035327	not a DEG	7.40	8	24.90	25	14.10	13	14.80	16	14.20	15	8.10	7
skeletal_muscle	tnnt3a	ENSDARG00000030270	not a DEG	0	0	0	0	0	0	0	0	0	0	0	0
skeletal_muscle	mybpc1	ENSDARG00000045560	not a DEG	38.60	42	54.80	55	23.80	22	24.90	27	23.70	25	23	20
skeletal_muscle	desma	ENSDARG00000058656	not a DEG	44.10	48	24.90	25	36.80	34	31.40	34	32.20	34	31.10	27
cartilage_bone	col2a1a	ENSDARG00000069093	not a DEG	34	37	50.80	51	48.70	45	52.60	57	70.20	74	50.70	44
cartilage_bone	col10a1a	ENSDARG00000054753	not a DEG	8.30	9	8	8	5.40	5	1.80	2	70.20	74	1.20	1
cartilage_bone	sp7	ENSDARG00000019516	not a DEG	0.900	1	1	1	2.20	2	2.80	3	6.60	7	0	0
cartilage_bone	bglap	ENSDARG00000058414	not a DEG	1.80	2	0	0	0	0	0	0	0	0	0	0
cartilage_bone	col1a1a	ENSDARG00000012405	not a DEG	68.90	75	136.4	137	384.1	355	43.40	47	308.3	325	71.50	62
cartilage_bone	runx2b	ENSDARG00000059233	not a DEG	36.80	40	58.70	59	42.20	39	61.80	67	43.60	46	56.50	49
lens_eye	cryaa	ENSDARG00000053502	not a DEG	0	0	0	0	0	0	0	0	0	0	0	0
lens_eye	crybb1	ENSDARG00000068507	not a DEG	0	0	0	0	1.10	1	0.900	1	0	0	0	0
lens_eye	rho	ENSDARG00000002193	not a DEG	57	62	1	1	0	0	0	0	405.9	428	2.30	2
lens_eye	gnat1	ENSDARG00000044199	not a DEG	711.5	774	23.90	24	74.70	69	3.70	4	117.6	124	8.10	7
lens_eye	crygm2d1	ENSDARG00000087324	not a DEG	0	0	0	0	0	0	0	0	0	0	0	0
lens_eye	opn1sw1	ENSDARG00000045677	not a DEG	0	0	1	1	0	0	0.900	1	19.90	21	1.20	1

Table S17. Normalised and raw counts in each of the six libraries for the genes raised by the reviewer and for twelve tissue marker panels. Normalised values are median-of-ratios counts. Genes absent from the count matrix are marked as such.

Table S18. Genes reassigned as of probable non-neural origin

Gene	Sym	Reason	Category	Tissue	Evidence	Log2Fold Change	Adj. p
ENSDARG00000017624	krt4	non-neural annotation	epithelial	skin, both epidermal layers	COKUS19	-3.62	0.0063
ENSDARG00000058371	krt5	non-neural annotation	epithelial	skin, both epidermal layers	COKUS19	-3.29	0.017
ENSDARG00000094041	krt17	non-neural annotation	epithelial	periderm-preferred	COKUS19	-4.03	0.047
ENSDARG00000069415	col17a1a	non-neural annotation	epithelial	skin	KIM10	-6.26	0.00437
ENSDARG00000052705	pkp1b	non-neural annotation	epithelial	desmosome, epidermis	FAMILY	-2.69	0.00017
ENSDARG00000069888	cldna	non-neural annotation	epithelial	periderm / epithelial tight junction	FAMILY	-1.51	0.00831
ENSDARG00000009544	cldnb	non-neural annotation	epithelial	periderm / lateral line	FAMILY	-3.67	0.00031
ENSDARG00000009978	icn	non-neural annotation	epithelial	epidermis; ictacalcin, an S100-family protein	HSIAO03	-1.18	8.50×10^{-5}
ENSDARG00000055514	icn2	non-neural annotation	epithelial	epidermis, by the paralogue icn	PARALOGUE	-3.78	0.00421
ENSDARG00000021866	upk1a	non-neural annotation	epithelial	urinary tract epithelium (cloaca, pronephric duct)	FAMILY	-5.33	0.021
ENSDARG00000102750	cdh1	non-neural annotation	epithelial	epithelial E-cadherin, both skin layers	COKUS19	-2.50	0.042
ENSDARG00000058556	muc5.2	non-neural annotation	epithelial	mucous secretory epithelium	FAMILY	-3.31	0.00313
ENSDARG00000044655	st14b	non-neural annotation	epithelial	epithelial matriptase	FAMILY	-3.65	0.033
ENSDARG00000103917	znf185	non-neural annotation	epithelial	epithelial, actin cytoskeleton	FAMILY	-1.74	0.037
ENSDARG00000056765	ela2l	non-neural annotation	organ	exocrine pancreas	ORGAN	8.30	3.72×10^{-8}
ENSDARG00000033832	ampd1	non-neural annotation	organ	striated muscle isoform	ORGAN	3.73	0.00318
ENSDARG00000052708	tnni1b	non-neural annotation	organ	striated muscle troponin	ORGAN	1.23	0.019
ENSDARG00000079393	tmprss15	non-neural annotation	organ	intestinal enteropeptidase	ORGAN	-1.44	2.75×10^{-12}
ENSDARG00000044280	opn1mw2	non-neural annotation	organ	retinal cone opsin	ORGAN	5.77	0.047
ENSDARG00000042850	nphs2	non-neural annotation	organ	kidney podocyte	ORGAN	-4.12	0.037
ENSDARG00000096599	si:ch211-160d14.9	presence/absence	structural	all-or-none across pools	counts in this dataset	-8.50	6.56×10^{-9}
ENSDARG00000113858	ENSDARG00000113858	presence/absence	structural	all-or-none across pools	counts in this dataset	-7.84	3.16×10^{-7}
ENSDARG00000097247	ENSDARG00000097247	presence/absence	structural	all-or-none across pools	counts in this dataset	7.17	3.50×10^{-5}
ENSDARG00000110845	ENSDARG00000110845	presence/absence	structural	all-or-none across pools	counts in this dataset	6.16	0.00313
ENSDARG00000100771	ENSDARG00000100771	presence/absence	structural	all-or-none across pools	counts in this dataset	-5.98	0.00551
ENSDARG00000083597	dre-mir-22a	presence/absence	structural	all-or-none across pools	counts in this dataset	5.85	0.00801
ENSDARG00000076264	zgc:195170	presence/absence	structural	all-or-none across pools	counts in this dataset	5.73	0.016
ENSDARG00000092025	si:dkey-121n8.8	presence/absence	structural	all-or-none across pools	counts in this dataset	5.55	0.028

Table S18. The 28 genes set aside in Section 2.11, with the category, the tissue of reported expression, the evidence code and the effect size. The evidence codes are: COKUS19, the tissue-specific transcriptomes of the two skin epithelial layers of zebrafish embryos [27]; KIM10, the expression profiles of zebrafish type XVII collagen [29]; HSIAO03, the skin-specific expression of ictacalcin during zebrafish embryogenesis [28]; FAMILY, the tissue specificity of the gene family to which the gene belongs, without a zebrafish expression record of its own; ORGAN, annotation to an organ-restricted paralogue; PARALOGUE, the assignment of the closest paralogue; and "counts in this dataset" for the eight genes set aside on the presence/absence pattern alone. The evidence tier grades the zebrafish expression record itself, as defined in Section 4.7: A, recorded in the attributed tissue in adult fish with no neural record; B, recorded in that tissue at embryonic or larval stages or not resolved to a stage; C, a record that also includes adult brain or nervous system, so that it does not discriminate the origin of the transcript; D, no zebrafish expression annotation at all; E, the count pattern, with no expression evidence. Three entries of the submitted version are corrected here: icn and icn2 encode ictacalcins, calcium-binding proteins of the S100 family, and not antimicrobial peptides; [28] annotates icn and not icn2, whose assignment follows its paralogue; and the zebrafish record for upk1a places it in the urinary tract rather than in the epidermis. Table S19d reports the exclusion repeated for the nested tiers.

Table S19a. Gene-set tests before and after the reassignment

Set	Analysis	N	Direction	T	P
GR_INDUCED_EACHUS	all genes	2 443	Down	-3.18	0.00146
GR_INDUCED_EACHUS	non-neural excluded	2 442	Down	-3.22	0.00128
GR_REPRESSED_EACHUS	all genes	1 951	Up	3.17	0.00154
GR_REPRESSED_EACHUS	non-neural excluded	1 948	Up	3.28	0.00105
DEMIN2020_CUS_UP	all genes	62	Down	-0.606	0.545
DEMIN2020_CUS_UP	non-neural excluded	62	Down	-0.623	0.533
DEMIN2020_CUS_DOWN	all genes	129	Up	3.18	0.0015
DEMIN2020_CUS_DOWN	non-neural excluded	129	Up	3.20	0.0014
ABETA42_UP	all genes	2 471	Up	1.28	0.200
ABETA42_UP	non-neural excluded	2 469	Up	1.28	0.202
ABETA42_DOWN	all genes	2 835	Down	-0.669	0.503
ABETA42_DOWN	non-neural excluded	2 831	Down	-0.636	0.525

Table S19a. Competitive tests of the glucocorticoid-dependent sets and of the marker panels on the full list and after removal of the twenty-eight reassigned genes.

Table S19b. Gene-set tests across models

Model	Contrast	Set	N	Direction	T	P	N DEG	How it may be read
~ group	group	GR-induced	2 443	Down	-3.195	0.0014	271	unadjusted; the analysis reported in the paper
~ group	group	GR-repressed	1 951	Up	3.228	0.00125	271	unadjusted; the analysis reported in the paper
~ group, 28 genes set aside	group	GR-induced	2 442	Down	-3.221	0.00128	—	tests gene-level annotation artefact, not sample composition
~ group, 28 genes set aside	group	GR-repressed	1 948	Up	3.278	0.00105	—	tests gene-level annotation artefact, not sample composition
~ group, 85 genes set aside	group	GR-induced	2 433	Down	-3.36	0.00078	—	annotation and genomic-block artefact together
~ group, 85 genes set aside	group	GR-repressed	1 937	Up	3.201	0.00137	—	annotation and genomic-block artefact together
~ group + epidermal score (as measured)	group	GR-induced	2 443	Down	-0.919	0.3581	27	collinear with group (r = -0.82); not interpretable
~ group + epidermal score (as measured)	group	GR-repressed	1 951	Up	1.613	0.1067	27	collinear with group (r = -0.82); not interpretable
~ group + epidermal score (as measured)	epidermal score	GR-induced	2 443	Up	1.619	0.1054	16	collinear with group; not interpretable
~ group + epidermal score (as measured)	epidermal score	GR-repressed	1 951	Down	-0.816	0.4144	16	collinear with group; not interpretable
~ group + epidermal score centred within groups	group	GR-induced	2 443	Down	-3.082	0.00206	246	covariate orthogonal by construction; group contrast retains power
~ group + epidermal score centred within groups	group	GR-repressed	1 951	Up	3.152	0.00162	246	covariate orthogonal by construction; group contrast retains power
~ group + epidermal score centred within groups	epidermal score, within groups	GR-induced	2 443	Up	1.619	0.1054	16	within-group composition only; silent on the between-group difference
~ group + epidermal score centred within groups	epidermal score, within groups	GR-repressed	1 951	Down	-0.816	0.4144	16	within-group composition only; silent on the between-group difference

Table S19b. The two glucocorticoid sets under every model considered in Section 2.11, with the reading each model admits. The epidermal score and the group factor are collinear in these libraries (r = −0.82; per-library scores 5.97, 7.71, 6.82 in the control pools and 5.37, 5.42, 4.14 in the stress pools), so the model containing the score as measured is not identifiable and its p-values are reported for completeness only: random covariates of the same collinearity remove the glucocorticoid signal in 14 of 31 runs and reduce the gene count to a median of 84 (Table S19c). Centring the score within groups removes the collinearity by construction but restricts the covariate to within-group variation, so that model is silent on the difference between the group means, which is 1.86 $\log_2$ units against a within-group spread of 1.74 and 1.28. No model in this table separates a difference in tissue composition from the treatment.

Table S19c. Random covariates of equal collinearity

Seed	Rep	R with group	N DEG	P ind	Dir ind	P rep	Dir rep
11	1	-0.839	41	0.00141	Down	0.00278	Up
11	2	-0.800	61	4.09×10^{-8}	Down	6.58×10^{-5}	Up

11	3	0.846	133	0.002	Down	0.013	Up
11	4	-0.799	90	7.62×10^{-8}	Down	7.04×10^{-5}	Up
11	5	0.840	84	0.036	Down	0.080	Up
12	1	0.789	99	0.341	Down	0.114	Up
12	2	0.846	118	5.56×10^{-5}	Down	0.00635	Up
12	3	-0.815	74	0.00154	Down	0.00141	Up
12	4	0.821	74	0.177	Down	0.084	Up
12	5	-0.814	97	0.059	Down	0.058	Up
13	1	0.811	88	0.431	Up	0.528	Up
13	2	-0.832	17	0.282	Up	0.805	Up
13	3	-0.844	45	0.030	Up	0.911	Down
13	4	-0.788	55	0.428	Down	0.188	Up
13	5	0.788	80	4.67×10^{-5}	Down	0.00488	Up
14	1	0.808	58	3.10×10^{-7}	Down	0.00025	Up
14	2	0.824	68	7.04×10^{-7}	Down	0.00064	Up
14	3	0.809	97	0.882	Down	0.165	Up
14	4	0.827	148	0.063	Down	0.057	Up
14	5	-0.821	99	0.033	Down	0.021	Up
15	1	0.810	76	0.00114	Down	0.014	Up
21	1	-0.812	194	1.09×10^{-8}	Down	0.00016	Up
21	2	-0.802	81	0.205	Down	0.032	Up
21	3	0.833	39	0.023	Up	0.783	Up
21	4	-0.832	145	2.86×10^{-10}	Down	5.44×10^{-5}	Up
21	5	-0.812	59	0.514	Down	0.166	Up
22	1	0.837	85	0.072	Down	0.089	Up
22	2	0.822	106	4.24×10^{-11}	Down	3.38×10^{-5}	Up
22	3	0.828	88	1.04×10^{-7}	Down	0.0005	Up
22	4	0.839	49	1.14×10^{-5}	Down	0.00532	Up
22	5	0.805	103	1.17×10^{-5}	Down	0.00067	Up

Table S19c. Thirty-one random covariates constructed to have the same collinearity with the group factor as the measured epidermal score. For each, the number of DEGs and whether the glucocorticoid signal is retained.

Table S19d. Sensitivity of the exclusion to the strength of the evidence

Genes set aside	N	GR-induced	P induced	GR-repressed	P repressed	GO terms at FDR < 0.05	Terms retained
none (all 275)	0	Down	0.00146	Up	0.00154	16	collagen trimer; intermediate filament; intermediate filament cytoskeleton; basement membrane
adult zebrafish expression (tier A)	3	Down	0.0015	Up	0.00155	13	collagen trimer; basement membrane; regulation of cell differentiation; regulation of neurogenesis
+ zebrafish, stage not adult or not resolved (tier B)	14	Down	0.0014	Up	0.00152	6	collagen trimer; regulation of cell differentiation; regulation of neurogenesis; regulation of cell development
+ record includes adult neural tissue (tier C)	18	Down	0.00136	Up	0.00153	6	collagen trimer; regulation of cell differentiation; regulation of neurogenesis; regulation of cell development
+ no zebrafish expression data (tier D)	20	Down	0.00132	Up	0.00153	5	collagen trimer; regulation of cell differentiation; regulation of neurogenesis; regulation of cell development
all 28 set aside	28	Down	0.00128	Up	0.00105	5	collagen trimer; regulation of cell differentiation; regulation of neurogenesis; regulation of cell development

Table S19d. The glucocorticoid tests and the functional enrichment after setting aside nested subsets of the twenty-eight genes, ordered by the strength of the zebrafish expression evidence behind each assignment (tiers A to D of Table S18; the last row adds the eight genes set aside on the count pattern, tier E). The glucocorticoid tests are unaffected throughout. The epithelial terms fall as the grading predicts: three of the sixteen with the adult-supported genes, and the intermediate-filament, keratinocyte-differentiation and basement-membrane terms once tier B is added. None of this decides the origin of the transcripts, which is why they are set aside rather than attributed: no claim is made in either direction. Complement activation falls below three members when *st14b* is set aside at tier D.

Table S20. Enrichment before and after the reassignment

Analysis	Term	Name	K	Set size	P	Fdr
all 275	GO:0005581	collagen trimer	7	56	8.56×10^{-6}	0.0025
all 275	GO:0005882	intermediate filament	6	57	0.0001	0.012
all 275	GO:0045111	intermediate filament cytoskeleton	6	59	0.00012	0.012

all 275	GO:0005604	basement membrane	5	43	0.00025	0.018
all 275	GO:0045595	regulation of cell differentiation	9	163	0.00032	0.019
all 275	GO:0050767	regulation of neurogenesis	7	109	0.00061	0.026
all 275	GO:0030216	keratinocyte differentiation	3	14	0.00074	0.026
all 275	GO:0045109	intermediate filament organization	3	14	0.00074	0.026
all 275	GO:0060284	regulation of cell development	7	114	0.00079	0.026
all 275	GO:0006956	complement activation	3	15	0.00092	0.027
all 275	GO:0051960	regulation of nervous system development	7	121	0.00113	0.030
all 275	GO:0030020	extracellular matrix structural constituent conferring tensile strength	4	41	0.00204	0.043
all 275	GO:0048935	peripheral nervous system neuron development	3	20	0.00219	0.043
all 275	GO:0048934	peripheral nervous system neuron differentiation	3	20	0.00219	0.043
all 275	GO:0002252	immune effector process	4	42	0.00223	0.043
all 275	GO:0008544	epidermis development	4	43	0.00243	0.044
all 275	GO:0008395	steroid hydroxylase activity	3	23	0.0033	0.055
all 275	GO:0042552	myelination	4	48	0.00364	0.055
all 275	GO:0009913	epidermal cell differentiation	3	24	0.00374	0.055
all 275	GO:0008366	axon ensheathment	4	49	0.00393	0.055
all 275	GO:0007272	ensheathment of neurons	4	49	0.00393	0.055
all 275	GO:0005201	extracellular matrix structural constituent	4	50	0.00423	0.055
all 275	GO:0014003	oligodendrocyte development	3	26	0.00471	0.055
all 275	GO:0045104	intermediate filament cytoskeleton organization	3	26	0.00471	0.055
all 275	GO:0045103	intermediate filament-based process	3	26	0.00471	0.055
all 275	GO:0005576	extracellular region	19	747	0.00498	0.055
all 275	GO:0031012	extracellular matrix	7	159	0.00523	0.055
all 275	GO:0030312	external encapsulating structure	7	159	0.00523	0.055
all 275	GO:0006959	humoral immune response	3	28	0.00582	0.057
all 275	GO:0043588	skin development	3	28	0.00582	0.057
all 275	GO:0015291	secondary active transmembrane transporter activity	6	125	0.00631	0.059
all 275	GO:0022804	active transmembrane transporter activity	6	127	0.00681	0.062
all 275	GO:0099080	supramolecular complex	14	505	0.00742	0.066
all 275	GO:0007155	cell adhesion	13	456	0.00779	0.067
all 275	GO:0007043	cell-cell junction assembly	4	62	0.00906	0.075
all 275	GO:0005507	copper ion binding	3	33	0.00923	0.075
all 275	GO:0005198	structural molecule activity	7	180	0.010	0.079
all 275	GO:0032922	circadian regulation of gene expression	3	36	0.012	0.090
all 275	GO:0015293	symporter activity	4	68	0.012	0.092
all 275	GO:0050793	regulation of developmental process	10	330	0.013	0.092
all 275	GO:0050778	positive regulation of immune response	4	71	0.014	0.095
all 275	GO:2000026	regulation of multicellular organismal development	9	288	0.015	0.095
all 275	GO:0071560	cellular response to transforming growth factor beta stimulus	3	39	0.015	0.095
all 275	GO:0071559	response to transforming growth factor beta	3	39	0.015	0.095
all 275	GO:0007179	transforming growth factor beta receptor signaling pathway	3	39	0.015	0.095
all 275	GO:0000381	regulation of alternative mRNA splicing, via spliceosome	3	41	0.017	0.106
all 275	GO:0015804	neutral amino acid transport	3	42	0.018	0.111
all 275	GO:0048925	lateral line system development	5	116	0.019	0.113
all 275	GO:0048709	oligodendrocyte differentiation	3	43	0.019	0.113
all 275	GO:0007156	homophilic cell-cell adhesion	5	117	0.019	0.114
all 275	GO:0141091	transforming growth factor beta receptor superfamily signaling pathway	4	80	0.021	0.122
all 275	GO:0010001	glial cell differentiation	5	123	0.024	0.132
all 275	GO:0007178	cell surface receptor protein serine/threonine kinase signaling pathway	4	85	0.026	0.142
all 275	GO:0050776	regulation of immune response	4	86	0.027	0.142
all 275	GO:0016705	oxidoreductase activity, acting on paired donors, with incorporation or reduction of molecular oxygen	6	174	0.028	0.142
all 275	GO:0006915	apoptotic process	7	222	0.028	0.142
all 275	GO:0099081	supramolecular polymer	11	431	0.028	0.142
all 275	GO:0099512	supramolecular fiber	11	431	0.028	0.142
all 275	GO:0048584	positive regulation of response to stimulus	4	88	0.029	0.142
all 275	GO:0021782	glial cell development	4	88	0.029	0.142
all 275	GO:0099513	polymeric cytoskeletal fiber	9	330	0.032	0.147

all 275	GO:0042063	gliogenesis	5	133	0.032	0.147
all 275	GO:0030659	cytoplasmic vesicle membrane	5	133	0.032	0.147
all 275	GO:0012506	vesicle membrane	5	135	0.033	0.148
all 275	GO:0004252	serine-type endopeptidase activity	4	92	0.033	0.148
all 275	GO:0002684	positive regulation of immune system process	4	92	0.033	0.148
all 275	GO:0012501	programmed cell death	7	237	0.038	0.162
all 275	GO:0008219	cell death	7	237	0.038	0.162
all 275	GO:0006355	regulation of DNA-templated transcription	17	806	0.039	0.162
all 275	GO:0071363	cellular response to growth factor stimulus	5	141	0.039	0.162
all 275	GO:0007422	peripheral nervous system development	3	57	0.039	0.162
all 275	GO:0070848	response to growth factor	5	142	0.040	0.162
all 275	GO:0006814	sodium ion transport	4	98	0.041	0.162
all 275	GO:0007623	circadian rhythm	3	58	0.041	0.162
all 275	GO:0030198	extracellular matrix organization	5	144	0.042	0.162
all 275	GO:0043062	extracellular structure organization	5	144	0.042	0.162
all 275	GO:0042490	mechanoreceptor differentiation	3	59	0.043	0.163
all 275	GO:0002253	activation of immune response	3	60	0.045	0.167
all 275	GO:0035725	sodium ion transmembrane transport	3	61	0.047	0.170
all 275	GO:0016323	basolateral plasma membrane	3	61	0.047	0.170
all 275	GO:0008236	serine-type peptidase activity	4	104	0.049	0.176
all 275	GO:0017171	serine hydrolase activity	4	106	0.052	0.184
all 275	GO:0006865	amino acid transport	3	64	0.052	0.184
all 275	GO:0007167	enzyme-linked receptor protein signaling pathway	8	309	0.053	0.184
all 275	GO:0048882	lateral line development	4	108	0.055	0.188
all 275	GO:0001666	response to hypoxia	3	67	0.059	0.199
all 275	GO:0036293	response to decreased oxygen levels	3	68	0.061	0.204
all 275	GO:0004497	monooxygenase activity	4	113	0.063	0.207
all 275	GO:0051239	regulation of multicellular organismal process	9	377	0.063	0.207
all 275	GO:0009925	basal plasma membrane	3	70	0.065	0.209
all 275	GO:0045178	basal part of cell	3	70	0.065	0.209
all 275	GO:0070482	response to oxygen levels	3	71	0.067	0.214
all 275	GO:0048518	positive regulation of biological process	8	327	0.069	0.216
all 275	GO:0009897	external side of plasma membrane	4	118	0.071	0.220
all 275	GO:0048884	neuromast development	3	73	0.072	0.221
all 275	GO:0020037	heme binding	4	121	0.076	0.232
all 275	GO:0045597	positive regulation of cell differentiation	3	76	0.079	0.238
all 275	GO:0005506	iron ion binding	4	127	0.087	0.261
all 275	GO:0009953	dorsal/ventral pattern formation	4	130	0.093	0.275
all 275	GO:0002376	immune system process	9	413	0.097	0.284
all 275	GO:0030133	transport vesicle	5	186	0.100	0.290
all 275	GO:0005509	calcium ion binding	11	542	0.105	0.300
all 275	GO:0098793	presynapse	5	193	0.113	0.319
all 275	GO:0046983	protein dimerization activity	7	308	0.114	0.320
all 275	GO:0009719	response to endogenous stimulus	7	309	0.115	0.321
all 275	GO:0002682	regulation of immune system process	4	141	0.116	0.321
all 275	GO:0098609	cell-cell adhesion	6	253	0.119	0.324
all 275	GO:0009628	response to abiotic stimulus	7	313	0.121	0.328
all 275	GO:0048511	rhythmic process	3	93	0.125	0.331
all 275	GO:0000978	RNA polymerase II cis-regulatory region sequence-specific DNA binding	14	753	0.125	0.331
all 275	GO:0048583	regulation of response to stimulus	6	261	0.132	0.347
all 275	GO:0005912	adherens junction	3	97	0.136	0.356
all 275	GO:0098552	side of membrane	4	152	0.142	0.364
all 275	GO:0000987	cis-regulatory region sequence-specific DNA binding	14	771	0.142	0.364
all 275	GO:0061564	axon development	8	389	0.144	0.365
all 275	GO:0034220	monoatomic ion transmembrane transport	5	210	0.145	0.365
all 275	GO:0140097	catalytic activity, acting on DNA	4	154	0.146	0.366
all 275	GO:0008021	synaptic vesicle	3	101	0.149	0.368
all 275	GO:0000981	DNA-binding transcription factor activity, RNA polymerase II-specific	15	859	0.165	0.404
all 275	GO:0005911	cell-cell junction	5	223	0.173	0.416
all 275	GO:0009888	tissue development	16	934	0.173	0.416
all 275	GO:0004175	endopeptidase activity	6	284	0.174	0.416
all 275	GO:0005929	cilium	6	286	0.178	0.420
all 275	GO:0022008	neurogenesis	16	941	0.180	0.420
all 275	GO:0030855	epithelial cell differentiation	3	111	0.181	0.420
all 275	GO:0003729	mRNA binding	5	227	0.181	0.420
all 275	GO:0048522	positive regulation of cellular process	5	228	0.184	0.420
all 275	GO:0006302	double-strand break repair	3	112	0.184	0.420

all 275	GO:0016491	oxidoreductase activity	12	679	0.188	0.422
all 275	GO:0005667	transcription regulator complex	5	230	0.188	0.422
all 275	GO:0009986	cell surface	5	232	0.193	0.429
all 275	GO:0071495	cellular response to endogenous stimulus	5	233	0.195	0.430
all 275	GO:0030054	cell junction	14	820	0.196	0.430
all 275	GO:0009952	anterior/posterior pattern specification	4	178	0.209	0.453
all 275	GO:0006310	DNA recombination	3	120	0.211	0.453
all 275	GO:0019953	sexual reproduction	4	179	0.211	0.453
all 275	GO:0042592	homeostatic process	3	121	0.214	0.453
all 275	GO:0048871	multicellular organismal-level homeostasis	3	121	0.214	0.453
all 275	GO:0070382	exocytic vesicle	3	123	0.221	0.464
all 275	GO:0003002	regionalization	9	504	0.223	0.464
all 275	GO:0007276	gamete generation	3	124	0.225	0.465
all 275	GO:0016874	ligase activity	4	186	0.231	0.473
all 275	GO:0048609	multicellular organismal reproductive process	3	126	0.232	0.473
all 275	GO:0051094	positive regulation of developmental process	3	128	0.239	0.484
all 275	GO:0007389	pattern specification process	9	519	0.248	0.495
all 275	GO:0031410	cytoplasmic vesicle	13	792	0.248	0.495
all 275	GO:0097708	intracellular vesicle	13	795	0.252	0.495
all 275	GO:0048666	neuron development	11	658	0.253	0.495
all 275	GO:0051240	positive regulation of multicellular organismal process	3	132	0.253	0.495
all 275	GO:0006955	immune response	5	261	0.262	0.510
all 275	GO:0004518	nuclease activity	3	136	0.267	0.513
all 275	GO:2000145	regulation of cell motility	3	136	0.267	0.513
all 275	GO:0005764	lysosome	4	200	0.271	0.513
all 275	GO:0048699	generation of neurons	13	810	0.273	0.513
all 275	GO:0000323	lytic vacuole	4	201	0.274	0.513
all 275	GO:0000977	RNA polymerase II transcription regulatory region sequence-specific DNA binding	15	951	0.274	0.513
all 275	GO:0022414	reproductive process	4	203	0.280	0.520
all 275	GO:0040012	regulation of locomotion	3	142	0.288	0.533
all 275	GO:0003723	RNA binding	13	825	0.294	0.540
all 275	GO:0031982	vesicle	13	826	0.296	0.540
all 275	GO:0006325	chromatin organization	6	343	0.299	0.543
all 275	GO:0009314	response to radiation	3	146	0.303	0.546
all 275	GO:0048880	sensory system development	9	552	0.306	0.547
all 275	GO:0031175	neuron projection development	8	487	0.314	0.558
all 275	GO:0007169	cell surface receptor protein tyrosine kinase signaling pathway	4	215	0.315	0.558
all 275	GO:0045893	positive regulation of DNA-templated transcription	4	218	0.324	0.567
all 275	GO:0051726	regulation of cell cycle	3	152	0.324	0.567
all 275	GO:0006508	proteolysis	7	427	0.333	0.579
all 275	GO:0048839	inner ear development	3	159	0.349	0.604
all 275	GO:0043583	ear development	3	161	0.357	0.613
all 275	GO:0045202	synapse	8	514	0.367	0.619
all 275	GO:0007166	cell surface receptor signaling pathway	14	947	0.369	0.619
all 275	GO:0048667	cell morphogenesis involved in neuron differentiation	6	374	0.371	0.619
all 275	GO:0000785	chromatin	4	234	0.372	0.619
all 275	GO:0120543	macromolecular conformation isomerase activity	4	235	0.375	0.619
all 275	GO:0060429	epithelium development	8	518	0.375	0.619
all 275	GO:0070161	anchoring junction	5	305	0.375	0.619
all 275	GO:0055085	transmembrane transport	10	663	0.377	0.619
all 275	GO:0006811	monoatomic ion transport	8	522	0.383	0.621
all 275	GO:0010608	post-transcriptional regulation of gene expression	3	169	0.385	0.621
all 275	GO:0048523	negative regulation of cellular process	3	169	0.385	0.621
all 275	GO:0120025	plasma membrane bounded cell projection	7	452	0.387	0.621
all 275	GO:0031099	regeneration	3	170	0.389	0.621
all 275	GO:0035556	intracellular signal transduction	13	901	0.409	0.645
all 275	GO:0045892	negative regulation of DNA-templated transcription	3	176	0.410	0.645
all 275	GO:0005773	vacuole	4	247	0.411	0.645
all 275	GO:0022857	transmembrane transporter activity	8	540	0.419	0.655
all 275	GO:0016310	phosphorylation	12	836	0.423	0.657
all 275	GO:0001525	angiogenesis	4	252	0.425	0.657
all 275	GO:0006351	DNA-templated transcription	4	254	0.431	0.663
all 275	GO:0007423	sensory organ development	9	622	0.436	0.666

all 275	GO:0048519	negative regulation of biological process	4	260	0.449	0.682
all 275	GO:0010008	endosome membrane	3	189	0.455	0.689
all 275	GO:0030097	hemopoiesis	6	412	0.460	0.693
all 275	GO:0030182	neuron differentiation	11	788	0.466	0.698
all 275	GO:0009605	response to external stimulus	6	421	0.481	0.717
all 275	GO:0007417	central nervous system development	9	649	0.486	0.720
all 275	GO:0007409	axonogenesis	5	350	0.491	0.724
all 275	GO:0005085	guanyl-nucleotide exchange factor activity	3	204	0.506	0.735
all 275	GO:0003700	DNA-binding transcription factor activity	6	432	0.506	0.735
all 275	GO:0048598	embryonic morphogenesis	8	584	0.506	0.735
all 275	GO:0005694	chromosome	6	435	0.513	0.738
all 275	GO:0043228	membraneless organelle	6	435	0.513	0.738
all 275	GO:0016301	kinase activity	12	902	0.529	0.757
all 275	GO:0001944	vasculature development	6	444	0.533	0.759
all 275	GO:0140640	catalytic activity, acting on a nucleic acid	5	369	0.538	0.762
all 275	GO:0050877	nervous system process	4	296	0.550	0.775
all 275	GO:0005874	microtubule	3	223	0.566	0.794
all 275	GO:0006259	DNA metabolic process	5	384	0.573	0.801
all 275	GO:0008270	zinc ion binding	8	621	0.576	0.801
all 275	GO:0035239	tube morphogenesis	6	468	0.584	0.809
all 275	GO:0048812	neuron projection morphogenesis	5	394	0.596	0.815
all 275	GO:0008233	peptidase activity	6	476	0.601	0.815
all 275	GO:0006629	lipid metabolic process	5	397	0.603	0.815
all 275	GO:0060322	head development	6	477	0.603	0.815
all 275	GO:0006468	protein phosphorylation	4	317	0.604	0.815
all 275	GO:0016462	pyrophosphatase activity	8	639	0.608	0.815
all 275	GO:0048514	blood vessel morphogenesis	4	319	0.609	0.815
all 275	GO:0140657	ATP-dependent activity	4	323	0.618	0.818
all 275	GO:0016818	hydrolase activity, acting on acid anhydrides, in phosphorus-containing anhydrides	8	645	0.619	0.818
all 275	GO:0016817	hydrolase activity, acting on acid anhydrides	8	645	0.619	0.818
all 275	GO:0006950	response to stress	12	964	0.622	0.818
all 275	GO:0061024	membrane organization	3	246	0.632	0.821
all 275	GO:0048870	cell motility	6	492	0.633	0.821
all 275	GO:0003924	GTPase activity	4	329	0.633	0.821
all 275	GO:0005768	endosome	5	412	0.635	0.821
all 275	GO:0005730	nucleolus	3	249	0.640	0.824
all 275	GO:0060041	retina development in camera-type eye	3	251	0.646	0.827
all 275	GO:0005525	GTP binding	5	425	0.662	0.844
all 275	GO:0048018	receptor ligand activity	3	266	0.684	0.865
all 275	GO:0030546	signaling receptor activator activity	3	268	0.689	0.865
all 275	GO:0140110	transcription regulator activity	3	270	0.694	0.865
all 275	GO:0048646	anatomical structure formation involved in morphogenesis	7	609	0.695	0.865
all 275	GO:0006338	chromatin remodeling	3	271	0.696	0.865
all 275	GO:0016853	isomerase activity	4	360	0.702	0.865
all 275	GO:0141124	intracellular signaling cassette	4	362	0.706	0.865
all 275	GO:0016477	cell migration	5	448	0.707	0.865
all 275	GO:0001568	blood vessel development	4	363	0.708	0.865
all 275	GO:0072359	circulatory system development	10	865	0.708	0.865
all 275	GO:0006281	DNA repair	3	281	0.719	0.874
all 275	GO:0006974	DNA damage response	4	370	0.722	0.874
all 275	GO:0007420	brain development	5	458	0.725	0.874
all 275	GO:0015629	actin cytoskeleton	3	286	0.730	0.877
all 275	GO:0065003	protein-containing complex assembly	4	382	0.744	0.891
all 275	GO:0016192	vesicle-mediated transport	5	476	0.755	0.895
all 275	GO:0043010	camera-type eye development	4	388	0.755	0.895
all 275	GO:0005783	endoplasmic reticulum	8	735	0.757	0.895
all 275	GO:0035295	tube development	6	568	0.763	0.898
all 275	GO:0005654	nucleoplasm	5	492	0.780	0.915
all 275	GO:0005789	endoplasmic reticulum membrane	5	496	0.786	0.915
all 275	GO:0017111	ribonucleoside triphosphate phosphatase activity	6	585	0.787	0.915
all 275	GO:0031981	nuclear lumen	8	767	0.796	0.923
all 275	GO:0042175	nuclear outer membrane-endoplasmic reticulum membrane network	5	506	0.800	0.923
all 275	GO:0043933	protein-containing complex organization	4	435	0.828	0.948
all 275	GO:0015630	microtubule cytoskeleton	5	530	0.831	0.948
all 275	GO:0005739	mitochondrion	10	974	0.831	0.948
all 275	GO:0042995	cell projection	4	443	0.839	0.950
all 275	GO:0048568	embryonic organ development	5	544	0.847	0.950
all 275	GO:0004930	G protein-coupled receptor activity	3	354	0.848	0.950

all 275	GO:0016755	aminoacyltransferase activity	3	357	0.852	0.950
all 275	GO:0016773	phosphotransferase activity, alcohol group as acceptor	7	732	0.853	0.950
all 275	GO:0007017	microtubule-based process	3	358	0.854	0.950
all 275	GO:0150063	visual system development	4	460	0.859	0.950
all 275	GO:0001654	eye development	4	460	0.859	0.950
all 275	GO:0006810	transport	3	369	0.867	0.956
all 275	GO:0048562	embryonic organ morphogenesis	3	377	0.877	0.962
all 275	GO:0070013	intracellular organelle lumen	8	863	0.886	0.968
all 275	GO:0038023	signaling receptor activity	7	784	0.896	0.968
all 275	GO:0060089	molecular transducer activity	7	784	0.896	0.968
all 275	GO:0009887	animal organ morphogenesis	6	692	0.897	0.968
all 275	GO:0140513	nuclear protein-containing complex	6	694	0.898	0.968
all 275	GO:0005829	cytosol	8	898	0.909	0.976
all 275	GO:0045184	establishment of protein localization	3	423	0.920	0.984
all 275	GO:0004888	transmembrane signaling receptor activity	5	657	0.937	0.991
all 275	GO:0051234	establishment of localization	6	757	0.937	0.991
all 275	GO:0004674	protein serine/threonine kinase activity	3	448	0.937	0.991
all 275	GO:0033554	cellular response to stress	4	560	0.940	0.991
all 275	GO:0007186	G protein-coupled receptor signaling pathway	3	490	0.958	0.998
all 275	GO:0016788	hydrolase activity, acting on ester bonds	4	609	0.961	0.998
all 275	GO:0007507	heart development	3	499	0.962	0.998
all 275	GO:0004672	protein kinase activity	4	614	0.963	0.998
all 275	GO:0005102	signaling receptor binding	3	520	0.969	0.998
all 275	GO:1990904	ribonucleoprotein complex	3	520	0.969	0.998
all 275	GO:0043005	neuron projection	3	524	0.970	0.998
all 275	GO:0031967	organelle envelope	3	541	0.975	0.999
all 275	GO:0016746	acyltransferase activity	3	565	0.981	0.999
all 275	GO:0016043	cellular component organization	4	706	0.985	0.999
all 275	GO:0008104	intracellular protein localization	3	619	0.989	0.999
all 275	GO:0008092	cytoskeletal protein binding	3	644	0.992	0.999
all 275	GO:0033036	macromolecule localization	3	717	0.996	0.999
all 275	GO:0031090	organelle membrane	4	914	0.998	0.999
all 275	GO:0071840	cellular component organization or biogenesis	4	963	0.999	0.999
after exclusion	GO:0005581	collagen trimer	6	56	5.13×10^{-5}	0.013
after exclusion	GO:0045595	regulation of cell differentiation	9	163	0.00015	0.019
after exclusion	GO:0050767	regulation of neurogenesis	7	109	0.00032	0.027
after exclusion	GO:0060284	regulation of cell development	7	114	0.00042	0.027
after exclusion	GO:0051960	regulation of nervous system development	7	121	0.0006	0.031
after exclusion	GO:0048935	peripheral nervous system neuron development	3	20	0.00161	0.054
after exclusion	GO:0048934	peripheral nervous system neuron differentiation	3	20	0.00161	0.054
after exclusion	GO:0005604	basement membrane	4	43	0.00165	0.054
after exclusion	GO:0008395	steroid hydroxylase activity	3	23	0.00244	0.058
after exclusion	GO:0042552	myelination	4	48	0.00248	0.058
after exclusion	GO:0008366	axon ensheathment	4	49	0.00268	0.058
after exclusion	GO:0007272	ensheathment of neurons	4	49	0.00268	0.058
after exclusion	GO:0014003	oligodendrocyte development	3	26	0.00349	0.070
after exclusion	GO:0015291	secondary active transmembrane transporter activity	6	125	0.00377	0.070
after exclusion	GO:0022804	active transmembrane transporter activity	6	127	0.00407	0.071
after exclusion	GO:0050793	regulation of developmental process	10	330	0.00617	0.100
after exclusion	GO:0005507	copper ion binding	3	33	0.00688	0.105
after exclusion	GO:2000026	regulation of multicellular organismal development	9	288	0.00758	0.109
after exclusion	GO:0015293	symporter activity	4	68	0.00864	0.114
after exclusion	GO:0032922	circadian regulation of gene expression	3	36	0.00878	0.114
after exclusion	GO:0007179	transforming growth factor beta receptor signaling pathway	3	39	0.011	0.124
after exclusion	GO:0071560	cellular response to transforming growth factor beta stimulus	3	39	0.011	0.124
after exclusion	GO:0071559	response to transforming growth factor beta	3	39	0.011	0.124
after exclusion	GO:0030020	extracellular matrix structural constituent conferring tensile strength	3	41	0.013	0.129
after exclusion	GO:0000381	regulation of alternative mRNA splicing, via spliceosome	3	41	0.013	0.129
after exclusion	GO:0015804	neutral amino acid transport	3	42	0.013	0.129
after exclusion	GO:0002252	immune effector process	3	42	0.013	0.129
after exclusion	GO:0048709	oligodendrocyte differentiation	3	43	0.014	0.133

after exclusion	GO:0141091	transforming growth factor beta receptor superfamily signaling pathway	4	80	0.015	0.135
after exclusion	GO:0006355	regulation of DNA-templated transcription	17	806	0.016	0.137
after exclusion	GO:0005576	extracellular region	16	747	0.017	0.137
after exclusion	GO:0006915	apoptotic process	7	222	0.017	0.137
after exclusion	GO:0016705	oxidoreductase activity, acting on paired donors, with incorporation or reduction of molecular oxygen	6	174	0.018	0.139
after exclusion	GO:0007178	cell surface receptor protein serine/threonine kinase signaling pathway	4	85	0.018	0.141
after exclusion	GO:0030659	cytoplasmic vesicle membrane	5	133	0.021	0.154
after exclusion	GO:0005201	extracellular matrix structural constituent	3	50	0.021	0.154
after exclusion	GO:0012506	vesicle membrane	5	135	0.022	0.154
after exclusion	GO:0008219	cell death	7	237	0.023	0.154
after exclusion	GO:0012501	programmed cell death	7	237	0.023	0.154
after exclusion	GO:0071363	cellular response to growth factor stimulus	5	141	0.026	0.172
after exclusion	GO:0070848	response to growth factor	5	142	0.027	0.172
after exclusion	GO:0006814	sodium ion transport	4	98	0.029	0.177
after exclusion	GO:0005882	intermediate filament	3	57	0.030	0.177
after exclusion	GO:0007422	peripheral nervous system development	3	57	0.030	0.177
after exclusion	GO:0007167	enzyme-linked receptor protein signaling pathway	8	309	0.031	0.177
after exclusion	GO:0007623	circadian rhythm	3	58	0.031	0.177
after exclusion	GO:0045111	intermediate filament cytoskeleton	3	59	0.033	0.178
after exclusion	GO:0042490	mechanoreceptor differentiation	3	59	0.033	0.178
after exclusion	GO:0016323	basolateral plasma membrane	3	61	0.036	0.185
after exclusion	GO:0035725	sodium ion transmembrane transport	3	61	0.036	0.185
after exclusion	GO:0051239	regulation of multicellular organismal process	9	377	0.036	0.185
after exclusion	GO:0006865	amino acid transport	3	64	0.040	0.198
after exclusion	GO:0031012	extracellular matrix	5	159	0.041	0.198
after exclusion	GO:0030312	external encapsulating structure	5	159	0.041	0.198
after exclusion	GO:0001666	response to hypoxia	3	67	0.045	0.211
after exclusion	GO:0004497	monooxygenase activity	4	113	0.045	0.211
after exclusion	GO:0036293	response to decreased oxygen levels	3	68	0.047	0.212
after exclusion	GO:0048925	lateral line system development	4	116	0.049	0.212
after exclusion	GO:0009925	basal plasma membrane	3	70	0.050	0.212
after exclusion	GO:0045178	basal part of cell	3	70	0.050	0.212
after exclusion	GO:0007156	homophilic cell-cell adhesion	4	117	0.050	0.212
after exclusion	GO:0009897	external side of plasma membrane	4	118	0.052	0.212
after exclusion	GO:0050778	positive regulation of immune response	3	71	0.052	0.212
after exclusion	GO:0070482	response to oxygen levels	3	71	0.052	0.212
after exclusion	GO:0020037	heme binding	4	121	0.056	0.223
after exclusion	GO:0010001	glial cell differentiation	4	123	0.059	0.231
after exclusion	GO:0045597	positive regulation of cell differentiation	3	76	0.061	0.238
after exclusion	GO:0005506	iron ion binding	4	127	0.064	0.244
after exclusion	GO:0000978	RNA polymerase II cis-regulatory region sequence-specific DNA binding	14	753	0.065	0.244
after exclusion	GO:0009953	dorsal/ventral pattern formation	4	130	0.069	0.256
after exclusion	GO:0030133	transport vesicle	5	186	0.071	0.259
after exclusion	GO:0042063	gliogenesis	4	133	0.074	0.262
after exclusion	GO:0046983	protein dimerization activity	7	308	0.074	0.262
after exclusion	GO:0000987	cis-regulatory region sequence-specific DNA binding	14	771	0.075	0.262
after exclusion	GO:0009719	response to endogenous stimulus	7	309	0.075	0.262
after exclusion	GO:0099080	supramolecular complex	10	505	0.079	0.269
after exclusion	GO:0098793	presynapse	5	193	0.080	0.270
after exclusion	GO:0050776	regulation of immune response	3	86	0.082	0.274
after exclusion	GO:0048584	positive regulation of response to stimulus	3	88	0.087	0.279
after exclusion	GO:0021782	glial cell development	3	88	0.087	0.279
after exclusion	GO:0000981	DNA-binding transcription factor activity, RNA polymerase II-specific	15	859	0.087	0.279
after exclusion	GO:0043062	extracellular structure organization	4	144	0.092	0.286
after exclusion	GO:0030198	extracellular matrix organization	4	144	0.092	0.286
after exclusion	GO:0061564	axon development	8	389	0.092	0.286
after exclusion	GO:0007155	cell adhesion	9	456	0.094	0.286
after exclusion	GO:0048518	positive regulation of biological process	7	327	0.095	0.287
after exclusion	GO:0002684	positive regulation of immune system process	3	92	0.096	0.287

after exclusion	GO:0048511	rhythmic process	3	93	0.098	0.290
after exclusion	GO:0034220	monoatomic ion transmembrane transport	5	210	0.105	0.306
after exclusion	GO:0098552	side of membrane	4	152	0.107	0.308
after exclusion	GO:0016491	oxidoreductase activity	12	679	0.110	0.313
after exclusion	GO:0140097	catalytic activity, acting on DNA	4	154	0.111	0.313
after exclusion	GO:0008021	synaptic vesicle	3	101	0.118	0.328
after exclusion	GO:0002376	immune system process	8	413	0.119	0.328
after exclusion	GO:0003729	mRNA binding	5	227	0.133	0.364
after exclusion	GO:0048522	positive regulation of cellular process	5	228	0.135	0.365
after exclusion	GO:0048882	lateral line development	3	108	0.137	0.366
after exclusion	GO:0005667	transcription regulator complex	5	230	0.138	0.367
after exclusion	GO:0009986	cell surface	5	232	0.142	0.373
after exclusion	GO:0071495	cellular response to endogenous stimulus	5	233	0.144	0.373
after exclusion	GO:0003002	regionalization	9	504	0.146	0.373
after exclusion	GO:0006302	double-strand break repair	3	112	0.148	0.373
after exclusion	GO:0031410	cytoplasmic vesicle	13	792	0.148	0.373
after exclusion	GO:0097708	intracellular vesicle	13	795	0.151	0.377
after exclusion	GO:0000977	RNA polymerase II transcription regulatory region sequence-specific DNA binding	15	951	0.159	0.391
after exclusion	GO:0048666	neuron development	11	658	0.159	0.391
after exclusion	GO:0009952	anterior/posterior pattern specification	4	178	0.161	0.391
after exclusion	GO:0007389	pattern specification process	9	519	0.164	0.395
after exclusion	GO:0005198	structural molecule activity	4	180	0.166	0.395
after exclusion	GO:0009628	response to abiotic stimulus	6	313	0.168	0.398
after exclusion	GO:0006310	DNA recombination	3	120	0.170	0.399
after exclusion	GO:0070382	exocytic vesicle	3	123	0.179	0.413
after exclusion	GO:0016874	ligase activity	4	186	0.179	0.413
after exclusion	GO:0003723	RNA binding	13	825	0.182	0.413
after exclusion	GO:0031982	vesicle	13	826	0.183	0.413
after exclusion	GO:0051094	positive regulation of developmental process	3	128	0.194	0.435
after exclusion	GO:0048583	regulation of response to stimulus	5	261	0.198	0.437
after exclusion	GO:0099513	polymeric cytoskeletal fiber	6	330	0.198	0.437
after exclusion	GO:0051240	positive regulation of multicellular organismal process	3	132	0.206	0.450
after exclusion	GO:0005764	lysosome	4	200	0.213	0.461
after exclusion	GO:0000323	lytic vacuole	4	201	0.216	0.461
after exclusion	GO:2000145	regulation of cell motility	3	136	0.218	0.461
after exclusion	GO:0004518	nuclease activity	3	136	0.218	0.461
after exclusion	GO:0031175	neuron projection development	8	487	0.222	0.462
after exclusion	GO:0006325	chromatin organization	6	343	0.222	0.462
after exclusion	GO:0022008	neurogenesis	14	941	0.227	0.468
after exclusion	GO:0007166	cell surface receptor signaling pathway	14	947	0.234	0.475
after exclusion	GO:0002682	regulation of immune system process	3	141	0.234	0.475
after exclusion	GO:0040012	regulation of locomotion	3	142	0.237	0.478
after exclusion	GO:0099081	supramolecular polymer	7	431	0.251	0.492
after exclusion	GO:0099512	supramolecular fiber	7	431	0.251	0.492
after exclusion	GO:0007169	cell surface receptor protein tyrosine kinase signaling pathway	4	215	0.251	0.492
after exclusion	GO:0005929	cilium	5	286	0.252	0.492
after exclusion	GO:0048699	generation of neurons	12	810	0.254	0.492
after exclusion	GO:0045893	positive regulation of DNA-templated transcription	4	218	0.259	0.498
after exclusion	GO:0055085	transmembrane transport	10	663	0.262	0.500
after exclusion	GO:0045202	synapse	8	514	0.265	0.504
after exclusion	GO:0051726	regulation of cell cycle	3	152	0.269	0.506
after exclusion	GO:0035556	intracellular signal transduction	13	901	0.271	0.508
after exclusion	GO:0006811	monoatomic ion transport	8	522	0.279	0.518
after exclusion	GO:0048667	cell morphogenesis involved in neuron differentiation	6	374	0.283	0.522
after exclusion	GO:0016310	phosphorylation	12	836	0.288	0.528
after exclusion	GO:0048839	inner ear development	3	159	0.291	0.529
after exclusion	GO:0043583	ear development	3	161	0.298	0.537
after exclusion	GO:0000785	chromatin	4	234	0.301	0.539
after exclusion	GO:0120543	macromolecular conformation isomerase activity	4	235	0.304	0.541
after exclusion	GO:0022857	transmembrane transporter activity	8	540	0.310	0.548
after exclusion	GO:0005509	calcium ion binding	8	542	0.313	0.551
after exclusion	GO:0048523	negative regulation of cellular process	3	169	0.324	0.565
after exclusion	GO:0030182	neuron differentiation	11	788	0.332	0.575
after exclusion	GO:0005773	vacuole	4	247	0.336	0.578
after exclusion	GO:0045892	negative regulation of DNA-templated transcription	3	176	0.346	0.592
after exclusion	GO:0001525	angiogenesis	4	252	0.349	0.593

after exclusion	GO:0098609	cell-cell adhesion	4	253	0.352	0.593
after exclusion	GO:0006351	DNA-templated transcription	4	254	0.354	0.593
after exclusion	GO:0019953	sexual reproduction	3	179	0.356	0.593
after exclusion	GO:0030097	hemopoiesis	6	412	0.362	0.596
after exclusion	GO:0007417	central nervous system development	9	649	0.362	0.596
after exclusion	GO:0048519	negative regulation of biological process	4	260	0.370	0.606
after exclusion	GO:0006955	immune response	4	261	0.373	0.606
after exclusion	GO:0016301	kinase activity	12	902	0.381	0.616
after exclusion	GO:0010008	endosome membrane	3	189	0.388	0.623
after exclusion	GO:0007409	axonogenesis	5	350	0.400	0.637
after exclusion	GO:0003700	DNA-binding transcription factor activity	6	432	0.404	0.641
after exclusion	GO:0043228	membraneless organelle	6	435	0.411	0.643
after exclusion	GO:0005694	chromosome	6	435	0.411	0.643
after exclusion	GO:0009888	tissue development	12	934	0.428	0.666
after exclusion	GO:0022414	reproductive process	3	203	0.432	0.669
after exclusion	GO:0005085	guanyl-nucleotide exchange factor activity	3	204	0.435	0.670
after exclusion	GO:0140640	catalytic activity, acting on a nucleic acid	5	369	0.444	0.679
after exclusion	GO:0008270	zinc ion binding	8	621	0.455	0.691
after exclusion	GO:0006950	response to stress	12	964	0.471	0.712
after exclusion	GO:0006259	DNA metabolic process	5	384	0.478	0.718
after exclusion	GO:0048880	sensory system development	7	552	0.481	0.718
after exclusion	GO:0016462	pyrophosphatase activity	8	639	0.487	0.723
after exclusion	GO:0005874	microtubule	3	223	0.493	0.723
after exclusion	GO:0016818	hydrolase activity, acting on acid anhydrides, in phosphorus-containing anhydrides	8	645	0.497	0.723
after exclusion	GO:0016817	hydrolase activity, acting on acid anhydrides	8	645	0.497	0.723
after exclusion	GO:0060322	head development	6	477	0.498	0.723
after exclusion	GO:0048812	neuron projection morphogenesis	5	394	0.501	0.723
after exclusion	GO:0030054	cell junction	10	820	0.507	0.725
after exclusion	GO:0006629	lipid metabolic process	5	397	0.507	0.725
after exclusion	GO:0006468	protein phosphorylation	4	317	0.519	0.737
after exclusion	GO:0048514	blood vessel morphogenesis	4	319	0.524	0.740
after exclusion	GO:0140657	ATP-dependent activity	4	323	0.534	0.750
after exclusion	GO:0048598	embryonic morphogenesis	7	584	0.540	0.751
after exclusion	GO:0005768	endosome	5	412	0.540	0.751
after exclusion	GO:0003924	GTPase activity	4	329	0.548	0.758
after exclusion	GO:0061024	membrane organization	3	246	0.559	0.766
after exclusion	GO:0009605	response to external stimulus	5	421	0.560	0.766
after exclusion	GO:0005730	nucleolus	3	249	0.568	0.769
after exclusion	GO:0005525	GTP binding	5	425	0.568	0.769
after exclusion	GO:0048646	anatomical structure formation involved in morphogenesis	7	609	0.585	0.788
after exclusion	GO:0001944	vasculature development	5	444	0.607	0.802
after exclusion	GO:0007423	sensory organ development	7	622	0.607	0.802
after exclusion	GO:0048018	receptor ligand activity	3	266	0.612	0.802
after exclusion	GO:0030546	signaling receptor activator activity	3	268	0.617	0.802
after exclusion	GO:0016853	isomerase activity	4	360	0.619	0.802
after exclusion	GO:0140110	transcription regulator activity	3	270	0.622	0.802
after exclusion	GO:0120025	plasma membrane bounded cell projection	5	452	0.623	0.802
after exclusion	GO:0141124	intracellular signaling cassette	4	362	0.624	0.802
after exclusion	GO:0006338	chromatin remodeling	3	271	0.625	0.802
after exclusion	GO:0001568	blood vessel development	4	363	0.626	0.802
after exclusion	GO:0007420	brain development	5	458	0.634	0.808
after exclusion	GO:0006974	DNA damage response	4	370	0.641	0.813
after exclusion	GO:0005783	endoplasmic reticulum	8	735	0.645	0.814
after exclusion	GO:0006281	DNA repair	3	281	0.649	0.816
after exclusion	GO:0035239	tube morphogenesis	5	468	0.653	0.816
after exclusion	GO:0004175	endopeptidase activity	3	284	0.656	0.817
after exclusion	GO:0065003	protein-containing complex assembly	4	382	0.665	0.824
after exclusion	GO:0031981	nuclear lumen	8	767	0.691	0.845
after exclusion	GO:0017111	ribonucleoside triphosphate phosphatase activity	6	585	0.695	0.845
after exclusion	GO:0005654	nucleoplasm	5	492	0.695	0.845
after exclusion	GO:0048870	cell motility	5	492	0.695	0.845
after exclusion	GO:0005789	endoplasmic reticulum membrane	5	496	0.702	0.849
after exclusion	GO:0042175	nuclear outer membrane-endoplasmic reticulum membrane network	5	506	0.719	0.865
after exclusion	GO:0005739	mitochondrion	10	974	0.722	0.865
after exclusion	GO:0060429	epithelium development	5	518	0.737	0.879
after exclusion	GO:0006508	proteolysis	4	427	0.747	0.887
after exclusion	GO:0015630	microtubule cytoskeleton	5	530	0.755	0.893
after exclusion	GO:0043933	protein-containing complex organization	4	435	0.760	0.894

after exclusion	GO:0016773	phosphotransferase activity, alcohol group as acceptor	7	732	0.768	0.899
after exclusion	GO:0042995	cell projection	4	443	0.772	0.899
after exclusion	GO:0048568	embryonic organ development	5	544	0.775	0.899
after exclusion	GO:0016477	cell migration	4	448	0.780	0.901
after exclusion	GO:0016755	aminoacyltransferase activity	3	357	0.797	0.913
after exclusion	GO:0007017	microtubule-based process	3	358	0.799	0.913
after exclusion	GO:0035295	tube development	5	568	0.806	0.913
after exclusion	GO:0070013	intracellular organelle lumen	8	863	0.806	0.913
after exclusion	GO:0072359	circulatory system development	8	865	0.808	0.913
after exclusion	GO:0006810	transport	3	369	0.815	0.917
after exclusion	GO:0016192	vesicle-mediated transport	4	476	0.818	0.917
after exclusion	GO:0048562	embryonic organ morphogenesis	3	377	0.826	0.922
after exclusion	GO:0009887	animal organ morphogenesis	6	692	0.832	0.923
after exclusion	GO:0140513	nuclear protein-containing complex	6	694	0.834	0.923
after exclusion	GO:0043010	camera-type eye development	3	388	0.841	0.926
after exclusion	GO:0045184	establishment of protein localization	3	423	0.880	0.965
after exclusion	GO:0051234	establishment of localization	6	757	0.888	0.968
after exclusion	GO:0033554	cellular response to stress	4	560	0.901	0.968
after exclusion	GO:0004674	protein serine/threonine kinase activity	3	448	0.903	0.968
after exclusion	GO:0038023	signaling receptor activity	6	784	0.906	0.968
after exclusion	GO:0060089	molecular transducer activity	6	784	0.906	0.968
after exclusion	GO:0005829	cytosol	7	898	0.911	0.968
after exclusion	GO:0001654	eye development	3	460	0.912	0.968
after exclusion	GO:0150063	visual system development	3	460	0.912	0.968
after exclusion	GO:0008233	peptidase activity	3	476	0.923	0.976
after exclusion	GO:0016788	hydrolase activity, acting on ester bonds	4	609	0.933	0.980
after exclusion	GO:0004672	protein kinase activity	4	614	0.935	0.980
after exclusion	GO:1990904	ribonucleoprotein complex	3	520	0.948	0.983
after exclusion	GO:0005102	signaling receptor binding	3	520	0.948	0.983
after exclusion	GO:0043005	neuron projection	3	524	0.950	0.983
after exclusion	GO:0004888	transmembrane signaling receptor activity	4	657	0.954	0.983
after exclusion	GO:0031967	organelle envelope	3	541	0.957	0.983
after exclusion	GO:0016746	acyltransferase activity	3	565	0.965	0.988
after exclusion	GO:0016043	cellular component organization	4	706	0.970	0.989
after exclusion	GO:0008104	intracellular protein localization	3	619	0.979	0.994
after exclusion	GO:0008092	cytoskeletal protein binding	3	644	0.983	0.995
after exclusion	GO:0033036	macromolecule localization	3	717	0.992	0.997
after exclusion	GO:0031090	organelle membrane	4	914	0.995	0.997
after exclusion	GO:0071840	cellular component organization or biogenesis	4	963	0.997	0.997

Table S20. g:Profiler output for the full list of 275 DEGs and after removal of the twenty-eight reassigned genes, sorted so that terms reaching FDR < 0.05 appear first within each analysis. Sixteen terms are significant on the full list and five after the reassignment.

Table S21. Cluster membership after the reassignment

Cluster	N before	N after	Removed	Remaining
MCL 1	9	9	—	nog2, irf3, cdk5, smad6a, tgfb1a, hey1, smad3a, jun, gpx1a
MCL 2	8	7	col17a1a	col8a2, col12a1a, col16a1, p3h1, bmp1b, col4a6, lamb2l
MCL 3	7	3	krt17, krt4, krt5, pkp1b	tfpt, hephl1a, zgc:136930
MCL 4	6	5	ela2l	her4.5, her8.2, ifi27.4, chrna7a, loxl2b
MCL 5	5	4	ampd1	mcm7, rrm2b, tk2, entpd8
MCL 6	5	4	upk1a	si:ch211-39f2.3, si:dkey-247k7.2, moxd1, si:dkey-183i3.5

Table S21. MCL clusters before and after removal of the reassigned genes.

Table S22. Composite scores for the twelve marker panels

Panel	Ctrl1	Ctrl2	Ctrl3	Str1	Str2	Str3
neuron	11.84	11.91	11.94	11.97	11.88	11.81
radial_glia	10.15	9.94	10.06	10.37	10.19	10.36
oligodendrocyte	8.30	8.31	8.55	8.42	8.17	7.81
microglia	6.57	6.64	6.65	6.63	6.70	6.99
epidermis	5.97	7.71	6.82	5.37	5.42	4.14
olfactory_epithelium	6.95	7.44	6.94	6.83	6.95	7.07
erythrocyte	9.59	9.95	10.29	9.69	9.57	9.08
endothelium	8.17	8.70	8.44	8.46	8.34	8.28
pineal	7.80	5.23	5.67	5.39	6.13	5.23
skeletal_muscle	4.22	4.55	4.08	4.38	4.03	4.00
cartilage_bone	4.93	5.47	5.58	4.67	6.51	4.71
lens_eye	7.67	2.82	3.12	1.11	7.78	2.45

Table S22. Composite scores per library, centred within panels, with the difference between groups and its nominal p-value. These are descriptive; the multiplicity correction is in Table S26.

Table S23. Positional gene pairs among the differentially expressed genes

Chr	GeneA id	GeneA	GeneB id	GeneB	Start distance bp	Body gap bp	L2fcA	L2fcB	Same dir
1	ENSDARG00000096599	si:ch211-160d14.9	ENSDARG00000100771	ENSDARG00000100771	6 330	3 551	-8.50	-5.98	yes
1	ENSDARG00000113858	ENSDARG00000113858	ENSDARG00000096599	si:ch211-160d14.9	8 033	1 164	-7.83	-8.50	yes
11	ENSDARG00000057093	kdm5ba	ENSDARG00000029510	timm17a	80 799	48 659	-0.410	-0.559	yes
11	ENSDARG00000070213	etnk2	ENSDARG00000061000	klhdc8a	184 410	152 623	0.740	0.677	yes
11	ENSDARG00000100115	dnah1	ENSDARG00000027065	slc38a3a	190 707	103 469	0.929	-0.730	no
13	ENSDARG00000017489	ifi27.1	ENSDARG00000078389	ifi27.4	17 380	14 835	2.11	1.46	yes
14	ENSDARG00000061909	si:ch73-233f7.1	ENSDARG00000104806	fbxw11a	166 623	148 645	0.443	-0.689	no
15	ENSDARG00000091847	LOC110437747	ENSDARG00000068621	nlrc11	18 146	4 839	6.73	8.65	yes
15	ENSDARG00000068840	ppt2a.1	ENSDARG00000091607	LOC101884284	64 641	59 716	5.49	2.14	yes
15	ENSDARG00000069888	cldna	ENSDARG00000041502	tgfb1a	120 940	118 998	-1.51	0.698	no
16	ENSDARG00000022430	ppp1r8b	ENSDARG00000089572	tfpt	18 133	73	0.794	0.932	yes
16	ENSDARG00000009978	icn	ENSDARG00000055514	icn2	55 862	54 499	-1.18	-3.78	yes
16	ENSDARG00000103199	si:dkey-247k7.2	ENSDARG00000009978	icn	92 127	85 094	-2.79	-1.18	yes
17	ENSDARG00000032426	coq8ab	ENSDARG00000075619	cenpo	60 669	40 254	1.92	-4.29	no
18	ENSDARG00000041691	bhlhe41	ENSDARG00000057698	ctsd	7 743	3 430	0.531	0.333	yes
18	ENSDARG00000069189	ENSDARG00000069189	ENSDARG00000041450	rab11a	10 789	7 461	3.08	0.382	yes
18	ENSDARG00000057698	ctsd	ENSDARG00000062821	slc6a15	18 179	5 693	0.333	0.558	yes
18	ENSDARG00000016038	hacd3	ENSDARG00000069189	ENSDARG00000069189	138 907	125 701	0.577	3.08	yes
18	ENSDARG00000093381	tgm2l	ENSDARG00000016038	hacd3	178 538	131 775	-1.43	0.577	no
2	ENSDARG00000097556	cyp8b1.2	ENSDARG00000097559	cyp8b1.3	15 479	13 513	-3.17	-7.04	yes
21	ENSDARG00000090202	rbm11	ENSDARG00000079393	tmprss15	15 950	2 635	-1.21	-1.44	yes
21	ENSDARG00000056248	si:dkey-183i3.5	ENSDARG00000044655	st14b	88 483	78 590	-2.60	-3.65	yes
21	ENSDARG00000056619	arhgap42a	ENSDARG00000044619	birc2	94 966	37 841	0.619	0.577	yes
21	ENSDARG00000057121	c7b	ENSDARG00000043608	eif4ebp1	111 642	97 856	-0.887	1.03	no
23	ENSDARG00000070770	her4.3	ENSDARG00000056729	her4.5	9 940	8 063	3.53	2.76	yes
23	ENSDARG00000071029	fbxo44.9	ENSDARG00000092077	ENSDARG00000092077	19 543	2 608	2.02	5.55	yes
25	ENSDARG00000029232	ENSDARG00000029232	ENSDARG00000076025	dgkzb	193 289	183 021	-0.901	0.365	no
3	ENSDARG00000097238	ba1l	ENSDARG00000089087	hbba1	4 988	4 173	-0.602	-0.506	yes
3	ENSDARG00000102593	si:ch211-214b16.2	ENSDARG00000074345	si:ch211-214b16.3	24 714	22 870	6.65	3.28	yes
3	ENSDARG00000075626	zgc:172090	ENSDARG00000087012	ENSDARG00000087012	30 501	26 930	5.23	5.69	yes
5	ENSDARG00000028542	si:ch211-114c12.2	ENSDARG00000057855	mrps31	9 538	2 877	-0.544	-0.684	yes
6	ENSDARG00000099307	si:dkey-10p5.10	ENSDARG00000098924	suz12b	11 073	7 986	-1.22	-0.379	yes
6	ENSDARG00000052705	pkp1b	ENSDARG00000052708	tnni1b	93 830	51 094	-2.69	1.23	no
6	ENSDARG00000034409	pik3r3b	ENSDARG00000074311	mast2	126 399	104 689	-1.00	-0.789	yes
6	ENSDARG00000005565	entpd8	ENSDARG00000044280	opn1mw2	147 563	125 897	-2.38	5.77	no
7	ENSDARG00000087196	fibinb	ENSDARG00000003495	madd	27 350	24 328	-4.72	-0.327	yes
7	ENSDARG00000053200	dis3l	ENSDARG00000087196	fibinb	48 732	33 677	-1.04	-4.72	yes
7	ENSDARG00000053209	smad6a	ENSDARG00000053200	dis3l	58 356	48 485	-1.66	-1.04	yes
7	ENSDARG00000036096	smad3a	ENSDARG00000053209	smad6a	80 302	33 700	-2.36	-1.66	yes
7	ENSDARG00000090615	cd59	ENSDARG00000017474	zgc:110699	116 081	113 136	-0.644	0.748	no
7	ENSDARG00000077237	rfx7a	ENSDARG00000075964	plekhg4	181 174	141 136	0.367	0.794	yes
7	ENSDARG00000003495	madd	ENSDARG00000077237	rfx7a	189 394	93 397	-0.327	0.367	no
8	ENSDARG00000089901	ENSDARG00000089901	ENSDARG00000093392	ENSDARG00000093392	5 362	0	3.99	4.05	yes
8	ENSDARG00000090721	prdm2a	ENSDARG00000016830	rimkla	8 466	3 878	5.12	1.21	yes
8	ENSDARG00000068966	tnfrsf14	ENSDARG00000089901	ENSDARG00000089901	12 147	84	3.80	3.99	yes
8	ENSDARG00000073847	rbm15	ENSDARG00000068787	slc6a17	66 101	63 540	6.20	1.11	yes
8	ENSDARG00000078216	eps15	ENSDARG00000079308	tor3a	82 309	37 898	0.349	0.528	yes
9	ENSDARG00000057262	cyp27a3	ENSDARG00000077643	lypd6b	132 562	118 882	-0.953	-1.99	yes

Table S23. Consecutive DEGs on the same chromosome whose start positions lie less than 200 kb apart, with the distance between start positions, the gap between gene bodies and the two effect sizes. Symbols follow Ensembl 112.

Table S24. Genes with an all-or-none count pattern

Gene	Sym	Ctrl1	Ctrl2	Ctrl3	Str1	Str2	Str3
ENSDARG00000096599	si:ch211-160d14.9	57	65	77	0	0	0
ENSDARG00000056765	ela2l	0	0	0	57	40	72
ENSDARG00000113858	ENSDARG00000113858	48	38	41	0	0	0
ENSDARG00000097247	ENSDARG00000097247	0	0	0	49	15	17
ENSDARG00000110845	ENSDARG00000110845	0	0	0	8	12	18
ENSDARG00000100771	ENSDARG00000100771	15	7	13	0	0	0
ENSDARG00000083597	dre-mir-22a	0	0	0	14	9	9
ENSDARG00000076264	zgc:195170	0	0	0	15	10	5
ENSDARG00000092025	si:dkey-121n8.8	0	0	0	6	7	12

Table S24. DEGs with zero counts in every library of one group and non-zero counts in every library of the other.

Table S25. Genomic dispersion of the enriched terms

Source	Statement	K	N chromosomes	Min distance bp	Largest chr span bp	Genes zero in one group	Single cluster	Members
Huang et al. 2019	Interpro: Interferon-induced 6-16 (IFI6/IFI27-like), up	3	1	5 758	17 380	0	yes	ENSDARG00000039243, si:dkey-188i13.7, zgc:123068
Huang et al. 2019	Interpro: Orange domain, up	4	4	—	—	0	no	ENSDARG00000006514, her4.2, her8.2, hey1
present analysis, all 275	GO:0005581 collagen trimer	7	6	2.98×10^6	2.98×10^6	1	no	COL16A1, col12a1a, col17a1a, col17a1b, col28a1b, col4a6, col8a2
present analysis, all 275	GO:0005882 intermediate filament	6	6	—	—	0	no	krt17, krt4, krt5, si:dkey-183i3.5, zgc:136930, zgc:92380
present analysis, all 275	GO:0045111 intermediate filament cytoskeleton	6	6	—	—	0	no	krt17, krt4, krt5, si:dkey-183i3.5, zgc:136930, zgc:92380
present analysis, all 275	GO:0005604 basement membrane	5	5	—	—	1	no	col17a1a, col4a6, col8a2, lamb2l, loxl2b
present analysis, all 275	GO:0045595 regulation of cell differentiation	9	7	873 116	6.40×10^6	0	no	bhlhe41, her4.3, her8.2, hey1, id1, loxl2b, metrn, neurod6a, nog2
present analysis, all 275	GO:0050767 regulation of neurogenesis	7	6	5.53×10^6	5.53×10^6	0	no	bhlhe41, her4.3, her8.2, hey1, id1, metrn, neurod6a
present analysis, all 275	GO:0030216 keratinocyte differentiation	3	3	—	—	0	no	krt4, krt5, zgc:136930
present analysis, all 275	GO:0045109 intermediate filament organization	3	3	—	—	0	no	krt4, krt5, zgc:136930
present analysis, all 275	GO:0060284 regulation of cell development	7	6	5.53×10^6	5.53×10^6	0	no	bhlhe41, her4.3, her8.2, hey1, id1, metrn, neurod6a
present analysis, all 275	GO:0006956 complement activation	3	2	4.37×10^6	4.37×10^6	0	no	C7 (1 of many), si:dkey-8k3.2, st14b
present analysis, all 275	GO:0051960 regulation of nervous system development	7	6	5.53×10^6	5.53×10^6	0	no	bhlhe41, her4.3, her8.2, hey1, id1, metrn, neurod6a
present analysis, all 275	GO:0030020 extracellular matrix structural constituent conferring tensile strength	4	3	2.98×10^6	2.98×10^6	1	no	COL16A1, col17a1a, col4a6, col8a2
present analysis, all 275	GO:0048935 peripheral nervous system neuron development	3	2	9 940	9 940	0	no	cdk5, her4.2, her4.3
present analysis, all 275	GO:0048934 peripheral nervous system neuron differentiation	3	2	9 940	9 940	0	no	cdk5, her4.2, her4.3
present analysis, all 275	GO:0002252 immune effector process	4	3	4.37×10^6	4.37×10^6	0	no	C7 (1 of many), dysf, si:dkey-8k3.2, st14b
present analysis, all 275	GO:0008544 epidermis development	4	4	—	—	1	no	col17a1a, krt4, krt5, zgc:136930
present analysis, after exclusion	GO:0005581 collagen trimer	6	5	2.98×10^6	2.98×10^6	0	no	COL16A1, col12a1a, col17a1b, col28a1b, col4a6, col8a2
present analysis, after exclusion	GO:0045595 regulation of cell differentiation	9	7	873 116	6.40×10^6	0	no	bhlhe41, her4.3, her8.2, hey1, id1, loxl2b, metrn, neurod6a, nog2
present analysis, after exclusion	GO:0050767 regulation of neurogenesis	7	6	5.53×10^6	5.53×10^6	0	no	bhlhe41, her4.3, her8.2, hey1, id1, metrn, neurod6a

present analysis, after exclusion	GO:0060284 regulation of cell development	7	6	5.53×10^6	5.53×10^6	0	no	bhlhe41, her4.3, her8.2, hey1, id1, metrn, neurod6a
present analysis, after exclusion	GO:0051960 regulation of nervous system development	7	6	5.53×10^6	5.53×10^6	0	no	bhlhe41, her4.3, her8.2, hey1, id1, metrn, neurod6a

Table S25. For each enriched term, the number of member genes, the number of chromosomes they occupy and the smallest distance between two of them. A term carried by genes on one chromosome within a short interval is not an independent observation of that many genes.

Table S26. Multiplicity correction across the marker panels

Panel	N markers	Diff	T	P	P bonferroni	P BH
radial_glia	8	0.258	3.01	0.040	0.474	0.282
epidermis	9	-1.86	-2.84	0.047	0.564	0.282
erythrocyte	2	-0.499	-1.81	0.145	1	0.580
microglia	5	0.149	1.31	0.261	1	0.632
oligodendrocyte	5	-0.253	-1.30	0.263	1	0.632
olfactory_epithelium	3	-0.159	-0.886	0.426	1	0.730
skeletal_muscle	5	-0.144	-0.774	0.482	1	0.730
pineal	5	-0.645	-0.765	0.487	1	0.730
endothelium	4	-0.078	-0.485	0.653	1	0.870
lens_eye	2	-0.753	-0.293	0.784	1	0.941
neuron	12	-0.00579	-0.106	0.921	1	0.968
cartilage_bone	4	-0.028	-0.043	0.968	1	0.968

Table S26. Benjamini–Hochberg correction applied across the twelve panels of Table S22. No panel survives at FDR < 0.05.

Table S27a. Epidermal score, leave-one-library-out

Dropped	Diff	P
ctrl1	-2.29	0.037
ctrl2	-1.42	0.109
ctrl3	-1.86	0.114
str1	-2.05	0.084
str2	-2.08	0.079
str3	-1.44	0.113

Table S27a. The epidermal composite recomputed with each library omitted in turn.

Table S27b. Epidermal score, leave-one-marker-out

Dropped	Diff	P
krt91	-1.72	0.051
krt97	-1.54	0.061
krt8	-2.09	0.046
tp63	-1.95	0.043
anxa1a	-2.00	0.043
s100a10b	-2.08	0.042
krt1-19d	-1.90	0.045
epcam	-1.61	0.059
muc5.1	-1.82	0.047

Table S27b. The epidermal composite recomputed with each marker gene omitted in turn.

Table S28. Model with an orthogonal composition term

Contrast	Set	N	Direction	T	P	N DEG
group (stress vs control)	GR_INDUCED_EACHUS	2 443	Down	-3.08	0.00206	246
group (stress vs control)	GR_REPRESSED_EACHUS	1 951	Up	3.15	0.00162	246
epithelial content, within groups	GR_INDUCED_EACHUS	2 443	Up	1.62	0.105	16
epithelial content, within groups	GR_REPRESSED_EACHUS	1 951	Down	-0.816	0.414	16

Table S28. Tests of the two glucocorticoid sets against the group factor and against the epidermal score centred within groups. Centring within groups makes the covariate orthogonal to the group factor by construction; the test therefore concerns the within-group component of composition only.

Table S29. Expression-matched null for the positional clustering

N iterations	Matching	Observed pairs	Observed same direction	Null pairs mean	Null pairs 95	Null same mean	Null same 95	Empirical p pairs	Empirical p same direction
2 000	expression decile	48	37	15.22	[8.0, 23.0]	7.84	[3.0, 14.0]	0	0

Table S29. Two thousand random gene sets drawn to match the DEG list decile by decile on mean normalised expression, counted by the same function that produced the observed values.

Table S30. Mirror null for the glucocorticoid gene sets

N iterations	Set sizes	Observed	Null mirror both significant	Null mirror same orientation	Empirical p mirror any orientation	Empirical p mirror same orientation	Null worse p median	Null worse p le observed
2 000	[2443, 1951]	{'induced': {'direction': 'Down', 'p': 0.0014623498625153206}, 'repressed': {'direction': 'Up', 'p': 0.0015374413534325517}}	0	0	0	0	0.936	0

Table S30. Two thousand disjoint pairs of random gene sets of the same sizes as the two glucocorticoid sets, drawn from the same ranked statistics and tested by the same competitive procedure.

Table S31. Reliability of the newly identified genes

N new testable	N agree	Pct	Baseline pct	Binom p vs baseline	Spearman	N with pub adj. p	N pub adj. p lt 010	N pub adj. p lt 025	Median pub adj. p new	Median pub adj. p background	Flagged new pct	Flagged old pct	Fisher p flags	Median abs l2fc new	Median abs l2fc old
163	148	90.80	79.12	5.24×10^{-5}	0.867	159	24	61	0.542	1.000	10.90	8.90	0.682	0.930	1.21

Table S31. Agreement in direction between the two analyses for the genes significant here but not in the published list, against a background of expressed genes significant in neither.

Table S32. Decomposition of the increase in the number of DEGs

Quantity	Value
n_absent_from_huang	11
n_filtered_by_huang	4
n_tested_above_threshold	159
huang_genes_with_padj	21235
our_genes_with_padj	20863
basemean_ratio_newDEGs	1.4587369523048794
lfcSE_ratio_newDEGs	0.8974014441456651
lfcSE_smaller_newDEGs	108/159
basemean_ratio_all	1.9085664871112444
lfcSE_ratio_all	0.7784795070308356
lfcSE_smaller_pct_all	84.84485006518905
counterfactual_huangSE	238
counterfactual_ourSE	262
n_both_tested	19175
n_common	19175
gain_total	122
gain_precision	341
gain_estimates	98
medFC_ourONLY_ours	0.929832102097375
medFC_ourONLY_pub	0.618315331
medFC_pubsig_ours	0.940335993656973
medFC_pubsig_pub	1.2095374445
medFC_all_ours	0.124685600755437
medFC_all_pub	0.149427805
counterfactual: published FC / published SE	140
counterfactual: published FC / our SE	481
counterfactual: our FC / published SE	238
counterfactual: our FC / our SE	262

Table S32. Where the additional genes come from, and a counterfactual recomputation crossing the effect-size estimates of each analysis with the standard errors of the other. The counterfactual mixes quantities from two pipelines under a normal approximation and is a decomposition, not a prediction.

Table S33. Glucocorticoid sets after removal of both flagged classes

Analysis	N dropped	Set	N	Direction	T	P
all genes	0	GR_INDUCED_EACHUS	2 443	Down	-3.18	0.00146
all genes	0	GR_REPRESSED_EACHUS	1 951	Up	3.17	0.00154
28 non-neural / all-or-none	28	GR_INDUCED_EACHUS	2 442	Down	-3.22	0.00128
28 non-neural / all-or-none	28	GR_REPRESSED_EACHUS	1 948	Up	3.28	0.00105
genomic blocks only	64	GR_INDUCED_EACHUS	2 434	Down	-3.35	0.00082
genomic blocks only	64	GR_REPRESSED_EACHUS	1 937	Up	3.18	0.00146
both classes	85	GR_INDUCED_EACHUS	2 433	Down	-3.36	0.00078
both classes	85	GR_REPRESSED_EACHUS	1 937	Up	3.20	0.00137
counts	—	n_block_members	66	—	—	—
counts	—	n_block_excl_her4	64	—	—	—
counts	—	n_union	85	—	—	—
counts	—	n_remaining	190	—	—	—

Table S33. Competitive tests after removal of the reassigned genes, of the same-direction members of the positional blocks, and of the union of the two.

Table S34. Symbols that differ between the two annotation releases

Gene	Symbol Ensembl 112	Symbol Ensembl 89 used by in [1]	Log2FoldChange	Adj. p
ENSDARG00000074345	si:ch211-214b16.3	si:ch211-214b16.4	3.28	3.89×10^{-30}
ENSDARG00000068966	tnfrsf14	si:ch211-261n11.7	3.80	3.67×10^{-29}
ENSDARG00000091847	LOC110437747	si:ch211-181d7.1	6.73	4.74×10^{-23}
ENSDARG00000096508	(unnamed)	BX322618.1	-2.34	1.22×10^{-20}
ENSDARG00000104899	si:dkey-242k1.4	CR931813.2	-1.60	3.41×10^{-19}
ENSDARG00000068621	nlrc11	si:ch211-181d7.3	8.65	1.75×10^{-13}
ENSDARG00000069189	(unnamed)	si:dkey-242h9.3	3.08	2.26×10^{-10}
ENSDARG00000104004	LOC101883440	si:dkey-146c18.2	-5.06	3.26×10^{-10}
ENSDARG00000089901	(unnamed)	si:ch211-261n11.8	3.99	5.04×10^{-10}
ENSDARG00000096599	si:ch211-160d14.9	AL928650.3	-8.50	6.56×10^{-9}
ENSDARG00000091607	LOC101884284	si:ch211-272b8.6	2.14	2.23×10^{-7}
ENSDARG00000038863	babam2	bre	0.728	1.02×10^{-6}
ENSDARG00000013997	(unnamed)	ern1	-1.05	1.14×10^{-6}
ENSDARG00000017489	ifi27.1	zgc:123068	2.11	1.38×10^{-6}
ENSDARG00000096046	(unnamed)	BX649307.1	1.04	5.33×10^{-6}
ENSDARG00000092077	(unnamed)	si:dkey-147f3.4	5.55	1.30×10^{-5}
ENSDARG00000103538	(unnamed)	BX571954.2	-4.38	2.35×10^{-5}
ENSDARG00000030107	syne2a	si:ch211-207i1.2	-1.80	3.22×10^{-5}
ENSDARG00000097247	(unnamed)	BX322555.3	7.17	3.50×10^{-5}
ENSDARG00000037587	synpra	SYNPR (1 of many)	0.692	3.75×10^{-5}
ENSDARG00000070957	mfsd11	im:7160594	0.927	4.18×10^{-5}
ENSDARG00000038424	c4b	si:dkey-8k3.2	1.33	4.30×10^{-5}
ENSDARG00000070601	rassf5	RASSF5	0.874	6.90×10^{-5}
ENSDARG00000074844	(unnamed)	CABZ01061495.1	1.21	7.95×10^{-5}
ENSDARG00000005216	megf6	zgc:158328	1.56	9.54×10^{-5}
ENSDARG00000087012	(unnamed)	BX004816.4	5.69	0.00014
ENSDARG00000070621	zgc:101564	FAM72B	1.01	0.00028
ENSDARG00000101702	chrna7a	chrna7	-1.74	0.00032
ENSDARG00000029232	(unnamed)	FO704779.1	-0.901	0.00036
ENSDARG00000071029	fbxo44.9	si:dkey-147f3.4	2.02	0.00049
ENSDARG00000036848	(unnamed)	slc43a2a	-0.773	0.00051
ENSDARG00000056729	her4.5	her4.2	2.76	0.00051
ENSDARG00000097574	si:ch211-160m12.2	CR847898.4	-2.32	0.00079
ENSDARG00000009194	col16a1	COL16A1	0.992	0.00079
ENSDARG00000031588	si:ch211-39f2.3	si:dkey-239b22.1	-4.30	0.00202
ENSDARG00000090429	ccdc178	si:dkeyp-7a3.1	-0.983	0.00248
ENSDARG00000042055	niban1a	fam129aa	-4.77	0.0047
ENSDARG00000100771	(unnamed)	si:ch211-160d14.15	-5.98	0.00551
ENSDARG00000032856	ccdc120b	ccdc120	-0.836	0.00551
ENSDARG00000093494	(unnamed)	si:ch211-217k17.9	-6.18	0.0059
ENSDARG00000052626	LOC100005723	AL954655.1	-4.60	0.00625
ENSDARG00000057262	cyp27a3	si:dkey-91i10.3	-0.953	0.00637
ENSDARG00000010764	dhrs12la	flj13639	-0.650	0.00657
ENSDARG00000061602	(unnamed)	ssx2ipb	-0.630	0.00679
ENSDARG00000097556	cyp8b1.2	cyp8b2	-3.17	0.00988
ENSDARG00000068840	ppt2a.1	zgc:66024	5.49	0.011
ENSDARG00000088810	prox3	prox1b	0.682	0.014
ENSDARG00000090340	fam83ga	si:dkey-27o4.1	2.78	0.017
ENSDARG00000104387	slc4a5b	slc4a5	0.896	0.018
ENSDARG00000105689	(unnamed)	znf1009	0.776	0.019
ENSDARG00000102679	si:ch73-158p21.7	FP074874.2	-0.729	0.019
ENSDARG00000057121	c7b	C7 (1 of many)	-0.887	0.019
ENSDARG00000059369	(unnamed)	si:ch1073-15f12.3	-4.28	0.020
ENSDARG00000055504	cd68	si:ch211-212k18.7	0.968	0.020
ENSDARG00000101556	fam111.2	si:ch1073-475a24.1	3.06	0.021
ENSDARG00000092042	(unnamed)	AL935153.1	4.03	0.021
ENSDARG00000088549	si:ch1073-390k14.1	CU611050.1	-1.16	0.022
ENSDARG00000103413	rtbdn	zgc:109949	0.482	0.022
ENSDARG00000103218	(unnamed)	BX927130.3	-2.41	0.023
ENSDARG00000077570	(unnamed)	cdh16	-1.83	0.023
ENSDARG00000039963	fgfbp1a	fgfbp1b	1.47	0.024
ENSDARG00000100598	cdh19	CDH19	-1.15	0.025
ENSDARG00000093392	(unnamed)	CT583651.1	4.05	0.025
ENSDARG00000077237	rfx7a	rfx7	0.367	0.030
ENSDARG00000103518	(unnamed)	abhd8a	-0.501	0.031
ENSDARG00000097238	ba1l	hbba1	-0.602	0.032
ENSDARG00000093823	(unnamed)	si:ch211-102c2.8	-1.24	0.038
ENSDARG00000078389	ifi27.4	si:dkey-188i13.7	1.46	0.040
ENSDARG00000105651	(unnamed)	BX323060.3	0.888	0.045
ENSDARG00000032426	coq8ab	COQ8A (1 of many)	1.92	0.046

Table S34. DEGs carrying a different symbol in Ensembl 112 and in the Ensembl 89 release used by [1]. Comparison between the analyses was made on stable identifiers throughout.

Table S34b. Symbols reassigned to a different gene

Obsolete symbol	Denoted in in [1]	Denotes now
si:ch211-181d7.1	ENSDARG00000091847	ENSDARG00000088274
her4.2	ENSDARG00000056729	ENSDARG00000094426
fgfbp1b	ENSDARG00000039963	ENSDARG00000091660
hbba1	ENSDARG00000097238	ENSDARG00000089087

Table S34b. Symbols that denote one gene in the annotation used by [1] and a different gene in Ensembl 112. A comparison made by symbol rather than identifier would silently substitute one locus for the other.

Table S34c. Symbols attached to more than one identifier in the source table

Ensembl	Symbol in in [1]	Log_2FC in [1]	Adj. p in [1]	Symbol Ensembl 112	N ids sharing symbol in in [1]
ENSDARG00000091847	si:ch211-181d7.1	9.43	1.46×10^{-11}	LOC110437747	2
ENSDARG00000092077	si:dkey-147f3.4	5.28	0.00181	—	2
ENSDARG00000056729	her4.2	1.41	0.020	her4.5	2
ENSDARG00000089087	hbba1	-0.542	0.022	hbba1	2
ENSDARG00000039963	fgfbp1b	1.48	0.027	fgfbp1a	2
ENSDARG00000105689	znf1009	0.884	0.065	—	3
ENSDARG00000071029	si:dkey-147f3.4	2.62	0.073	fbxo44.9	2
ENSDARG00000059093	ank1a	-0.450	0.237	ank1a	2
ENSDARG00000097238	hbba1	-0.533	0.695	ba1l	2
ENSDARG00000090429	si:dkeyp-7a3.1	-0.597	0.964	ccdc178	2

Table S34c. DEGs whose symbol in the supplementary table of [1] is shared with at least one other Ensembl identifier in that same table. Across the whole table 289 symbols are shared in this way, covering 762 genes.

Table S35. Cross-check of the RT-qPCR panel of the original study

Symbol in supplement3	Accession	Role	Gene	Resolution	Note	Symbol Ensembl 112	Our log_2FC	Our adj. p	Our DEG	Pub log_2FC	Pub adj. p	Pub DEG	On exclusion list
ache	NM_131846	target	ENSDARG00000031796	symbol in [1]	—	ache	0.002	1.000	0	0.012	1.000	0	no
nr3c1	NM_001020711	target	ENSDARG00000025032	symbol in [1]	—	nr3c1	0.041	1.000	0	0.008	1.000	0	no
hsd11b2	NM_212720	target	ENSDARG00000001975	symbol in [1]	—	hsd11b2	-0.344	1.000	0	-0.126	1.000	0	no
npy	NM_131074	target	ENSDARG00000036222	symbol in [1]	—	npy	-0.060	1.000	0	-0.119	1.000	0	no
loxl2b	NM_001080626	target	ENSDARG00000044074	symbol in [1]	—	loxl2b	-2.80	0.00242	1	-2.86	1.05×10^{-7}	1	no
cdk5	NM_131719	target	ENSDARG00000056683	symbol in [1]	—	cdk5	0.529	0.044	1	0.813	0.0028	1	no
chrna7	NM_201219	target	ENSDARG00000101702	symbol in [1]	—	chrna7a	-1.74	0.00032	1	-2.02	0.023	1	no
gpx1a	NM_001007281	target	ENSDARG00000018146	symbol in [1]	—	gpx1a	-0.667	0.043	1	-0.587	3.65×10^{-5}	1	no
jun	NM_199987	target	ENSDARG00000043531	symbol in [1]	—	jun	-0.693	0.022	1	-0.699	0.023	1	no
igf1bp2a	NM_131458	target	ENSDARG00000052470	corrected symbol	—	igfbp2a	0.306	0.703	0	0.527	0.00501	1	no
acyp1	NM_001122849	target	ENSDARG00000060126	symbol in [1]	—	acyp1	4.57	1.75×10^{-13}	1	4.27	2.55×10^{-6}	1	no
col8a2	NM_001082901	target	ENSDARG00000060893	symbol in [1]	—	col8a2	1.45	0.028	1	1.50	0.046	1	no
ela2l	NM_199886	target	ENSDARG00000056765	symbol in [1]	—	ela2l	8.30	3.72×10^{-8}	1	7.15	2.19×10^{-5}	1	yes
nog2	NM_130992	target	ENSDARG00000043066	symbol in [1]	—	nog2	0.734	0.013	1	0.728	0.029	1	no
nub1	NM_001113580	target	ENSDARG00000061417	symbol in [1]	—	nub1	0.683	0.052	0	2.24	0.00028	1	no
hey1	NM_212561.1	target	ENSDARG00000070538	symbol in [1]	—	hey1	0.725	9.54×10^{-5}	1	0.785	0.00012	1	no
her6	NM_131079.2	target	ENSDARG00000006514	symbol in [1]	—	her6	0.391	0.345	0	0.484	0.010	1	no
her4.2	NM_131090.4	target	—	symbol in [1]	ambiguous in [1]: 2 identifiers ['ENSDARG00000056729', 'ENSDARG00000094426']	—	—	—	—	—	—	—	no
her8.2	NM_001166166.1	target	ENSDARG00000069675	symbol in [1]	—	her8.2	1.45	0.0043	1	2.05	0.00578	1	no
ef1a	NM_131263	reference	ENSDARG00000020850	current symbol	—	eef1a1l1	-0.061	1.000	0	-0.062	1.000	0	no

Table S35. The nineteen genes of Supplement 3 of [1] with the reference gene, resolved to stable identifiers through the supplementary table of [1] rather than by symbol, with the route recorded for each. The entry printed as igf1bp2a is igfbp2a; chrna7 is chrna7a in the current annotation; her4.2 cannot be resolved because that symbol is attached to two identifiers in the table of [1]

Table S18b. The zebrafish expression record behind each tissue assignment

Gene	Symbol	Evidence tier	Adult zebrafish record	Neural record	Source consulted	Note
ENSDARG00000017624	krt4	A, adult zebrafish expression	trunk integument (Adult)	none recorded	Han et al. 2020, ZFIN ZDB-GENE-000607-83	
ENSDARG00000058371	krt5	B, zebrafish, not adult or not resolved	not resolved	none in the ZFIN summary	COKUS19 (embryos); ZFIN summary: periderm, integument	
ENSDARG00000094041	krt17	B, zebrafish, not adult or not resolved	not resolved	none in the ZFIN summary	COKUS19 (embryos, periderm-preferred)	
ENSDARG00000069415	col17a1a	B, zebrafish, not adult or not resolved	cornea, by antibody labelling	none recorded	KIM10 (embryos and larvae); Oliver et al. 2016 (antibody)	the adult record is antibody-based and cannot separate col17a1a from col17a1b; ZFIN lists col17a1b among the former names of col17a1a
ENSDARG00000052705	pkp1b	B, zebrafish, not adult or not resolved	none recorded	none recorded	Thisse et al. 2004, embryonic in situ screen	enveloping layer, otic vesicle, peripheral olfactory organ, pharyngeal arch
ENSDARG00000069888	cldna	C, record includes adult neural tissue	integument and brain (Adult)	adult brain recorded	Baltzegar et al. 2013 adult tissue panel; Newman et al. 2019	
ENSDARG00000009544	cldnb	C, record includes adult neural tissue	epidermis (Adult, in situ); brain in an eleven-organ panel	adult brain recorded	Li et al. 2020; Clelland and Kelly 2010	
ENSDARG00000009978	icn	B, zebrafish, not adult or not resolved	transcript reported to persist into adulthood, not localised	none recorded	HSIAO03 (embryos)	ictacalcin, an S100-family protein, not an antimicrobial peptide
ENSDARG00000055514	icn2	B, zebrafish, not adult or not resolved	not resolved	none in the ZFIN summary	ZFIN summary: integument; HSIAO03 does not cover this gene	the assignment follows the paralogue icn
ENSDARG00000021866	upk1a	B, zebrafish, not adult or not resolved	none recorded	none recorded	Jubber et al. 2023 (cloaca 96 hpf, pronephric duct 120 hpf)	urinary tract, not epidermis
ENSDARG00000102750	cdh1	C, record includes adult neural tissue	brain, spinal cord neuroepithelium, cerebellum (Adult)	adult brain recorded	Luo et al. 2020 (glioma model); Klatt Shaw et al. 2021 (spinal cord transection); Jung et al. 2013 (cerebellum glioma)	adult neural records come from tumour and injury models; the record cannot discriminate origin either way
ENSDARG00000058556	muc5.2	A, adult zebrafish expression	gill lamellae (Adult)	none recorded	Jevtov et al. 2014, ZDB-FIG-141211-3	
ENSDARG00000044655	st14b	D, no zebrafish expression data	none	none	ZFIN reports no expression data for this gene	assignment rests on orthology to human ST14
ENSDARG00000103917	znf185	B, zebrafish, not adult or not resolved	none recorded	none recorded	Rodriguez et al. 2015 (Day 4); Hong et al. 2010 (Shield)	
ENSDARG00000056765	ela2l	B, zebrafish, not adult or not resolved	not resolved	none recorded	ZFIN: exocrine pancreas, liver	records of ela3l must not be read as ela2l
ENSDARG00000033832	ampd1	B, zebrafish, not adult or not resolved	none recorded	none recorded	Thisse et al. 2004 (ZDB-FIG-051107-206)	myotome and head musculature, embryonic
ENSDARG00000052708	tnni1b	B, zebrafish, not adult or not resolved	none recorded	none recorded	Fu et al. 2009; Cai et al. 2018	also cardiovascular; not specific to skeletal muscle
ENSDARG00000079393	tmprss15	D, no zebrafish expression data	none	neural group in the Daniocell larval atlas	ZFIN reports no anatomy term for this gene	assignment rests on orthology to mammalian TMPRSS15
ENSDARG00000044280	opn1mw2	C, record includes adult neural tissue	gill, heart, integument, nervous system, testis	nervous system recorded	ZFIN expression summary; adult opsin survey of Davies et al. 2015	attribution to retina alone is not supported
ENSDARG00000042850	nphs2	A, adult zebrafish expression	kidney and podocyte (Adult)	none recorded	Chen et al. 2016; Anderson et al. 2015	symbol changed from nphs2l

Table S18b. For each of the twenty genes set aside on annotation grounds, the zebrafish expression record that the assignment rests on, graded as defined in Section 4.7. Three genes carry expression in the attributed non-neural tissue in adult fish with no neural record (tier A); eleven carry zebrafish evidence that is embryonic, larval or not resolved to a stage (tier B); for four the record also includes adult brain or nervous system, so that it cannot discriminate the origin of a transcript found in a brain sample (tier C); and for two there is no zebrafish expression annotation at all (tier D). The eight genes set aside on the presence/absence pattern carry no expression evidence and are not listed here. Records were read from ZFIN on 23 October 2026; where a per-gene stage table could not be retrieved in full, the entry reads “not resolved” rather than being filled by inference. Table S19d repeats the exclusion analysis for the nested tiers.

Table S36. The six stressors of the source protocol and the level at which each was delivered

Stressor	Wording in the source	Level of delivery	Determinable from the source	Comment
Overcrowding	*“fish were confined by mesh dividers in their home tank for 50 minutes”*	Group, in the home tank	Yes	Crowding is a property of a group; the plural and the home tank are both explicit.
Shallow water	*“fish swam in 1 cm deep water for 2 minutes”*	Not determinable	No	Neither the vessel nor the number of fish treated at one time is stated.
Social isolation	*“individuals were confined in 250 ml fish water without visual contact of conspecifics for 50 minutes”*	Individual fish, outside the home tank	Yes	Explicitly individual, and the defining feature of the stressor is the absence of conspecifics.
Object chase	*“chased with a handle of a net in their home tank for 20 minutes”*	In the home tank; number of fish chased at one time not stated	Partly	The location is explicit, the unit is not.
Restraint	*“individuals were separated by mesh dividers in their home tank so that they touched the dividers when the changed orientation [sic], for 20 minutes”*	Individual fish, within the home tank	Yes	Delivered to individual fish, but to all occupants of a tank at the same time and in their own tank.
Tank change	*“netting between two tanks, 8 times”*	Group, between two tanks	Yes	The home tank is therefore not fixed for the stressed group.

Table S36. Each stressor as described in the Methods of [1], quoted verbatim, with the level at which it was delivered. Two stressors were delivered to individual fish, two to a group, and for two the number of fish treated at one time is not stated. The level of delivery does not determine the experimental unit, which is set by the level at which the treatment was allocated: the source states that *“Control animals were not handled during this time”*, so the control and stressed fish of a cohort cannot have occupied the same tank, and the treatment was therefore allocated to a housing group rather than to individual fish within a tank. Five further features of the design cannot be recovered from the article, its supplementary files or the archive record: the number of tanks per condition; the assignment of fish to tanks; the vessel used for shallow water, and whether fish were treated singly or together; the number of fish chased simultaneously during the object chase; whether the stressed and control fish of a cohort were housed on the same rack.

Table S37. Tissue-marker panels against the two glucocorticoid gene sets

Marker panel	Genes tested	Gene set	Observed	Expected	P	P, BH-adjusted
neuron	12	GR-induced	2	1.14	0.316299	1
neuron	12	GR-repressed	0	0.91	1	1
radial glia	9	GR-induced	1	0.85	0.591396	1
radial glia	9	GR-repressed	3	0.68	0.0256271	0.666305
oligodendrocyte	7	GR-induced	1	0.66	0.501469	1
oligodendrocyte	7	GR-repressed	1	0.53	0.42318	1
microglia	5	GR-induced	1	0.47	0.39176	1
microglia	5	GR-repressed	0	0.38	1	1
epidermis	15	GR-induced	0	1.42	1	1
epidermis	15	GR-repressed	0	1.13	1	1
olfactory epithelium	5	GR-induced	1	0.47	0.39176	1
olfactory epithelium	5	GR-repressed	0	0.38	1	1
exocrine pancreas	6	GR-induced	0	0.57	1	1
exocrine pancreas	6	GR-repressed	0	0.45	1	1
erythrocyte	3	GR-induced	0	0.28	1	1
erythrocyte	3	GR-repressed	0	0.23	1	1
endothelium	4	GR-induced	1	0.38	0.328164	1
endothelium	4	GR-repressed	1	0.3	0.26977	1
pineal	5	GR-induced	0	0.47	1	1
pineal	5	GR-repressed	0	0.38	1	1
skeletal muscle	6	GR-induced	1	0.57	0.449339	1
skeletal muscle	6	GR-repressed	0	0.45	1	1
cartilage bone	6	GR-induced	0	0.57	1	1
cartilage bone	6	GR-repressed	0	0.45	1	1
lens eye	4	GR-induced	0	0.38	1	1
lens eye	4	GR-repressed	1	0.3	0.26977	1

Table S37. Membership of the twelve marker panels of Table S17 in the two knockout-derived glucocorticoid sets, with the count expected from the set sizes against a background of 25,812 tested genes and a hypergeometric test of over-representation. None of the fifteen epidermal markers belongs to either set, against 1.4 and 1.1 expected, and no panel survives correction across the twenty-six tests (smallest adjusted $P = 0.67$). A change in the epidermal share of a library therefore has no direct representation in the two sets. This bounds the mechanism rather than excluding it, since transcripts expressed in both skin and brain are not captured by a marker panel.
